# Metabolomic age and risk of 50 chronic diseases in community‐dwelling adults: A prospective cohort study

**DOI:** 10.1111/acel.14125

**Published:** 2024-02-21

**Authors:** Xianwen Shang, Jiahao Liu, Zhuoting Zhu, Xueli Zhang, Yu Huang, Shunming Liu, Wei Wang, Xiayin Zhang, Shuo Ma, Shulin Tang, Yijun Hu, Zongyuan Ge, Honghua Yu, Mingguang He

**Affiliations:** ^1^ Guangdong Eye Institute, Department of Ophthalmology, Guangdong Provincial People's Hospital, Guangdong Academy of Medical Sciences Southern Medical University Guangzhou China; ^2^ Guangdong Cardiovascular Institute Guangdong Provincial People's Hospital, Guangdong Academy of Medical Sciences Guangzhou China; ^3^ The Ophthalmic Epidemiology Department Centre for Eye Research Australia Melbourne Victoria Australia; ^4^ Department of Medicine, Royal Melbourne Hospital University of Melbourne Melbourne Victoria Australia; ^5^ Medical Research Institute, Guangdong Provincial People's Hospital, Guangdong Academy of Medical Sciences Southern Medical University Guangzhou China; ^6^ State Key Laboratory of Ophthalmology, Zhongshan Ophthalmic Center Sun Yat‐sen University Guangzhou China; ^7^ Medical Big Data Center, Guangdong Provincial People's Hospital, Guangdong Academy of Medical Sciences Southern Medical University Guangzhou China; ^8^ Monash e‐Research Center, Faculty of Engineering, Airdoc Research, Nvidia AI Technology Research Center Monash University Melbourne Victoria Australia; ^9^ Experimental Ophthalmology The Hong Kong Polytechnic University Hong Kong China

**Keywords:** cardiometabolic disorder, chronic kidney disease, chronic obstructive pulmonary disease, liver disease, metabolomic age, moderation analysis, oesophageal cancer

## Abstract

It is unclear how metabolomic age is associated with the risk of a wide range of chronic diseases. Our analysis included 110,692 participants (training: *n* = 27,673; testing: *n* = 27,673; validating: *n* = 55,346) aged 39–71 years at baseline (2006–2010) from the UK Biobank. Incident chronic diseases were identified using inpatient records, or death registers until January 2021. Predicted metabolomic age was trained and tested based on 168 metabolomics. Metabolomic age was linked to the risk of 50 diseases in the validation dataset. The median follow‐up duration for individual diseases ranged from 11.2 years to 11.9 years. After controlling for false discovery rate, chronological age‐adjusted age gap (CAAG) was significantly associated with the incidence of 25 out of 50 chronic diseases. After adjustment for full covariates, associations with 15 chronic diseases remained significant. Greater CAAG was associated with increased risk of eight cardiometabolic disorders (including cardiovascular diseases and diabetes), some cancers, alcohol use disorder, chronic obstructive pulmonary disease, chronic kidney disease, chronic liver disease and age‐related macular degeneration. The association between CAAG and risk of peripheral vascular disease, other cardiac diseases, fracture, cataract and thyroid disorder was stronger among individuals with unhealthy diet than in those with healthy diet. The association between CAAG and risk of some conditions was stronger in younger individuals, those with metabolic disorders or low education. Metabolomic age plays an important role in the development of multiple chronic diseases. Healthy diet and high education may mitigate the risk for some chronic diseases due to metabolomic age acceleration.

## Graphical abstract

Accelerated metabolomic age was independently associated with an increased risk of 15 chronic conditions including 8 cardiometabolic disorders, some cancers, alcohol use disorder, chronic obstructive pulmonary disease, chronic kidney disease, chronic liver disease and age‐related macular degeneration. Healthy diet and higher education may mitigate the risk for some chronic diseases due to metabolomic age acceleration.

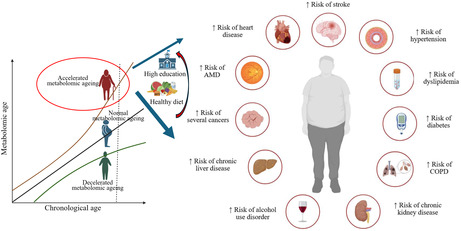

**Abbreviations**
BMI: body mass index
CAAG: chronological age‐adjusted age gap
CHD: coronary heart disease
CI: confidence interval
CKD: chronic kidney disease
CMD: cardio metabolic disorder
COPD: chronic obstructive pulmonary disease
CVD: cardiovascular disease
GRS: genetic risk score
HR: hazard ratio

## 1 INTRODUCTION

Chronological age is one of the most important risk factors for chronic disorders and mortality (Beard et al., 2016; López‐Otín et al., 2013). People with the same chronological age may differ in biological health, and biological ageing may be a better predictor for health (Horvath, 2013; Jylhävä et al., 2017). Biological age has been developed based on omics including genomics, telomere length, transcriptomics and proteomics and it has been shown to be a strong predictor of chronic diseases and mortality (Jylhävä et al., 2017; Kuo et al., 2022). In the global ageing population, it is important to understand biological age for the prevention of chronic diseases and promoting healthy ageing.

Metabolism plays an important role not only in the development of metabolic disorders but also in cardiovascular disease (CVD), neurogenerative disorders, musculoskeletal disorders, mental disorders, respiratory conditions and cancers (Amorim et al., 2022; Eckel et al., 2018; Lumsden et al., 2022). Metabolomic state added predictive value even over established clinical variables in the development of multiple chronic diseases (Buergel et al., 2022). A cohort study from Germany (discovery cohort: 2162 participants and replication cohort: 724 women) identified multiple metabolomics that were highly correlated with chronological age (Yu et al., 2012). A cohort study of 2239 participants from the UK showed that metabolomic age developed based on metabolic profiles was associated with the prevalence of obesity, diabetes, heavy alcohol use and depression (Robinson et al., 2020). Several other studies have demonstrated that metabolomic age acceleration was associated with an increased risk of mortality (Deelen et al., 2019; Fischer et al., 2014), and CVD (van den Akker et al., 2020). As previous studies have focused on one or several diseases, whether metabolomic age acceleration is associated with the risk of a wide range of chronic diseases remains to be explored. These studies are also limited by the relatively small sample sizes or cross‐sectional design, while biological clock estimates using machine learning can be improved by increasing the training sample size (Zhang et al., 2019).

Using the UK Biobank, we aimed to develop metabolomic age based on metabolomics measured by nuclear magnetic resonance spectroscopy using machine learning. We then examined the association between metabolomic age and a wide range of individual chronic diseases. The interplay between metabolomic age and age, sex, diet and metabolic disorders for chronic diseases was then examined.

## 2 METHODS

### 2.1 Study population

The present study was based on the UK Biobank, which is a population‐based cohort of more than 500,000 participants aged 39–73 years at enrolment (Sudlow et al., 2015). Data on demographic factors, lifestyle and medical history were collected using self‐administered questionnaires from 502,505 participants out of approximately 9.2 million invited people. Details of the study design have been shown elsewhere (Sudlow et al., 2015).

The UK Biobank Study's ethical approval has been granted by the National Information Governance Board for Health and Social Care and the NHS North West Multicenter Research Ethics Committee (REC reference: 16/NW/0274). All participants provided informed consent through electronic signature at recruitment.

### 2.2 Ascertainment of diseases

Individual diseases were defined if participants reported that they had ever been told by a doctor that they had the disease (Field code: Table S1). Fifty major diseases (all important conditions of interest) such as cardiometabolic disorders (CMD) (including diabetes, coronary heart disease (CHD), heart failure, atrial fibrillation and stroke), cancer (including melanoma, lung cancer and stomach cancer), chronic obstructive pulmonary disease (COPD) and chronic kidney disease (CKD) were included in the analysis.

Inpatient data were used to identify additional disease cases at baseline. Inpatient hospital records were captured using the Hospital Episode Statistics database, the Scottish Morbidity Record, and the Patient Episode Database in England, Scotland and Wales (Sudlow et al., 2015). In the UK Biobank, the inpatient hospital data were available since 1997 (Sudlow et al., 2015). The international classification diseases codes for each of the 50 diseases are listed in Table S2. Incident cases of these diseases were defined using inpatient and mortality data. The onset date of diseases was defined as the earliest recorded date regardless of sources. Person‐years for each disease were calculated from the date of baseline assessment to the date of onset, date of death or the end of follow‐up (31 December 2020 for England and Wales and 31 January 2021 for Scotland), whichever came first.

### 2.3 Metabolomic profiling

In the UK Biobank, metabolomic profiles were measured according to the structure and chemical properties of molecules using a high‐throughput nuclear magnetic resonance‐based metabolic biomarker profiling platform (Würtz et al., 2017). EDTA plasma samples were collected from a randomly selected subset of 117,121 participants at baseline and 5000+ participants at the first repeat visit. Venous blood sampling was collected and transported to a central laboratory and stored in ultra‐low temperature archives. The measurements at baseline were used in our analysis. Measurements were conducted for 249 metabolic traits (168 concentrations and 81 ratios) including the lipoprotein lipids, fatty acids, amino acids, glycolysis, organic acids and nucleotides. Automated quality control was performed, and biomarker values substantially affected by interfering substances were removed (https://biobank.ctsu.ox.ac.uk/crystal/label.cgi?id=220) (Soininen et al., 2015; Würtz et al., 2017). Metabolomic levels were normalized with mean = 0 and standard deviation (SD) = 1.

### 2.4 Covariates

Demographic information on age, sex, ethnicity, education and annual household income was self‐reported. Sleep duration was assessed based on the question ‘About how many hours' sleep do you get in every 24 h?’ Physical activity was assessed using a short form of the International Physical Activity Questionnaire. Diet score was computed based on seven commonly eaten food groups with a higher score representing a healthier diet (Lourida et al., 2019). Healthy diet was defined as diet score ≥4 and unhealthy diet as diet score <4 (Lourida et al., 2019). Medication use for antihypertension, lipid‐lowering and glucose‐lowering was self‐reported. Body mass index (BMI) was calculated based on measured height and weight. A genetic risk score (GRS) for longevity was computed using 78 single‐nucleotide polymorphisms with a higher score representing longer longevity (Timmers et al., 2020).

### 2.5 Statistical analysis

**FIGURE 1:**
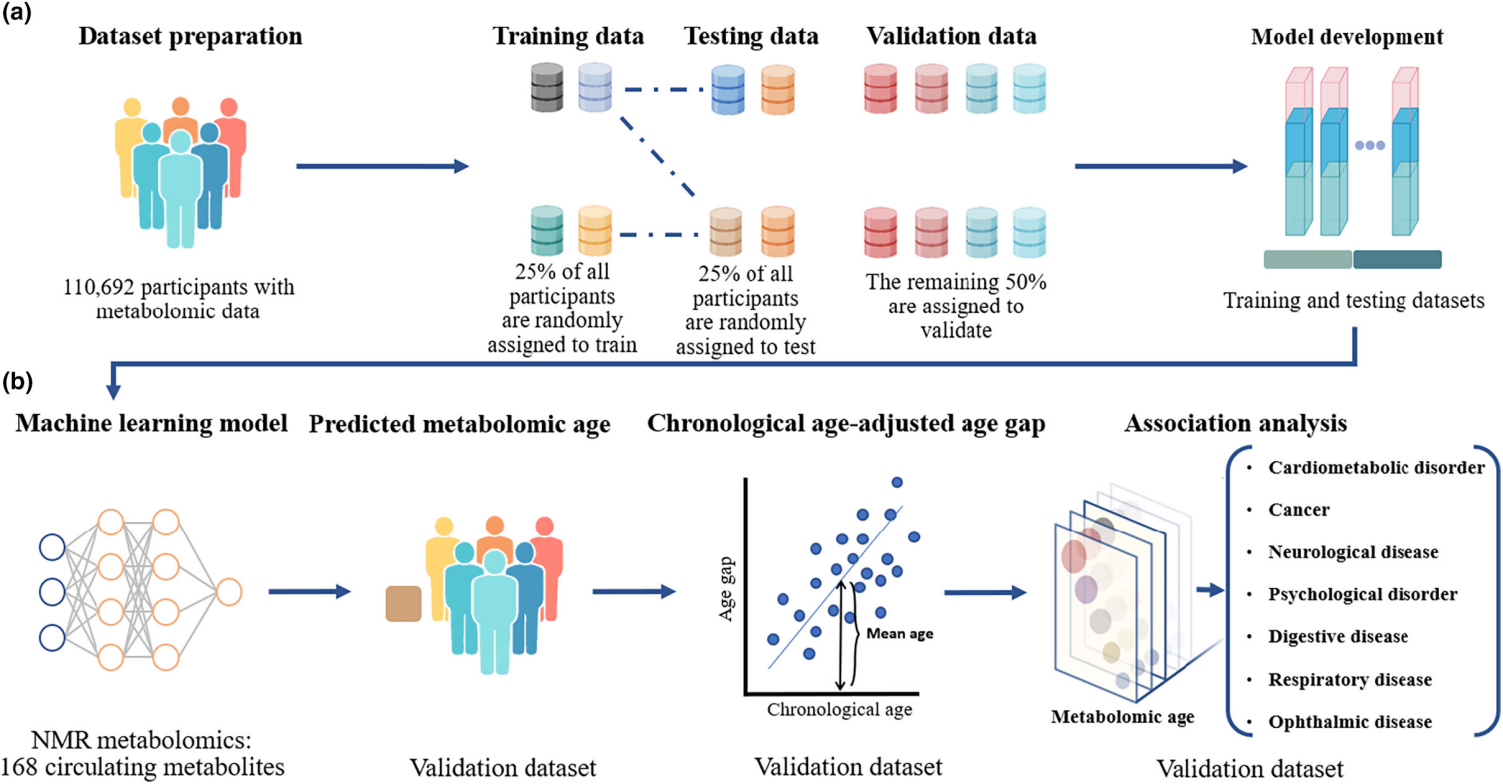
Flowchart for the development of metabolomic age and its association with the risk of chronic diseases (a) refers to the division of datasets; (b) refers to the development of metabolomic age and its linkage to chronic diseases.

We randomly selected 50% of the population with metabolomic data stratified by the assessment center to train (25% randomly selected participants) and test (the remaining 25% participants) the chronological age prediction model based on 168 metabolomic profiling concentrations. Data from the remaining 50% participants were used to develop metabolomic age and examine the association between metabolomic age and the risk of multiple diseases. Multiple linear regression models with the chronological age as the dependent variable were used to develop metabolomic age. We selected Gaussian family distribution when establishing prediction model using machine learning (Figure 1). The hyper‐parameters alpha and lambda specify the regularization strength and the regularization distribution between L1 (LASSO) and (ridge regression) L2 penalties, respectively. In this study, we used the R‐square to determine the best prediction performance.

Age gap was calculated by subtracting chronological age from metabolomic age. Given the age gap for individuals with different ages might represent different metabolomic ageing levels, chronological age‐adjusted age gap (CAAG) was calculated with the use of regression models (Willett et al., 1997). Baseline characteristics were expressed as frequency (%) or means ± SDs. ANOVA for continuous variables and chi‐square test for categorical variables were used to test the difference of characteristics by quintiles of CAAG.

The association between CAAG and incidence of each chronic disease was examined using the Cox proportional regression models. For each individual chronic disease, participants with the corresponding disease at baseline were excluded from the analysis. Three models were tested: (1) Model 1 was unadjusted; (2) Model 2 was adjusted for age, sex, ethnicity, education, household income, diet score, alcohol consumption, physical activity, smoking, sleep duration, fasting duration and GRS for longevity; (3) Model 3 was adjusted for Model 2 plus BMI, high cholesterol, hypertension, diabetes and antihypertensive, glucose‐lowering, and lipid‐lowering medications. CAAG was analysed in quintiles as well as a continuous variable (each year). Benjamin‐Hochberg's procedure was used to control the false discovery rate at a 5% level for multiple comparisons (Benjamini & Hochberg, 1995).

Sensitivity analysis of the association between CAAG and risk of individual diseases was conducted among individuals by excluding those developed the disease in the first year of follow‐up or by excluding those developed in the first 5 years of follow‐up. Whether associations between CAAG and chronic diseases were modified by age, sex, education, diet quality, metabolic disorders or GRS for longevity was tested using the Cox proportional regression models.

Percentages of individuals with missing data in BMI, physical activity, income and education were 2%, 19%, 14% and 1%, respectively. Given that individuals with missing data in outcome/exposure variables were excluded from the analysis, multiple imputations for missing data in covariates only using the fully conditional specification method were conducted to create 10 imputed datasets.

Data analyses were conducted using SAS 9.4 for Windows (SAS Institute Inc.) and all *p*‐values were two‐sided with statistical significance set at <0.05.

## 3 RESULTS

### 3.1 Population selection

Individuals with no data on metabolomic profiles (*n* = 391,785), or with missing data on inpatient data (*n* = 18) were excluded from the analysis. We included 110,692 participants in the final analysis. The analysis for the association between metabolic age and chronic diseases was conducted in the validation dataset (*n* = 55,346, 54.1% female, aged 39–71 [mean ± SD: 56.5 ± 8.1] years at baseline).

### 3.2 Metabolomic age and chronological age

In the machine learning analysis, the LASSO and ridge regression model with *α* = 0.2 and *λ* = 0.0001 showed the best prediction performance in the training and testing datasets. The algorism was then used to predict metabolomic age in the validation dataset (Figure 1).

**FIGURE 2:**
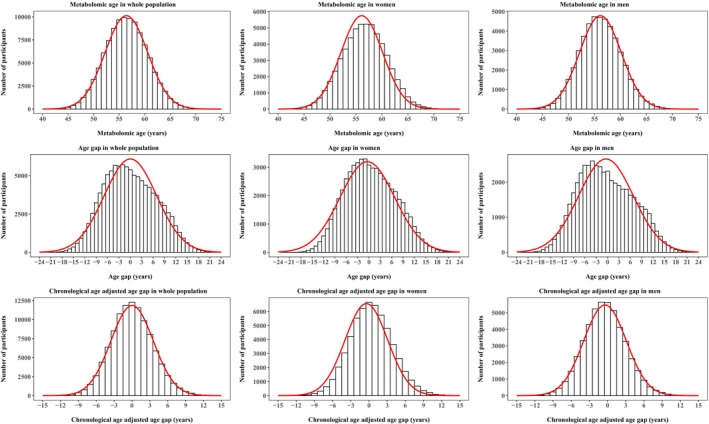
Distribution of metabolomic age in women and men. Age gap was calculated by subtracting chronological age from metabolomic age. Chronological age‐adjusted age gap was calculated with use of regression models. Red curves represent the trend of the distribution.

The distribution of predicted metabolomic age is shown in Figure 2. The correlation between metabolomic age and chronological age was 0.56 (95% CI: 0.55–0.56). The mean gap between metabolomic age and chronological age was 0.02 (SD = 6.93) years and the mean absolute error was 5.52 (SD = 4.27) years.

The age gap decreased with increasing age and similar trends were seen in both women and men. The age gap was 5.1 ± 5.1 years in individuals aged younger than 60 years and −5.0 ± 4.4 years in those aged 60 years or older (Figure S1). Therefore, CAAG was used in the association analysis.

Top 10 leading determinants of metabolomic age included omega‐3 fatty acids, docosahexaenoic acid, citrate, triglycerides in intermediate‐density lipoprotein, triglycerides in large low‐density lipoprotein, triglycerides in very small very low‐density lipoprotein, triglycerides in large high‐density lipoprotein, triglycerides in low‐density lipoprotein, tyrosine and saturated fatty acids (Figure S2). Similar metabolomic profiles that were the strongest predictors of chronological age were found (Figure S3).

### 3.3 Baseline characteristics

Individuals with greater CAAG were more likely to be female and have lower household income and higher BMI and a higher prevalence of hypertension (Table 1).

**TABLE 1:** Baseline characteristics across quintiles of chronological age‐adjusted age gap.

	Chronological age‐adjusted age gap (years)^a^					
	Quintile 1	Quintile 2	Quintile 3	Quintile 4	Quintile 5	*p*‐value^b^
	(≤2.90)	(−2.90, −0.92)	(−0.93, 0.80)	(0.81, 2.9)	(>2.9)	
Age (years)	56.6 ± 8.2	56.5 ± 8.2	56.5 ± 8.1	56.5 ± 8.1	56.6 ± 8.0	1.00
Sex						
Women	4994 (45)	5645 (51)	5959 (54)	6373 (58)	6954 (63)	<0.0001
Men	6076 (55)	5423 (49)	5110 (46)	4696 (42)	4116 (37)	
Ethnicity						
White	10,320 (93)	10,410 (94)	10,519 (95)	10,526 (95)	10,452 (94)	<0.0001
Non‐White	750 (7)	658 (6)	550 (5)	543 (5)	618 (6)	
Education						
High	3916 (35)	3787 (34)	3671 (33)	3692 (33)	3589 (32)	0.00011
Intermediate	5401 (49)	5452 (49)	5623 (51)	5556 (50)	5581 (51)	
Low	1753 (16)	1829 (17)	1775 (16)	1821 (17)	1900 (17)	
Household income (pounds)						
<18,000	2329 (21)	2326 (21)	2414 (22)	2449 (22)	2798 (25)	<0.0001
18,000‐30,999	3189 (29)	3164 (29)	3158 (28)	3197 (29)	3329 (30)	
31,000‐51,999	2932 (27)	2933 (26)	2855 (26)	2842 (26)	2667 (24)	
52,000‐100,000	2088 (19)	2124 (19)	2094 (19)	2067 (19)	1851 (17)	
>100,000	532 (5)	521 (5)	548 (5)	514 (5)	425 (4)	
Alcohol consumption						
Never	482 (5)	477 (4)	458 (4)	474 (4)	529 (5)	0.0323
Previous	374 (3)	418 (4)	366 (3)	349 (3)	460 (4)	
Current	10,214 (92)	10,173 (92)	10,245 (93)	10,246 (93)	10,081 (91)	
Smoking						
Never	6115 (55)	6058 (55)	6158 (56)	6007 (54)	5926 (53)	0.0277
Former	3777 (34)	3852 (35)	3806 (34)	3908 (35)	3973 (36)	
Current	1178 (11)	1158 (10)	1105 (10)	1154 (11)	1171 (11)	
Physical activity (MET‐minutes/week)	2743 ± 2808	2679 ± 2734	2623 ± 2626	2588 ± 2611	2512 ± 2653	<0.0001
Diet score^c^						
Unhealthy diet	4465 (40)	4341 (39)	4202 (38)	4035 (36)	3903 (35)	<0.0001
Healthy diet	6605 (60)	6727 (61)	6867 (62)	7034 (64)	7167 (65)	
Sleep duration (hours)	7.13 ± 1.07	7.14 ± 1.08	7.15 ± 1.10	7.18 ± 1.12	7.19 ± 1.20	<0.0001
BMI (kg/m^2^)	26.47 ± 4.06	27.00 ± 4.27	27.45 ± 4.50	27.79 ± 4.91	28.64 ± 5.52	<0.0001
Genetic risk score for longevity^d^	0.49 ± 0.05	0.49 ± 0.05	0.49 ± 0.05	0.49 ± 0.05	0.49 ± 0.05	0.0195
Fasting duration (hours)	3.80 ± 2.50	3.79 ± 2.47	3.74 ± 2.31	3.75 ± 2.40	3.84 ± 2.45	0.48
Hypertension	2434 (22)	2607 (24)	2863 (26)	3158 (29)	3738 (34)	<0.0001
High cholesterol	1805 (16)	2022 (18)	2361 (21)	2559 (23)	3206 (29)	<0.0001
Diabetes	322 (3)	378 (3)	445 (4)	599 (5)	1130 (10)	<0.0001
Antihypertensive medication	1809 (16)	1969 (18)	2168 (20)	2486 (23)	3145 (28)	<0.0001
Lipid lowering medication	1286 (12)	1546 (14)	1807 (16)	2142 (19)	2962 (27)	<0.0001
Glucose lowering medication	210 (2)	269 (2)	315 (3)	456 (4)	873 (8)	<0.0001

*Note*: Data are means ± standard deviations, or *N* (%).

Abbreviations: BMI, body mass index; MET, metabolic equivalent.

^a^ Age gap was calculated by subtracting chronological age from metabolomic age. Chronological age‐adjusted age gap was calculated with use of regression models.

^b^ ANOVA for continuous variables and chi‐squared for categorical variables were used to test the difference of baseline characteristics across quintiles of chronological age‐adjusted age gap.

^c^ Diet score was computed based on seven commonly eaten food groups following recommendations on dietary priorities for cardiometabolic health with a higher score representing a healthier diet.

^d^ Genetic risk score was calculated for longevity was calculated using 78 single‐nucleotide polymorphisms.

### 3.4 Chronological age‐adjusted age gap and incidence of individual diseases

Given the difference in the number of cases at baseline between individual diseases, the follow‐duration differed between these diseases. The median follow‐up duration ranged from 11.2 years for dyspepsia to 11.9 years for multiple sclerosis. The number of incident cases ranged from 46 for multiple sclerosis to 6484 for dyspepsia.

After controlling for false discovery rate, CAAG was significantly associated with the incidence of 25 out of 50 individual chronic diseases in Model 1. After adjustment for demographic factors, lifestyle, fasting duration, metabolic disorders and mediations use for hypertension and high cholesterol, the association with 15 chronic diseases remained significant. Each year increment of CAAG was associated with a 1% (95% CI: 0%–2%), 3% (1%–4%), 2% (1%–4%), 3% (1%–5%), 2% (0.3%–4%) and 2% (1%–3%) higher risk of CHD, heart failure, atrial fibrillation, stroke and other cardiac disease, respectively. Greater GAAG was associated with an increased risk of diabetes (HR (95% CI): 1.05 [1.03–1.06]), hypertension (1.01 [1.00–1.02]), dyslipidemia (1.02 [1.01–1.03]), oesophageal cancer (1.06 [1.02–1.11]), other cancers (1.01 [1.00–1.02]), alcohol use disorder (1.04 [1.01–1.06]), chronic liver disease (1.07 [1.03–1.11]), COPD (1.02 [1.01–1.03]), CKD (1.05 [1.04–1.06]) and age related macular degeneration (1.02 [1.00–1.04]).

**FIGURE 3:**
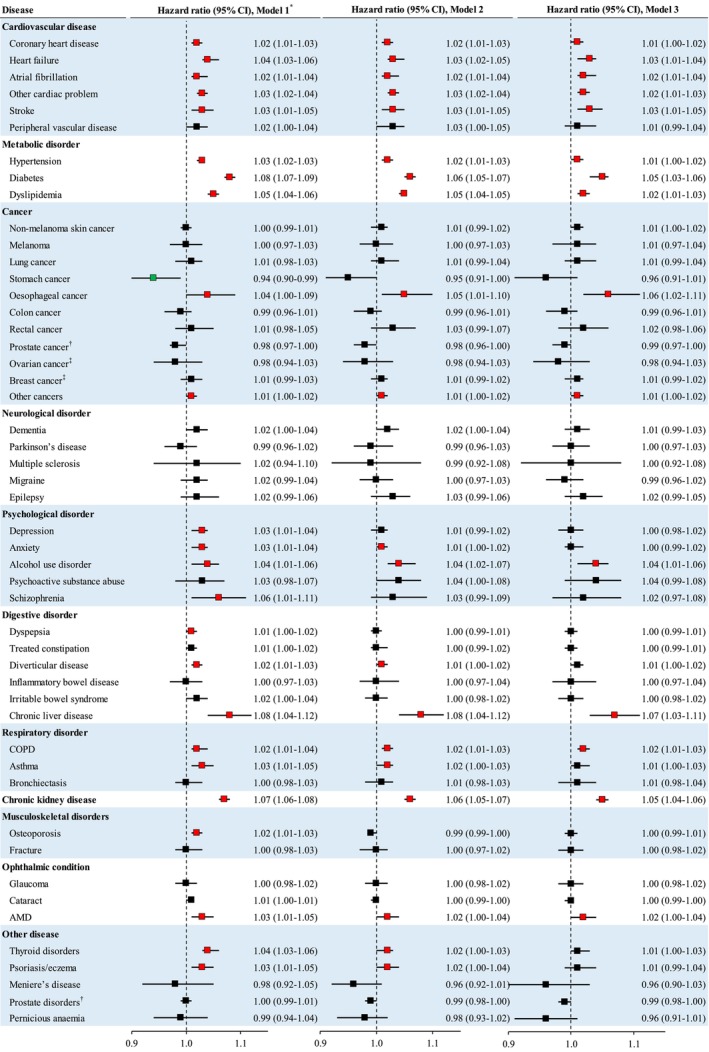
The association between each year increment in chronological age‐adjusted age gap and risk of individual diseases in the validation population. Age gap was calculated by subtracting chronological age from metabolomic age. Chronological age‐adjusted age gap was calculated with the use of regression models. *Cox proportional regression models were used to examine the association between chronological age‐adjusted age gap (each year increment) and incidence of individual chronic diseases. Model 1 was unadjusted; Model 2 was adjusted for Model 1 plus age, sex, ethnicity, education, household income, diet score, alcohol consumption, physical activity, smoking, sleep duration, fasting duration, and GRS for longevity; Model 3 was adjusted for Model 2 plus BMI, high cholesterol, hypertension, and antihypertensive and lipid‐lowering medications (hypertension or antihypertensive medication use at baseline was not adjusted for the analysis of incident hypertension given these participants with hypertension or antihypertensive medication use were excluded from the analysis). Red color squares refer to significantly positive associations while green color squares refer to significantly inverse associations. The significant associations in Model 1 were defined as *p*‐value<0.05 after adjustment for false discovery rate. ^†^These analyses were conducted among men only. ^‡^These analyses were conducted among women only. AMD, age related macular degeneration; CI, confidence interval; COPD, chronic obstructive pulmonary disease; HR, hazard ratio.

The association between CAAG and risk of anxiety, asthma, diverticulitis, thyroid disorders and eczema was attenuated to be non‐significant after adjustment for metabolic disorders and the use of related medications (Figure 3).

This was inconsistent with the results when CAAG was analysed in quintiles as categorical variables (Table S3). The survival plots for multiple individual diseases by quintiles of CAAG with significant associations are shown in Figure S4.

### 3.5 Moderation analysis

**FIGURE 4:**
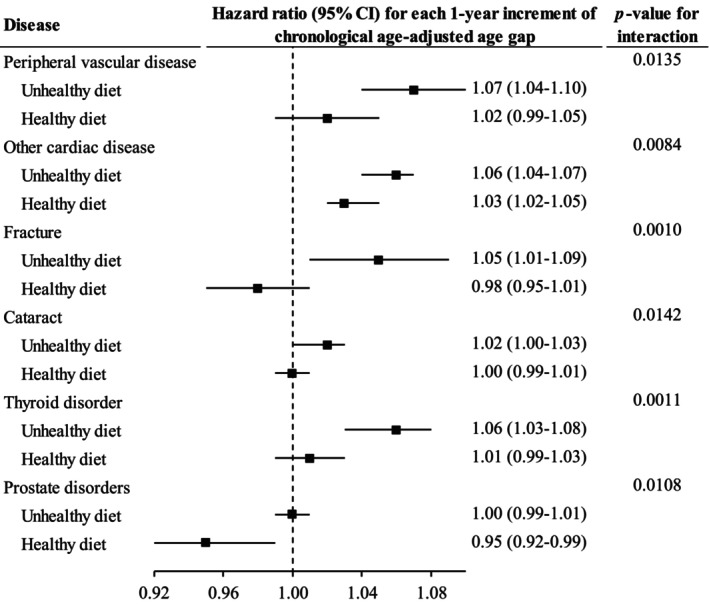
The association between chronological age‐adjusted age gap and incidence of chronic diseases moderated by diet score. Cox proportional regression models were used to test whether diet quality modified the association between chronological age‐adjusted age gap and incidence of chronic diseases. Only the results with significant interaction are shown in this figure. Horizontal lines indicate the range of the 95% confidence interval. The vertical dash lines represent the hazard ratio of one.

The association between CAAG and risk of peripheral vascular disease, other cardiac diseases, fracture, cataract and thyroid disorder was stronger among individuals with unhealthy diet. Greater CAAG was associated with a lower risk of prostate disorders in individuals with healthy diet (HR [95% CI] for each year increment: 0.95 [0.92–0.99]) but not those with unhealthy diet (1.00 [0.99–1.01], Figure 4).

**FIGURE 5:**
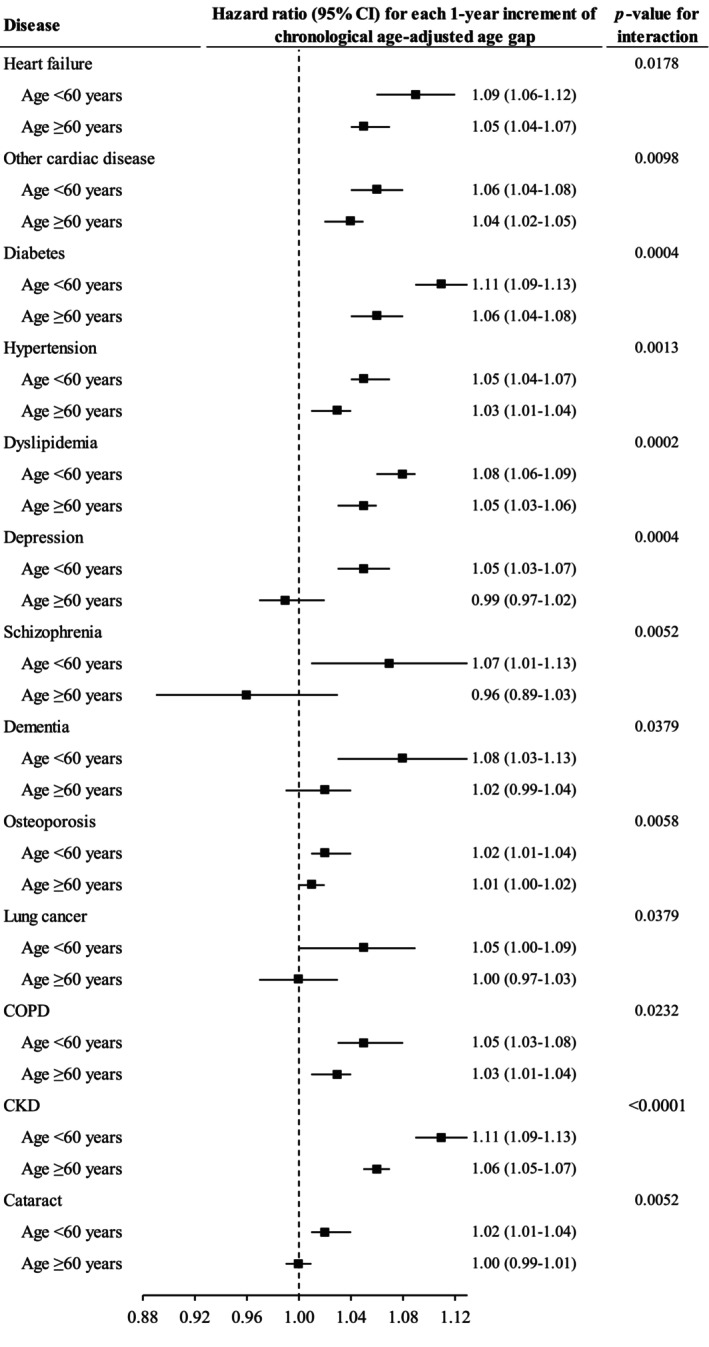
The association between chronological age‐adjusted age gap and incidence of chronic diseases moderated by chronological age. Cox proportional regression models were used to test whether chronological age modified the association between chronological age‐adjusted age gap and incidence of chronic diseases. Only the results with significant interaction are shown in this figure. Horizontal lines indicate the range of the 95% confidence interval. The vertical dash lines represent the hazard ratio of one. CKD, chronic kidney disease; CI, confidence interval; COPD, chronic obstructive pulmonary disease.

The association between CAAG and risk of heart failure, other cardiac diseases, diabetes, hypertension, dyslipidemia, COPD and CKD was stronger among younger than in older individuals. Greater CAAG was associated with increased risk of depression, schizophrenia, dementia, osteoporosis, lung cancer and cataract in younger individuals only (Figure 5).

**FIGURE 6:**
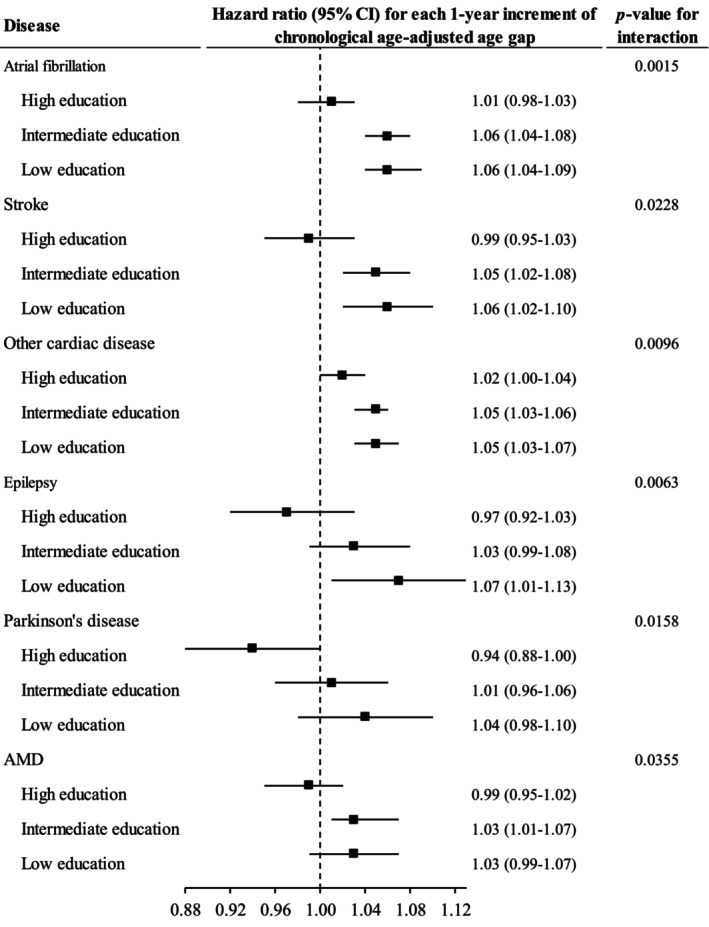
The association between chronological age‐adjusted age gap and incidence of chronic diseases moderated by education. Cox proportional regression models were used to test whether education modified the association between chronological age‐adjusted age gap and incidence of chronic diseases. Only the results with significant interaction are shown in this figure. Horizontal lines indicate the range of the 95% confidence interval. The vertical dash lines represent the hazard ratio of one. AMD, age‐related macular degeneration; CI, confidence interval.

The association between CAAG and risk of some CVDs, some neurological disorders, and age related macular degeneration was stronger in individuals with lower education (Figure 6).

The association between CAAG and risk of melanoma and chronic liver disease was stronger among individuals without hypertension than those with hypertension. Larger CAAG was associated with an increased risk of peripheral vascular disease, inflammatory bowel disease and cataract was significant among those with diabetes only. The association between CAAG and risk of epilepsy, hypertension and dyslipidemia was stronger in individuals with low GRS for longevity than those with high GRS (Figure S5).

### 3.6 Sensitivity analysis

Similar results for the association between chronological age‐adjusted age gap and risk of individual diseases were seen among individuals by excluding those developed the disease in the first year of follow‐up (Figure S6, Table S4) or by excluding those developed in the first 5 years of follow‐up (Figure S7, Table S5).

## 4 DISCUSSION

Using data from this large cohort study, we found greater CAAG was associated with an increased risk of 24 individual chronic diseases. The association with 15 diseases including most CVDs, all metabolic disorders, some cancers, alcohol use disorder, CKD, respiratory disorders, chronic liver disease and age related macular degeneration remained significant after adjustment for metabolic disorders and the use of related medications at baseline. The association with some CVDs, fracture, thyroid disorders and cataract was stronger among individuals with unhealthy diet. Greater CAAG was associated with lower risk of prostate disorders among individuals with healthy diet only. The association between CAAG and some chronic diseases was stronger among younger individuals, lowly educated individuals or those with metabolic disorders/low GRS of longevity.

The strong associations between metabolism and ageing provide the rationale for examining metabolomic clocks (López‐Otín et al., 2016). Metabolomic profiles including fatty acids, lipids and amino acids have been demonstrated to be strong predictors of longevity (Gonzalez‐Covarrubias et al., 2013). Consistently, we found fatty acids, amino acids and triglycerides (in intermediate‐density lipoprotein, large low‐density lipoprotein, very small very low‐density lipoprotein, large high‐density lipoprotein and low‐density lipoprotein) were among leading determinants of metabolomic age. Recent evidence suggests machine learning based on a larger training sample size has improved the precision of epigenetic clock estimates (Zhang et al., 2019). Our study showed that machine learning based LASSO and ridge regression analysis provided fair prediction of chronological age based on metabolomics. Noise in metabolomic data may limit the utility of metabolomic age (Rutledge et al., 2022), such that previous studies on metabolomic age only focused on several conditions including mortality, CVDs, obesity, diabetes and psychological disorders (Deelen et al., 2019; Fischer et al., 2014; Hertel et al., 2016; Menni et al., 2013; Robinson et al., 2020). Whether metabolomic age is associated with the risk of a wide range of chronic diseases needs to be explored in more cohort studies.

The important role of metabolomic profiles on CMDs has been highlighted in previous studies (Buergel et al., 2022; Shah et al., 2012). We found accelerated CAAG was associated with an increased risk of most CMDs of interest including CHD, heart failure, stroke and diabetes. Although these associations were independent of metabolic disorders and the use of medications for hypertension and lipids at baseline, the adjustment for these covariates attenuated the associations. This suggests good management of metabolic disorders may help mitigate the adverse effect size of accelerated metabolomic age on CMDs. It is also possible that the intake of respective medications might reduce the validity of the metabolomics thus biasing the algorithm of metabolomic age. In a cross‐sectional analysis of data from 26 community and hospital‐based cohorts, larger age gap (metabolomic age minus chronological age) was associated with a higher likelihood of diabetes (OR [95% CI] for each 10‐year increment of age gap: 2.52 [1.93–3.11]) among 12,633 participants (van den Akker et al., 2020). In the same study, a longitudinal analysis of 5410 participants with a mean follow‐up of 3.3 years showed that larger age gap was associated with an increased risk of CHD (HR [95% CI] for each 10‐year increment: 1.25 [1.11–1.40]) (van den Akker et al., 2020). Data from the UK Airwave cohort demonstrated that metabolomic age was corrected with obesity and diabetes (Robinson et al., 2020). Our findings regarding heart failure, atrial fibrillation, stroke, peripheral vascular disease and other cardiac disease need to be confirmed by future cohort studies with long follow‐up duration.

As metabolomic state has been linked to multiple diseases (Buergel et al., 2022), it is also of great interest to examine whether metabolomic age is predictive of many other diseases rather than CMDs only. Robinson et al. reported that metabolomic age acceleration was associated with heavy alcohol use and depression (Robinson et al., 2020). Likely, we found greater CAAG was associated with higher risk of incident depression, anxiety, alcohol use disorder and psychoactive substance abuse. The association for depression and anxiety was attenuated to be non‐significant after adjustment for metabolic disorders and antihypertensive and lipid‐lowering medications indicating that good control of traditional metabolic biomarkers might contribute to the reduction of the risk due to metabolomic age acceleration. A cross‐sectional analysis of data from the Study of Health in Pomerania showed that metabolic age acceleration was associated with kidney malfunction (microalbuminuria, albumin‐to‐creatinine ratio) (Hertel et al., 2016). A recent longitudinal multi‐omics study in humans revealed that kidney dysfunction was involved in the process of ageing (Ahadi et al., 2020). This is consistent with our study demonstrating that CAAG was associated with an increased risk of CKD. In a cohort study of 6055 individuals from the UK, an age‐related metabolite C‐glycosyl tryptophan was associated with lung function (forced expiratory volume) (Menni et al., 2013). We found greater CAAG was associated with an increased risk of COPD and asthma and the association for COPD was even independent of metabolic disorders and related medications use. The longitudinal multi‐omics study also identified an ageing pathway related to liver dysfunction (Ahadi et al., 2020). Consistently, metabolomic age was strongly associated with the risk of liver disease in our study. We also found larger CAAG was associated with a higher risk of oesophageal cancer but not other cancers. A recent systematic review reported that a number of metabolites were identified for oesophageal cancer but the results were inconsistent between previous studies (Huang et al., 2020). Meanwhile, metabolomic age acceleration was associated with an increased risk of dyspepsia, diverticulitis, osteoporosis and thyroid disorders before but not after adjustment for metabolic biomarkers and use of antihypertensive and lipid‐lowering medications in our study. Our study developed a metabolomic age that was independently predictive of multiple chronic diseases including psychological disorders, CKD, COPD, chronic liver disease and oesophageal cancer, which might be useful for the screening and prevention of these diseases.

The importance of diet in the development of chronic diseases should not be overlooked (Schulze et al., 2018; Shan et al., 2020). In moderation analysis, the association between CAAG and the risk of several types of CVD, CKD and chronic liver disease was weaker among individuals with healthy diet. Therefore, individuals are recommended to accommodate healthy diet habits to mitigate the risk of metabolomic ageing. Stronger associations between metabolomic age and some chronic diseases seen in individuals with metabolic disorders in our study highlight the importance of the management of metabolomics in these people. The association between CAAG and the risk of some chronic diseases was more pronounced among young than older individuals. This is consistent with previous studies showing that metabolic disorders diagnosed at younger age was associated with greater risk of CVD, dementia and mortality (Shang et al., 2021, 2022; Zhao et al., 2021). The association between CAAG and the risk of some chronic diseases was stronger among individuals with lower education. This is possibly due to the fact that individuals with higher education are more likely to seek health care and less likely to develop chronic diseases with metabolomic ageing (Brayne et al., 2010; Livingston et al., 2020). We also found that greater CAAG was associated with an increased risk of epilepsy, hypertension and dyslipidemia in individuals with high GRS of longevity. One possible explanation for this is that low GRS of longevity was associated with higher prevalence of metabolic disorders and higher genetic risks of epilepsy, hypertension and dyslipidemia were detected in long‐lived individuals (Hu et al., 2022). Metabolomic age provides different prediction values of some chronic diseases between diet, education, metabolic disorders, age or GRS groups.

This is the first study with a large sample size and long follow‐up duration to develop metabolomic age and examine its association with a wide range of chronic diseases. Several potential limitations need to be considered in our study. First, the metabolomic age was developed based on metabolomics measured at one time point, such that metabonomic dynamics with ageing within an individual could not be estimated. Second, the plasma sample in the UK Biobank was non‐fasting, which might bias the associations. However, the adjustment for fasting duration in the analysis did not substantially change the association between CAAG and chronic diseases. Third, most of the participants in our analyses were Caucasians thus our findings may not be generalized to other ethnic groups. Finally, the number of incident cases for several chronic diseases (such as multiple sclerosis) was small, which might have reduced the statistical power to test significance.

In conclusion, metabolomic age plays an important role in the development of multiple chronic diseases including CMDs, psychological disorders, COPD, CKD, liver disease and some cancers. Healthy diet may help mitigate the risk for some chronic diseases due to metabolomic age acceleration. Age, education, metabolic disorders and GRS for longevity may modify the association between metabolomic age and some chronic diseases. Our findings may help facilitate the understanding of ageing process related to metabolomics thus enhancing healthy ageing.

## AUTHOR CONTRIBUTIONS

XS, MH, and HY conceived and designed the study. ZZ, WW performed data curation. XS conducted data analysis and drafted the initial manuscript. XS, JL, XLZ, YH, SL, ZZ, WW, XYZ, ST, YH, ZG, HY, and MH made a critical revision to the manuscript for important intellectual content. All authors read the manuscript and approved the final draft.

## FUNDING INFORMATION

The study was in part supported by XLZ receives GDPH Supporting Fund for Talent Program (KJ2020633). ZZ receives support from the National Natural Science Foundation of China (82101173), the Research Foundation of Medical Science and Technology of Guangdong Province (B2021237). HY receives support from the National Natural Science Foundation of China (81870663, 82171075), the Outstanding Young Talent Trainee Program of Guangdong Provincial People's Hospital (KJ012019087), Guangdong Provincial People's Hospital Scientific Research Funds for Leading Medical Talents and Distinguished Young Scholars in Guangdong Province (KJ012019457), Talent Introduction Fund of Guangdong Provincial People's Hospital (Y012018145). MH receives support from the High‐level Talent Flexible Introduction Fund of Guangdong Provincial People's Hospital (No. KJ012019530). MH also receives support from the University of Melbourne at Research Accelerator Program and the CERA Foundation. The Centre for Eye Research Australia receives Operational Infrastructure Support from the Victorian State Government. The sponsor or funding organization had no role in the design or conduct of this research. The sponsor or funding organization had no role in the design, conduct, analysis, or reporting of this study. The funding sources did not participate in the design and conduct of the study; collection, management, analysis, and interpretation of the data; preparation, review, or approval of the manuscript; and decision to submit the manuscript for publication.

## CONFLICT OF INTEREST STATEMENT

The authors declare that they have no competing interests.

## Supporting information


Data S1.

## Data Availability

Data are available in a public, open access repository (https://www.ukbiobank.ac.uk/).
