# Supplementary material for: Metabolomic age and risk of 50 chronic diseases in community‐dwelling adults: A prospective cohort study
Source: Aging Cell. 2024 Feb 21;23(5):e14125. doi: 10.1111/acel.14125 (PMC11113347; doi:10.1111/acel.14125)
Supplement: Supplementary file 1 — Data S1. [file ACEL-23-e14125-s001.docx]

Figure S1. Age gap and chronological age

Figure S2. Leading metabolomic profiles for metabolomic age in women and men

Figure S3. Leading metabolomic profiles for chronological age in women and men

Figure S4. Incidence of individual chronic diseases by quintiles of chronological age-adjusted age gap

Figure S5. The association between chronological age-adjusted age gap and incidence of chronic diseases moderated by metabolic disorders and genetic risk score for longevity

Figure S6. The association between each year increment in chronological age-adjusted age gap and risk of individual diseases in the validation population with follow-up duration of ≥1 years

Figure S7. The association between each year increment in chronological age-adjusted age gap and risk of individual diseases in the validation population with follow-up duration of ≥5 years

Table S1. Field codes for diseases of interest

Table S2. ICD codes for diseases of interest

Table S3. The association between chronological age-adjusted age gap and risk of individual diseases in the validation population

Table S4. The association between chronological age-adjusted age gap and risk of individual diseases the validation population with follow-up duration of ≥1 years

Table S5. The association between chronological age-adjusted age gap and risk of individual diseases in the validation population with follow-up duration of ≥5 years


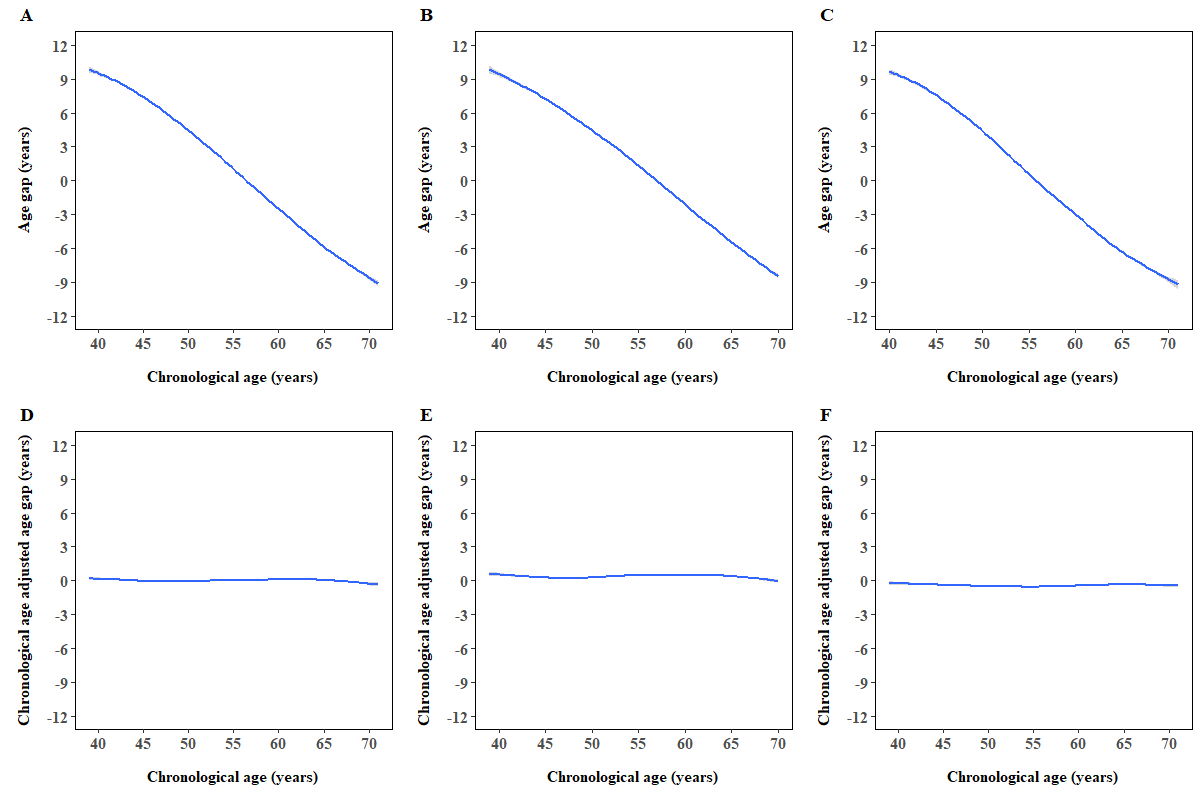


**Figure S1. Age gap and chronological age**

Pannels A, B, and C refer to the analysis for all participants, women, and men, respectively. Pannels D, E, and F refer to the analysis for all participants, women, and men, respectively. Age gap was calculated by subtracting chronological age from metabolomic age. Chronological age-adjusted age gap was calculated with use of regression models.


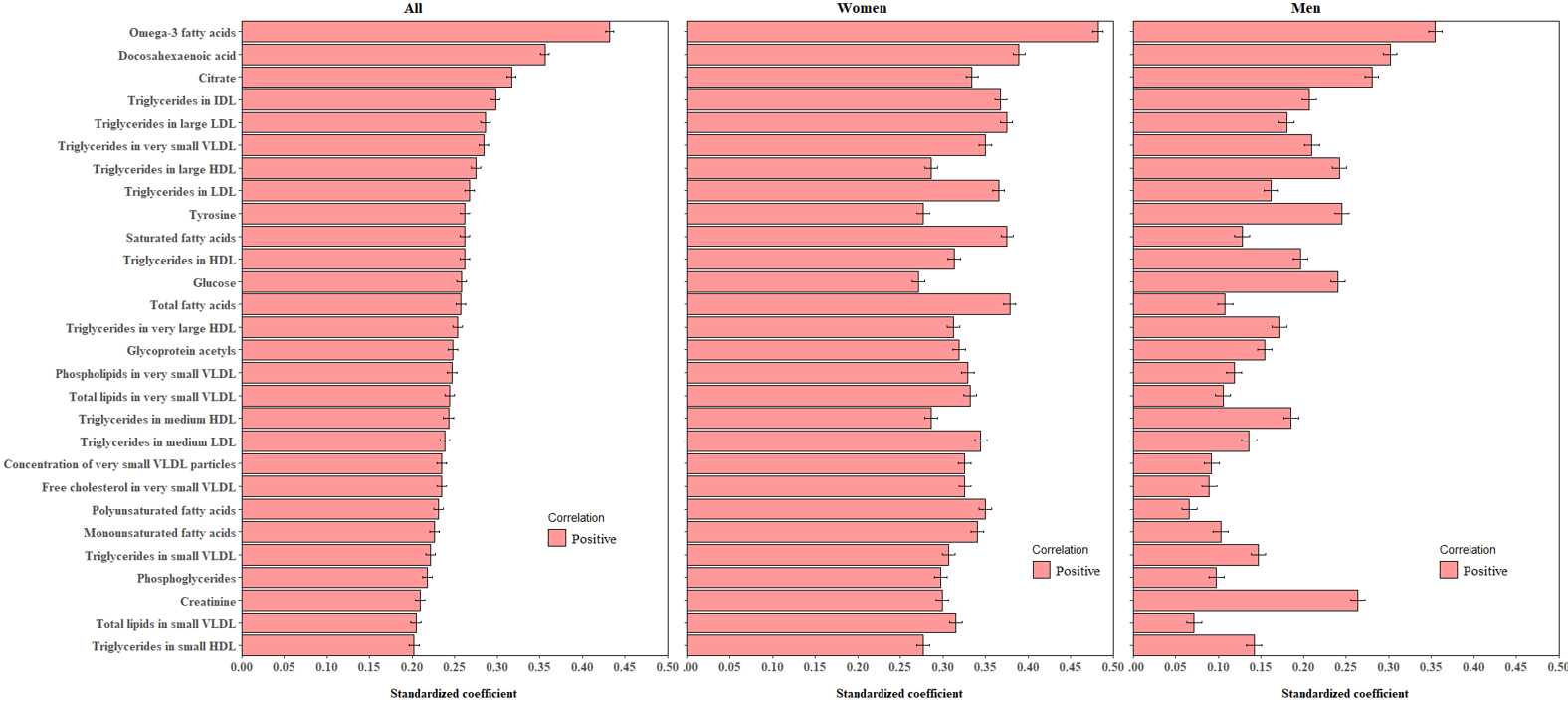


**Figure S2. Leading metabolomic profiles for metabolomic age in women and men**

Pearson's correlation coefficients between metabolomic age and 168 individual metabolomic profiles were analyzed. Metabolomic profiles with absolute coefficients ≥0.20 in the whole population are shown in this figure.


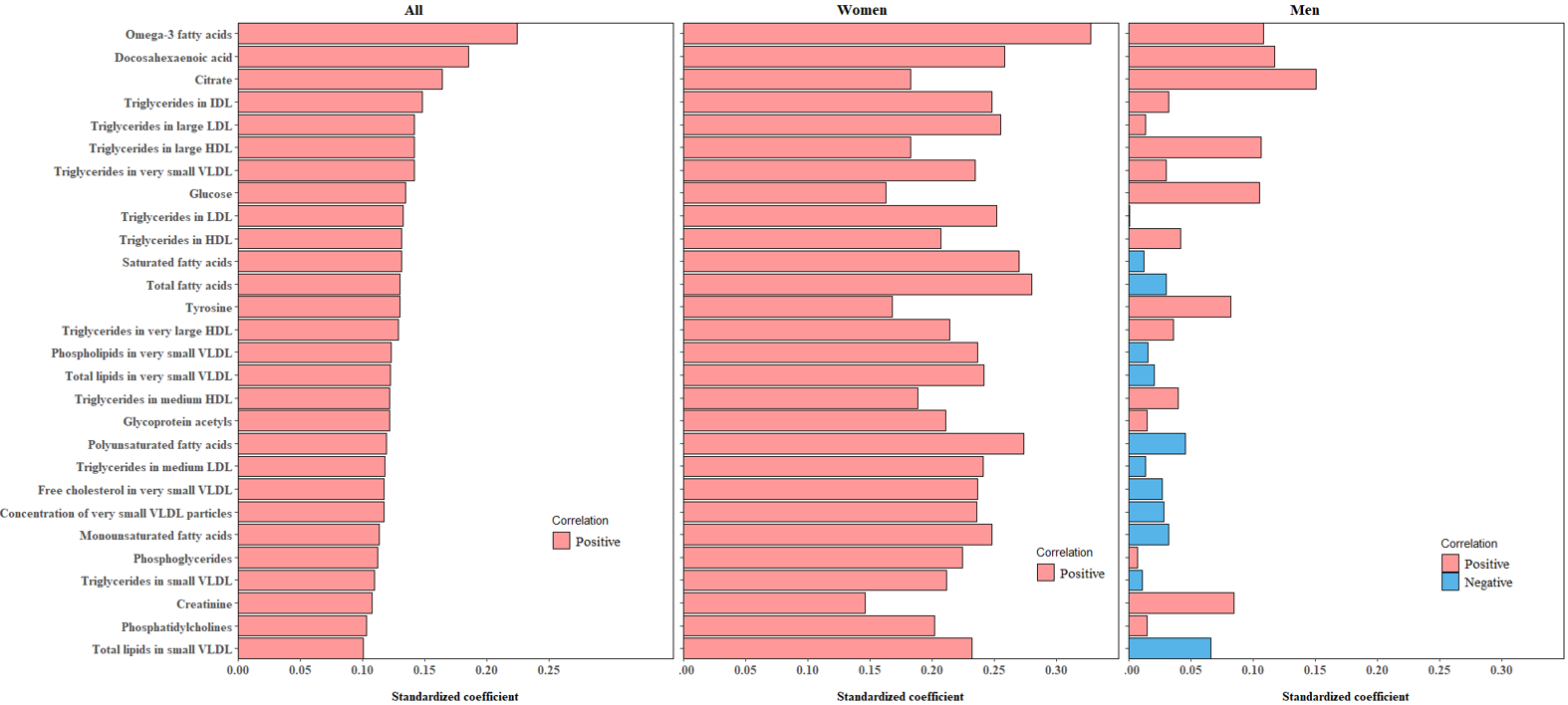


**Figure S3. Leading metabolomic profiles for chronological age in women and men**

Pearson's correlation coefficients between chronological age and 168 individual metabolomic profiles were analyzed. Metabolomic profiles with absolute coefficients ≥0.10 in the whole population are shown in this figure.


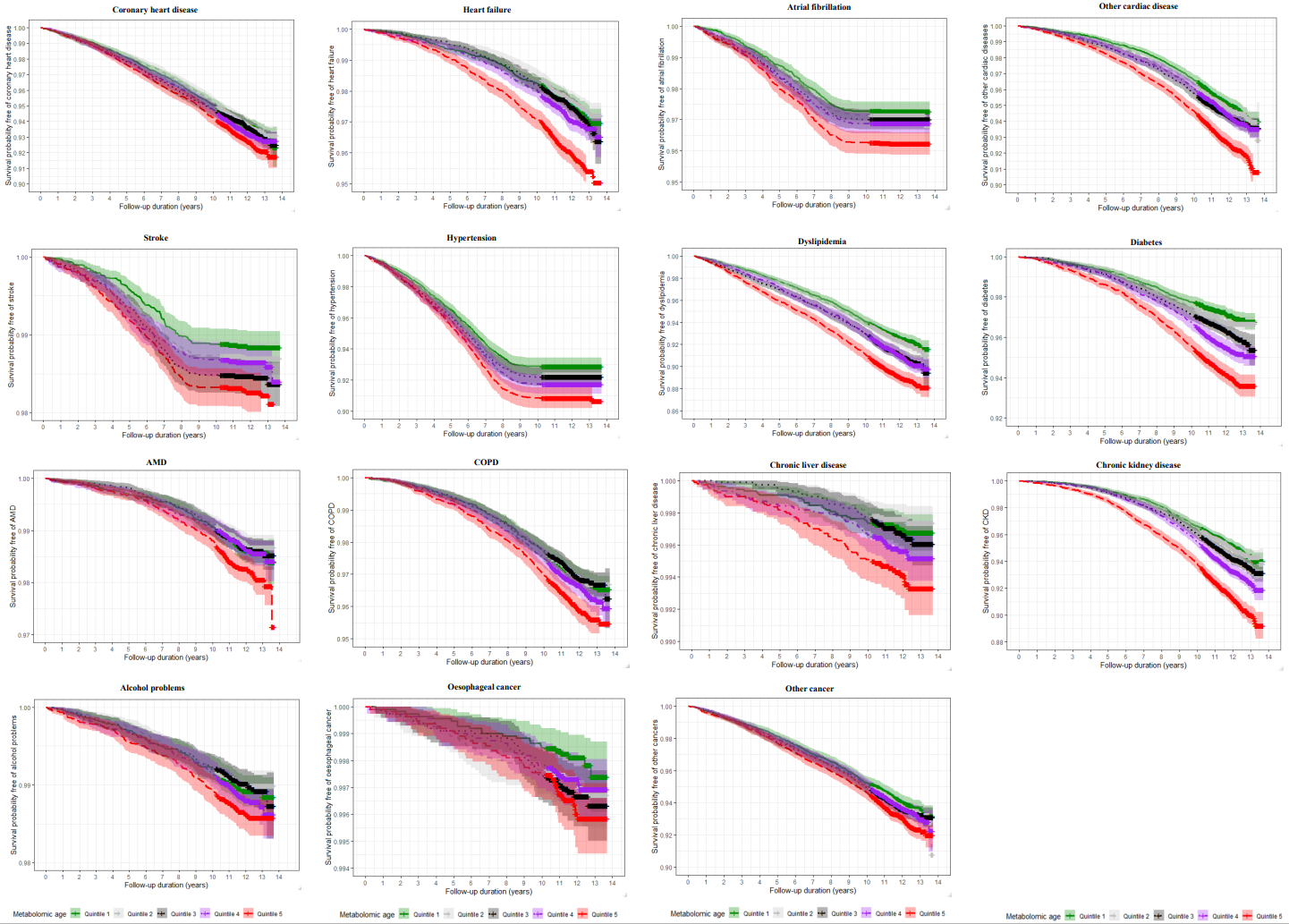


**Figure S4. Incidence of individual chronic diseases by quintiles of chronological age-adjusted age gap**

**Figure S5. The association between chronological age-adjusted age gap and incidence of chronic diseases moderated by metabolic disorders and genetic risk score for longevity**

AMD, age-related macular degeneration; CI, confidence interval.

Cox proportional regression models were used to test whether metabolic disorders or genetic risk score for longevity modified the association between chronological age-adjusted age gap and incidence of chronic diseases. Only the results with significant interaction are shown in this figure. Horizontal lines indicate the range of the 95% confidence interval. The vertical dash lines represent the hazard ratio of 1.

Figure S6. The association between each year increment in chronological age-adjusted age gap and risk of individual diseases in the validation population with follow-up duration of ≥1 years

AMD, age related macular degeneration; CI, confidence interval; COPD, chronic obstructive pulmonary disease; HR, hazard ratio.

Age gap was calculated by subtracting chronological age from metabolomic age. Chronological age-adjusted age gap was calculated with the use of regression models.

*Cox proportional regression models were used to examine the association between chronological age-adjusted age gap (each year increment) and incidence of individual chronic diseases. Model 1 was unadjusted; Model 2 was adjusted for Model 1 plus age, sex, ethnicity, education, household income, diet score, alcohol consumption, physical activity, smoking, sleep duration, fasting duration, and GRS for longevity; Model 3 was adjusted for Model 2 plus BMI, high cholesterol, hypertension, and antihypertensive and lipid-lowering medications (hypertension or antihypertensive medication use at baseline was not adjusted for the analysis of incident hypertension given these participants with hypertension or antihypertensive medication use were excluded from the analysis). Red color squares refer to significantly positive associations. The significant associations in Model 1 were defined as P-value<0.05 after adjustment for false discovery rate.

^†^These analyses were conducted among men only.

^‡^These analyses were conducted among women only.

Figure S7. The association between each year increment in chronological age-adjusted age gap and risk of individual diseases in the validation population with follow-up duration of ≥5 years

AMD, age related macular degeneration; CI, confidence interval; COPD, chronic obstructive pulmonary disease; HR, hazard ratio.

Age gap was calculated by subtracting chronological age from metabolomic age. Chronological age-adjusted age gap was calculated with the use of regression models.

*Cox proportional regression models were used to examine the association between chronological age-adjusted age gap (each year increment) and incidence of individual chronic diseases. Model 1 was unadjusted; Model 2 was adjusted for Model 1 plus age, sex, ethnicity, education, household income, diet score, alcohol consumption, physical activity, smoking, sleep duration, fasting duration, and GRS for longevity; Model 3 was adjusted for Model 2 plus BMI, high cholesterol, hypertension, and antihypertensive and lipid-lowering medications (hypertension or antihypertensive medication use at baseline was not adjusted for the analysis of incident hypertension given these participants with hypertension or antihypertensive medication use were excluded from the analysis). Red color squares refer to significantly positive associations. The significant associations in Model 1 were defined as P-value<0.05 after adjustment for false discovery rate.

^†^These analyses were conducted among men only.

^‡^These analyses were conducted among women only.

**Table S1. Field codes for diseases of interest**

| **Long term condition grouping** | **Conditions included as reported by participants** | **Field Code** |
| --- | --- | --- |
| 1. Hypertension | Hypertension  Essential Hypertension | 1065  1072 |
| 2. Depression | Essential Depression | 1072 |
|  | Postnatal Depression | 1531 |
| 3. Asthma | Asthma | 1111 |
| 4. Atrial fibrillation | Atrial Fibrillation | 1471 |
| 5. Coronary heart disease | Heart attack/Myocardial Infarction | 1075 |
|  | Angina | 1074 |
| 6. Dyspepsia | Gastro-oesophageal reflux (GORD)/gastric reflux | 1138 |
|  | Oesophagitis /Barrett's oesophagus | 1139 |
|  | Gastric stomach ulcers | 1142 |
|  | Gastric erosions/gastritis | 1143 |
|  | Duodenal ulcer | 1457 |
|  | Dyspepsia/indigestion | 1510 |
|  | Hiatus hernia | 1474 |
|  | Helicobacter pylori | 1442 |
| 7. Diabetes | Diabetic nephropathy | 1607 |
|  | Diabetic neuropathy/ulcers | 1468 |
|  | Diabetes | 1220 |
|  | Type 1 diabetes | 1222 |
|  | Type 2 diabetes | 1223 |
|  | Diabetic eye disease | 1276 |
|  | Thyroid problem (not cancer) | 1224 |
| 8. Thyroid disorders | Hyperthyroidism/thyrotoxicosis | 1225 |
|  | Hypothyroidism/myxoedema | 1226 |
|  | Grave’s disease | 1522 |
|  | Thyroid goitre | 1610 |
|  | Thyroiditis | 1428 |
| 9. Chronic Obstructive Pulmonary Disease (COPD) | COPD/chronic obstructive airways disease | 1112 |
|  | Emphysema/chronic bronchitis | 1113 |
|  | Emphysema | 1472 |
| 10. Anxiety | Anxiety/panic attacks | 1287 |
|  | Nervous breakdown | 1288 |
|  | Post-traumatic stress disorder | 1469 |
|  | Obsessive compulsive disorder | 1615 |
|  | Stress | 1614 |
|  | Insomnia | 1616 |
|  | Psychological/psychiatric problem | 1243 |
| 11. Irritable bowel syndrome | Irritable bowel syndrome | 1154 |
| 12. Alcohol use disorder | Alcohol dependency | 1408 |
|  | Alcoholic liver disease/alcoholic cirrhosis | 1604 |
| 13. Other psychoactive substance abuse | Opioid dependency | 1409 |
|  | Other substance abuse/dependency | 1410 |
| 14. Treated constipation | Constipation | 1599 |
| 15. Stroke/Transient Ischaemic Attack (TIA) | Stroke | 1081 |
|  | TIA | 1082 |
|  | Subarachnoid haemorrhage | 1083 |
|  | Brain haemorrhage | 1086 |
|  | Ischaemic stroke | 1583 |
| 16. Chronic kidney disease | Polycystic kidney | 1427 |
|  | Diabetic nephropathy | 1607 |
|  | Renal/kidney failure | 1192 |
|  | Renal failure requiring dialysis | 1193 |
|  | Renal failure not requiring dialysis | 1194 |
|  | Kidney nephropathy | 1519 |
|  | Immunoglobulin A (IgA) nephropathy | 1520 |
| 17. Diverticular disease | Diverticular disease | 1458 |
|  | Diverticulitis | 1458 |
| 18. Peripheral vascular disease | Peripheral vascular disease | 1067 |
|  | Leg claudication/intermittent claudication | 1087 |
| 19. Heart failure | Cardiomyopathy | 1079 |
|  | Hypertrophic cardiomyopathy | 1588 |
|  | Heart failure/pulmonary oedema | 1076 |
| 20. Prostate disorders | Prostate problem (not cancer) | 1207 |
|  | Enlarged prostate | 1396 |
|  | Benign prostatic hypertrophy | 1516 |
| 21. Epilepsy | Epilepsy | 1264 |
| 22. Dementia | Dementia, Alzheimer’s disease, Cognitive impairment | 1263 |
| 23. Schizophrenia/bipolar disorder | Schizophrenia | 1289 |
|  | mania/bipolar disorder/manic depression | 1291 |
| 24. Psoriasis/eczema | Eczema/dermatitis | 1452 |
|  | Psoriasis | 1453 |
| 25. Inflammatory Bowel Disease | Inflammatory Bowel Disease | 1461 |
|  | Crohn’s disease | 1462 |
|  | Ulcerative colitis | 1463 |
| 26. Migraine | Migraine | 1265 |
| 27. Bronchiectasis | Bronchiectasis | 1114 |
| 28. Parkinson’s disease | Parkinson’s disease | 1262 |
| 29. Multiple Sclerosis | Multiple Sclerosis | 1261 |
| 30. Osteoporosis | Osteoporosis | 1465 |
| 31. Chronic liver disease | Oesophageal varices  Non infective hepatitis  Liver failure/cirrhosis  Primary biliary cirrhosis | 1141  1157  1158  1506 |
| 32. Meniere’s disease | Meniere’s disease | 1421 |
| 33. Pernicious Anaemia | Pernicious Anaemia | 1331 |
| 34. Heart/cardiac problem | Heart/cardiac problem | 1066 |
| 35. High cholesterol | High cholesterol | 1473 |
| 36. Fracture | fracture pelvis | 1647 |
|  | fracture neck of femur / hip | 1648 |
|  | fracture patella / knee | 1650 |
| 37. Glaucoma | Glaucoma | 1277 |
| 38. Cataract | Cataract | 1278 |
| 39. AMD | AMD | 1528 |
| 40. Lung Cancer | Lung Cancer | 1001 |
| 41. Skin Cancer | Non-melanoma Cancer | 1060 |
| 42. Melanoma | Melanoma | 1059 |
| 43. Stomach Cancer | Stomach cancer | 1018 |
| 44. Oesophageal cancer | Lung Cancer | 1017 |
| 45. Colon cancer | Colon cancer | 1022 |
| 46. Rectal cancer | Rectal_cancer | 1023 |
| 47. Prostate cancer | Prostate cancer | 1044 |
| 48. Ovarian cancer | Ovarian cancer | 1039 |
| 49. Breast cancer | Breast cancer | 1002 |
| 50. Other cancers | cancer of lip/mouth/pharynx/oral cavity | 1004 |
|  | salivary gland cancer | 1005 |
|  | larynx/throat cancer | 1006 |
|  | nasal cavity cancer | 1007 |
|  | ear cancer | 1008 |
|  | sinus cancer | 1009 |
|  | lip cancer | 1010 |
|  | tongue cancer | 1011 |
|  | gum cancer | 1012 |
|  | parotid gland cancer | 1015 |
|  | other salivary gland cancer | 1016 |
|  | small intestine/small bowel cancer | 1019 |
|  | large bowel cancer/colorectal cancer | 1020 |
|  | anal cancer | 1021 |
|  | liver/hepatocellular cancer | 1024 |
|  | gallbladder/bile duct cancer | 1025 |
|  | pancreas cancer | 1026 |
|  | small cell lung cancer | 1027 |
|  | non-small cell lung cancer | 1028 |
|  | peripheral nerve/autonomic nerve cancer | 1029 |
|  | eye and/or adnexal cancer | 1030 |
|  | meningeal cancer / malignant meningioma | 1031 |
|  | brain cancer / primary malignant brain tumour | 1032 |
|  | spinal cord or cranial nerve cancer | 1033 |
|  | kidney/renal cell cancer | 1034 |
|  | bladder cancer | 1035 |
|  | other cancer of urinary tract | 1036 |
|  | female genital tract cancer | 1037 |
|  | male genital tract cancer | 1038 |
|  | cervical cancer | 1041 |
|  | vaginal cancer | 1042 |
|  | vulval cancer | 1043 |
|  | testicular cancer | 1045 |
|  | penis cancer | 1046 |
|  | lymphoma | 1047 |
|  | leukaemia | 1048 |
|  | multiple myeloma | 1050 |
|  | myelofibrosis or myelodysplasia | 1051 |
|  | hodgkins lymphoma / hodgkins disease | 1052 |
|  | non-hodgkins lymphoma | 1053 |
|  | chronic lymphocytic | 1055 |
|  | chronic myeloid | 1056 |
|  | other haematological malignancy | 1058 |
|  | basal cell carcinoma | 1061 |
|  | squamous cell carcinoma | 1062 |
|  | primary bone cancer | 1063 |
|  | mesothelioma | 1064 |
|  | thyroid cancer | 1065 |
|  | parathyroid cancer | 1066 |
|  | adrenal cancer | 1067 |
|  | sarcoma/fibrosarcoma | 1068 |
|  | malignant lymph node, unspecified | 1070 |
|  | metastatic cancer (unknown primary) | 1071 |
|  | cin/pre-cancer cells cervix | 1072 |
|  | rodent ulcer | 1073 |
|  | acute myeloid leukaemia | 1074 |
|  | retinoblastoma | 1075 |
|  | kaposis sarcoma | 1076 |
|  | mouth cancer | 1077 |
|  | tonsil cancer | 1078 |
|  | oropharynx / oropharyngeal cancer | 1079 |
|  | trachea cancer | 1080 |
|  | thymus cancer / malignant thymoma | 1081 |
|  | heart / mediastinum cancer | 1082 |
|  | respiratory / intrathoracic cancer | 1084 |
|  | bone metastases / bony secondaries | 1085 |
|  | appendix cancer | 1086 |
|  | fallopian tube cancer | 1087 |
|  | malignant insulinoma | 1088 |

**Table S2. ICD codes for diseases of interest**

| **Long term condition grouping** | **Conditions included as reported by participants** | **ICD10 code** | **ICD9 code** |
| --- | --- | --- | --- |
| 1. Hypertension | Essential (primary) hypertension | I10 | 401 |
|  | Hypertensive heart disease | I11 | 402 |
|  | Hypertensive heart disease with (congestive) heart failure | I110 | 403 |
|  | Hypertensive heart disease without (congestive) heart failure | I119 | 404 |
|  | Hypertensive renal disease | I12 | 405 |
|  | Hypertensive renal disease with renal failure | I120 |  |
|  | Hypertensive renal disease without renal failure | I129 |  |
|  | Hypertensive heart and renal disease | I13 |  |
|  | Hypertensive heart and renal disease with (congestive) heart failure | I130 |  |
|  | Hypertensive heart and renal disease with renal failure | I131 |  |
|  | Hypertensive heart and renal disease with both (congestive) heart failure and renal failure | I132 |  |
|  | Hypertensive heart and renal disease, unspecified | I139 |  |
|  | Secondary hypertension | I15 |  |
|  | Renovascular hypertension | I150 |  |
|  | Hypertension secondary to other renal disorders | I151 |  |
|  | Hypertension secondary to endocrine disorders | I152 |  |
|  | Other secondary hypertension | I158 |  |
|  | Secondary hypertension, unspecified | I159 |  |
| 2. Depression | Depressive episode | F32 | 2962 |
|  | Recurrent depressive disorder | F33 | 2963 |
|  | Dysthymia | F341 | 3004 |
|  | Other recurrent mood [affective] disorders | F381 | 311 |
|  | Postschizophrenic depression | F204 |  |
| 3. Asthma | Asthma | J45 | 493 |
|  | Predominantly allergic asthma | J450 |  |
|  | Nonallergic asthma | J451 |  |
|  | Mixed asthma | J458 |  |
|  | Asthma, unspecified | J459 |  |
| 4. Atrial Fibrillation | Atrial fibrillation and flutter | I48 | 427 |
|  | Paroxysmal atrial fibrillation | I480 |  |
|  | Persistent atrial fibrillation | I481 |  |
|  | Chronic atrial fibrillation | I482 |  |
|  | Atrial fibrillation and atrial flutter, unspecified | I489 |  |
| 5. Coronary Heart Disease | Angina pectoris | I20 | 413 |
|  | Acute myocardial infarction | I21 | 410 |
|  | Subsequent myocardial infarction | I22 | 411 |
|  | Certain current complications following acute myocardial infarction | I23 | 412 |
|  | Other acute ischaemic heart diseases | I24 | 414 |
|  | Chronic ischaemic heart disease | I25 |  |
| 6. Dyspepsia | Gastro-oesophageal reflux disease | K21 | 53081 |
|  | Gastro-oesophageal reflux disease with oesophagitis | K210 | 5368 |
|  | Gastro-oesophageal reflux disease without oesophagitis | K219 |  |
|  | Oesophagitis | K20 |  |
|  | Barrett's oesophagus | K227 |  |
|  | Other specified diseases of oesophagus | K228 |  |
|  | Disease of oesophagus, unspecified | K229 |  |
|  | Disorders of oesophagus in diseases classified elsewhere | K23 |  |
|  | Gastric ulcer | K25 |  |
|  | Gastritis and duodenitis | K29 |  |
|  | Duodenal ulcer | K26 |  |
|  | Dyspepsia | K30 |  |
|  | Congenital hiatus hernia | Q401 |  |
|  | Helicobacter pylori [H.pylori] as the cause of diseases classified to other chapters | B980 |  |
| 7. Diabetes | Diabetic polyneuropathy | G632 | 250 |
|  | Diabetic mononeuropathy | G590 |  |
|  | Diabetic retinopathy | H360 |  |
|  | Diabetic cataract | H280 |  |
|  | Insulin-dependent diabetes mellitus | E10 |  |
|  | Non-insulin-dependent diabetes mellitus | E11 |  |
|  | Malnutrition-related diabetes mellitus | E12 |  |
|  | Other specified diabetes mellitus | E13 |  |
|  | Unspecified diabetes mellitus | E14 |  |
|  | Congenital iodine-deficiency syndrome | E00 | 240 |
| 8. Thyroid disorders | Thyrotoxicosis [hyperthyroidism] | E05 | 241 |
|  | Other hypothyroidism | E03 | 242 |
|  | Other non-toxic goitre | E04 | 243 |
|  | Iodine-deficiency-related thyroid disorders and allied conditions | E01 | 244 |
|  | Thyroiditis | E06 | 245 |
|  | Other disorders of thyroid | E07 | 246 |
|  | Subclinical iodine-deficiency hypothyroidism | E02 |  |
| 9. Connective tissue disorders | Myositis | M60 | 710 |
|  | Myopathy, unspecified | G729 |  |
|  | Systemic lupus erythematosus | M32 |  |
|  | Other benign neoplasms of connective and other soft tissue | D21 |  |
|  | Other systemic involvement of connective tissue | M35 |  |
|  | Connective tissue stenosis of neural canal | M994 |  |
|  | Connective tissue and disk stenosis of intervertebral foramina | M997 |  |
|  | Sicca syndrome [Sjogren] | M350 |  |
|  | Dermatopolymyositis | M33 |  |
|  | Localised scleroderma [morphea] | L940 |  |
|  | Linear scleroderma | L941 |  |
|  | Rheumatoid arthritis with involvement of other organs and systems | M053 |  |
|  | Other seropositive rheumatoid arthritis | M058 |  |
|  | Seropositive rheumatoid arthritis, unspecified | M059 |  |
|  | Other rheumatoid arthritis | M06 |  |
|  | Psoriatic and enteropathic arthropathies | M07 |  |
|  | Polymyalgia rheumatica | M353 |  |
|  | Postsurgical malabsorption osteoporosis | M813 |  |
|  | Coeliac disease | K900 |  |
| 10. Chronic obstructive pulmonary disease (COPD) | Simple and mucopurulent chronic bronchitis | J41 | 491 |
|  | Unspecified chronic bronchitis | J42 |  |
|  | Emphysema | J43 | 492 |
|  | Other chronic obstructive pulmonary disease | J44 | 494 |
| 11. Anxiety | Phobic anxiety disorders | F40 | 3000 |
|  | Other anxiety disorders | F41 | 3002 |
|  | Reaction to severe stress, and adjustment disorders | F43 | 3009 |
|  | Posttraumatic stress disorder | F431 |  |
|  | Obsessive-compulsive disorder | F42 | 3003 |
|  | Stress, not elsewhere classified | Z733 | 308 |
|  | Disorders of initiating and maintaining sleep [insomnias] | G470 | 7805 |
|  | Mental disorder, not otherwise specified | F99 |  |
| 12. Irritable bowel syndrome | Irritable bowel syndrome | K58 | 5641 |
| 13. Alcohol use disorder | Dependence syndrome | **F102** | **3039** |
|  | Alcoholic liver disease | K70 | 291 |
|  | Harmful use | F101 | 303 |
| 14. Other psychoactive substance abuse | Harmful use | F111 | 304 |
|  | Acute intoxication | F100 | 305 |
|  | Dependence syndrome | F112 |  |
|  | Unspecified mental and behavioural disorder | F119 |  |
| 15. Treated constipation | Constipation | K590 | 5640 |
| 16. Stroke/Transient Ischaemic Attack (TIA) | Stroke, not specified as haemorrhage or infarction | I64 | 438 |
|  | Occlusion and stenosis of precerebral arteries, not resulting in cerebral infarction | I65 | 435 |
|  | Subarachnoid haemorrhage | I60 | 430 |
|  | Intracerebral haemorrhage | I61 | 431 |
|  | Other nontraumatic intracranial haemorrhage | I62 | 432 |
|  | Occlusion and stenosis of cerebral arteries, not resulting in cerebral infarction | I66 | 433 |
|  | Cerebral infarction | I63 | 434 |
|  | Acute but ill-defined cerebrovascular disease |  | 436 |
|  | Other and ill-defined cerebrovascular disease |  | 437 |
| 17. Chronic kidney disease | Polycystic kidney, infantile type | Q611 | 75315 |
|  | Polycystic kidney, adult type | Q612 |  |
|  | Polycystic kidney, unspecified | Q613 |  |
|  | Acute renal failure | N17 | 584 |
|  | Chronic renal failure | N18 | 585 |
|  | Unspecified renal failure | N19 | 586 |
|  | Renal complications | E112 | 587 |
|  | Other | N028 | 588 |
| 18. Diverticular disease | Diverticular disease of intestine | K57 | 562 |
| 19. Peripheral vascular disease | Other aneurysm | I72 | 440 |
|  | Other peripheral vascular diseases | I73 | 443 |
|  | Arterial embolism and thrombosis |  | 444 |
| 20. Heart failure | Cardiomyopathy | I42 | 425 |
|  | Heart failure | I50 | 428 |
| 21. Prostate disorders | Hyperplasia of prostate | N40 | 600 |
|  | Inflammatory diseases of prostate | N41 | 601 |
|  | Other disorders of prostate | N42 | 602 |
|  | Disorders of prostate in diseases classified elsewhere | N510 |  |
| 22. Epilepsy | Epilepsy | G40 | 345 |
| 23. Dementia | Creutzfeldt-Jakob disease | A810 | 290 |
|  | Dementia in Alzheimer's disease | F00 |  |
|  | Vascular dementia | F01 |  |
|  | Unspecified dementia | F03 |  |
|  | Delirium superimposed on dementia | F051 |  |
|  | Amnesic syndrome | F106 |  |
|  | Alzheimer's disease | G30 |  |
|  | Other degenerative diseases of nervous system, not elsewhere classified | G31 |  |
|  | Progressive vascular leukoencephalopathy | I673 |  |
| 24. Schizophrenia/bipolar disorder | Schizophrenia | F20 | 295 |
|  | Schizotypal disorder | F21 |  |
|  | Manic episode | F30 | 296 |
|  | Bipolar affective disorder | F31 |  |
| 25. Psoriasis/eczema | Atopic dermatitis | L20 | 696 |
|  | Seborrhoeic dermatitis | L21 | 692 |
|  | Diaper [napkin] dermatitis | L22 |  |
|  | Allergic contact dermatitis | L23 |  |
|  | Irritant contact dermatitis | L24 |  |
|  | Unspecified contact dermatitis | L25 |  |
|  | Exfoliative dermatitis | L26 |  |
|  | Dermatitis due to substances taken internally | L27 |  |
|  | Other dermatitis | L30 |  |
|  | Psoriasis | L40 |  |
|  | Parapsoriasis | L41 |  |
| 26. Inflammatory Bowel Disease | Crohn's disease [regional enteritis] | K50 |  |
|  | Ulcerative colitis | K51 |  |
| 27. Migraine | Migraine | G43 | 346 |
| 28. Bronchiectasis | Bronchiectasis | J47 | 494 |
| 29. Parkinson’s disease | Parkinson's disease | G20 | 332 |
|  | Secondary Parkinsonism | G21 | 3321 |
|  | Parkinsonism in diseases classified elsewhere | G22 | 333 |
|  | Other degenerative diseases of basal ganglia | G23 |  |
|  | Extrapyramidal and movement disorder, unspecified | G259 |  |
|  | Extrapyramidal and movement disorders in diseases classified elsewhere | G26 |  |
|  | Multisystem degeneration | G903 |  |
| 30. Multiple Sclerosis | Multiple sclerosis | G35 | 340 |
| 31. Chronic liver disease | Oesophageal varices | I85 | 571 |
|  | Toxoplasma hepatitis | B581 |  |
|  | Alcoholic hepatitis | K701 |  |
|  | Toxic liver disease with acute hepatitis | K712 |  |
|  | Toxic liver disease with chronic persistent hepatitis | K713 |  |
|  | Toxic liver disease with chronic lobular hepatitis | K714 |  |
|  | Toxic liver disease with chronic active hepatitis | K715 |  |
|  | Toxic liver disease with hepatitis, not elsewhere classified | K716 |  |
|  | Fibrosis and cirrhosis of liver | K74 |  |
|  | Primary biliary cirrhosis | K743 |  |
| 32. Osteoporosis | Polyarthrosis | M15 | 7330 |
|  | Primary generalised (osteo)arthrosis | M150 |  |
|  | Primary generalized (osteo)arthrosis, Multiple sites | M1500 |  |
|  | Heberden's nodes (with arthropathy) | M151 |  |
|  | Coxarthrosis [arthrosis of hip] | M16 |  |
|  | Gonarthrosis [arthrosis of knee] | M17 |  |
| 33. Meniere’s disease | Meniere's disease | H810 | 3860 |
| 34. Pernicious Anaemia | Vitamin B12 deficiency anaemia | D51 | 2810 |
| 35. Heart/cardiac problem | Cardiac arrest | I46 | 393 |
|  | Other cardiac arrhythmias | I49 | 394 |
|  | Complications and ill-defined descriptions of heart disease | I51 | 395 |
|  | Other heart disorders in diseases classified elsewhere | I52 | 396 |
|  | Other cerebrovascular diseases | I67 | 397 |
|  | Cerebrovascular disorders in diseases classified elsewhere | I68 | 398 |
|  | Sequelae of cerebrovascular disease | I69 | 399 |
|  | Atherosclerosis | I70 | 400 |
|  | Acute pericarditis |  | 420 |
|  | Acute and subacute endocarditis |  | 421 |
|  | Acute myocarditis |  | 422 |
|  | Other diseases of pericardium |  | 423 |
|  | Other diseases of endocardium |  | 424 |
|  | Conduction disorders |  | 426 |
|  | Cardiac dysrhythmias |  | 427 |
|  | Ill-defined descriptions and complications of heart disease | | 429 |
| 35. High cholesterol | Disorders of lipoprotein metabolism and other lipidaemias | | E78 |
| 36. Fracture | Multiple fractures of lumbar spine and pelvis | S327 | 808 |
|  | Multiple fractures of lumbar spine and pelvis (closed) | S3270 |  |
|  | Fracture of femur | S72 | 820 |
|  | Fracture of patella | S820 | 8210 |
|  | Fracture of patella (closed) | S8200 | 824 |
| 37. Glaucoma | Glaucoma | H40 | 365 |
| 38. Cataract | Senile cataract | H25 | 366 |
|  | Other cataract | H26 |  |
|  | Cataract and other disorders of lens in diseases classified elsewhere | H28 |  |
| 39. AMD | Degeneration of macula and posterior pole | H353 | 3625 |
| 40. Lung Cancer | Malignant neoplasm of bronchus and lung | C34 | 162 |
| 41. non-melanoma skin cancer | Other malignant neoplasms of skin | C44 | 173 |
|  | Mesothelioma | C45 |  |
| 42. Melanoma | Malignant melanoma of skin | C43 | 172 |
| 43. Stomach Cancer | Malignant neoplasm of stomach | C16 | 151 |
| 44. Oesophageal cancer | Malignant neoplasm of oesophagus | C15 | 150 |
| 45. Colon cancer | Malignant neoplasm of colon | C18 | 153 |
| 46. Rectal cancer | Malignant neoplasm of rectum | C20 | 154 |
| 47. Prostate cancer | Malignant neoplasm of prostate | C61 | 185 |
| 48. ovarian cancer | Malignant neoplasm of ovary | C56 | 183 |
| 49. Breast cancer | Malignant neoplasm of breast | C50 | 174 |
| 50. other cancers | Malignant neoplasm of lip | C00 | 140 |
|  | Malignant neoplasm of base of tongue | C01 | 141 |
|  | Malignant neoplasm of other and unspecified parts of tongue | C02 | 142 |
|  | Malignant neoplasm of gum | C03 | 143 |
|  | Malignant neoplasm of floor of mouth | C04 | 144 |
|  | Malignant neoplasm of palate | C05 | 145 |
|  | Malignant neoplasm of other and unspecified parts of mouth | C06 | 146 |
|  | Malignant neoplasm of parotid gland | C07 | 147 |
|  | Malignant neoplasm of other and unspecified major salivary glands | C08 | 148 |
|  | Malignant neoplasm of tonsil | C09 | 149 |
|  | Malignant neoplasm of oropharynx | C10 | 152 |
|  | Malignant neoplasm of nasopharynx | C11 | 155 |
|  | Malignant neoplasm of pyriform sinus | C12 | 156 |
|  | Malignant neoplasm of hypopharynx | C13 | 157 |
|  | Malignant neoplasm of other and ill-defined sites in the lip, oral cavity and pharynx | C14 | 158 |
|  | Malignant neoplasm of small intestine | C17 | 159 |
|  | Malignant neoplasm of rectosigmoid junction | C19 | 160 |
|  | Malignant neoplasm of anus and anal canal | C21 | 161 |
|  | Malignant neoplasm of liver and intrahepatic bile ducts | C22 | 163 |
|  | Malignant neoplasm of gallbladder | C23 | 164 |
|  | Malignant neoplasm of other and unspecified parts of biliary tract | C24 | 165 |
|  | Malignant neoplasm of pancreas | C25 | 166 |
|  | Malignant neoplasm of other and ill-defined digestive organs | C26 | 167 |
|  | Malignant neoplasm of nasal cavity and middle ear | C30 | 171 |
|  | Malignant neoplasm of accessory sinuses | C31 | 175 |
|  | Malignant neoplasm of larynx | C32 | 176 |
|  | Malignant neoplasm of trachea | C33 | 177 |
|  | Malignant neoplasm of thymus | C37 | 180 |
|  | Malignant neoplasm of heart, mediastinum and pleura | C38 | 181 |
|  | Malignant neoplasm of other and ill-defined sites in the respiratory system and intrathoracic organs | C39 | 184 |
|  | Malignant neoplasm of bone and articular cartilage of limbs | C40 | 186 |
|  | Malignant neoplasm of bone and articular cartilage of other and unspecified sites | C41 | 187 |
|  | hematopoietic and reticuloendothelial systems (ICD-O-3 specific) | C42 | 188 |
|  | Kaposi's sarcoma | C46 | 189 |
|  | Malignant neoplasm of peripheral nerves and autonomic nervous system | C47 | 190 |
|  | Malignant neoplasm of retroperitoneum and peritoneum | C48 | 191 |
|  | Malignant neoplasm of other connective and soft tissue | C49 | 192 |
|  | Malignant neoplasm of vulva | C51 | 193 |
|  | Malignant neoplasm of vagina | C52 | 194 |
|  | Malignant neoplasm of cervix uteri | C53 | 195 |
|  | Malignant neoplasm of corpus uteri | C54 | 196 |
|  | Malignant neoplasm of other and unspecified female genital organs | C57 | 197 |
|  | Malignant neoplasm of placenta | C58 | 198 |
|  | Malignant neoplasm of penis | C60 | 200 |
|  | Malignant neoplasm of testis | C62 | 201 |
|  | Malignant neoplasm of other and unspecified male genital organs | C63 | 202 |
|  | Malignant neoplasm of kidney, except renal pelvis | C64 | 203 |
|  | Malignant neoplasm of renal pelvis | C65 | 204 |
|  | Malignant neoplasm of ureter | C66 | 205 |
|  | Malignant neoplasm of bladder | C67 | 206 |
|  | Malignant neoplasm of other and unspecified urinary organs | C68 | 207 |
|  | Malignant neoplasm of eye and adnexa | C69 | 208 |
|  | Malignant neoplasm of meninges | C70 |  |
|  | Malignant neoplasm of brain | C71 |  |
|  | Malignant neoplasm of spinal cord, cranial nerves and other parts of central nervous system | C72 |  |
|  | Malignant neoplasm of thyroid gland | C73 |  |
|  | Malignant neoplasm of adrenal gland | C74 |  |
|  | Malignant neoplasm of other endocrine glands and related structures | C75 |  |
|  | Malignant neoplasm of other and ill-defined sites | C76 |  |
|  | Secondary and unspecified malignant neoplasm of lymph nodes | C77 |  |
|  | Secondary malignant neoplasm of respiratory and digestive organs | C78 |  |
|  | Secondary malignant neoplasm of other sites | C79 |  |
|  | Malignant neoplasm without specification of site | C80 |  |
|  | Hodgkin's disease | C81 |  |
|  | Follicular [nodular] non-Hodgkin's lymphoma | C82 |  |
|  | Diffuse non-Hodgkin's lymphoma | C83 |  |
|  | Peripheral and cutaneous T-cell lymphomas | C84 |  |
|  | Other and unspecified types of non-Hodgkin's lymphoma | C85 |  |
|  | Other specified types of T/NK-cell lymphoma | C86 |  |
|  | Malignant immunoproliferative diseases | C88 |  |
|  | Multiple myeloma and malignant plasma cell neoplasms | C90 |  |
|  | Lymphoid leukaemia | C91 |  |
|  | Myeloid leukaemia | C92 |  |
|  | Monocytic leukaemia | C93 |  |
|  | Other leukaemias of specified cell type | C94 |  |
|  | Leukaemia of unspecified cell type | C95 |  |
|  | Other and unspecified malignant neoplasms of lymphoid, haematopoietic and related tissue | C96 |  |
|  | Malignant neoplasms of independent (primary) multiple sites | C97 |  |

**Table S3.** **The association between chronological age-adjusted age gap and risk of individual diseases in the validation population**

|  | Chronological age-adjusted age gap* | | | | |  | P-value |
| --- | --- | --- | --- | --- | --- | --- | --- |
|  | Quintile 1 | Quintile 2 | Quintile 3 | Quintile 4 | Quintile 5 |  | for trend |
| Coronary heart disease |  |  |  |  |  |  |  |
| Events | 662 | 650 | 668 | 693 | 734 |  |  |
| Person-years | 123640 | 123490 | 122181 | 120999 | 117648 |  |  |
| HR (95% CI), Model 1^†^ | Reference | 0.98 (0.88-1.09) | 1.02 (0.91-1.13) | 1.07 (0.96-1.19) | 1.16 (1.05-1.29) |  | 0.0024 |
| HR (95% CI), Model 2 | Reference | 1.05 (0.94-1.17) | 1.12 (1.01-1.25) | 1.22 (1.09-1.36) | 1.37 (1.23-1.52) |  | <0.0001 |
| HR (95% CI), Model 3 | Reference | 1.01 (0.90-1.12) | 1.04 (0.93-1.16) | 1.08 (0.97-1.20) | 1.10 (0.99-1.23) |  | 0.33 |
| Heart failure |  |  |  |  |  |  |  |
| Events | 226 | 206 | 230 | 239 | 319 |  |  |
| Person-years | 126759 | 126642 | 125619 | 124437 | 120736 |  |  |
| HR (95% CI), Model 1 | Reference | 0.92 (0.76-1.11) | 1.03 (0.85-1.23) | 1.09 (0.90-1.30) | 1.48 (1.25-1.76) |  | <0.0001 |
| HR (95% CI), Model 2 | Reference | 0.97 (0.80-1.17) | 1.13 (0.94-1.36) | 1.23 (1.02-1.47) | 1.71 (1.44-2.03) |  | <0.0001 |
| HR (95% CI), Model 3 | Reference | 0.92 (0.76-1.11) | 1.03 (0.86-1.24) | 1.06 (0.88-1.28) | 1.31 (1.10-1.57) |  | 0.0018 |
| Atrial fibrillation |  |  |  |  |  |  |  |
| Events | 275 | 282 | 295 | 287 | 336 |  |  |
| Person-years | 124867 | 124401 | 123317 | 122151 | 118192 |  |  |
| HR (95% CI), Model 1 | Reference | 1.02 (0.87-1.21) | 1.09 (0.92-1.28) | 1.07 (0.91-1.26) | 1.28 (1.09-1.50) |  | 0.0025 |
| HR (95% CI), Model 2 | Reference | 1.10 (0.93-1.30) | 1.21 (1.03-1.43) | 1.23 (1.04-1.45) | 1.56 (1.33-1.83) |  | <0.0001 |
| HR (95% CI), Model 3 | Reference | 1.06 (0.89-1.25) | 1.12 (0.95-1.33) | 1.10 (0.93-1.30) | 1.29 (1.09-1.52) |  | 0.0376 |
| Other cardiac disease |  |  |  |  |  |  |  |
| Events | 477 | 494 | 521 | 507 | 628 |  |  |
| Person-years | 125150 | 124548 | 123563 | 122458 | 118746 |  |  |
| HR (95% CI), Model 1 | Reference | 1.04 (0.92-1.18) | 1.10 (0.97-1.25) | 1.09 (0.96-1.23) | 1.39 (1.23-1.57) |  | <0.0001 |
| HR (95% CI), Model 2 | Reference | 1.10 (0.97-1.25) | 1.20 (1.06-1.36) | 1.21 (1.07-1.37) | 1.57 (1.39-1.78) |  | <0.0001 |
| HR (95% CI), Model 3 | Reference | 1.06 (0.93-1.20) | 1.12 (0.99-1.27) | 1.08 (0.96-1.23) | 1.30 (1.15-1.47) |  | 0.0022 |
| Stroke |  |  |  |  |  |  |  |
| Events | 124 | 133 | 156 | 137 | 168 |  |  |
| Person-years | 125257 | 124699 | 124005 | 122804 | 118848 |  |  |
| HR (95% CI), Model 1 | Reference | 1.07 (0.84-1.37) | 1.29 (1.01-1.63) | 1.13 (0.89-1.44) | 1.44 (1.14-1.82) |  | 0.0041 |
| HR (95% CI), Model 2 | Reference | 1.13 (0.89-1.45) | 1.36 (1.07-1.72) | 1.24 (0.97-1.58) | 1.60 (1.26-2.02) |  | 0.0012 |
| HR (95% CI), Model 3 | Reference | 1.11 (0.87-1.42) | 1.30 (1.03-1.65) | 1.16 (0.91-1.48) | 1.42 (1.12-1.80) |  | 0.0364 |
| Peripheral vascular disease |  |  |  |  |  |  |  |
| Events | 135 | 122 | 123 | 130 | 153 |  |  |
| Person-years | 126740 | 126753 | 125849 | 124726 | 121292 |  |  |
| HR (95% CI), Model 1 | Reference | 0.90 (0.70-1.14) | 0.91 (0.71-1.16) | 0.97 (0.76-1.23) | 1.16 (0.92-1.46) |  | 0.13 |
| HR (95% CI), Model 2 | Reference | 0.93 (0.73-1.19) | 0.97 (0.76-1.25) | 1.04 (0.82-1.33) | 1.25 (0.99-1.58) |  | 0.12 |
| HR (95% CI), Model 3 | Reference | 0.91 (0.71-1.17) | 0.94 (0.74-1.20) | 0.97 (0.76-1.24) | 1.09 (0.86-1.39) |  | 0.64 |
| Hypertension |  |  |  |  |  |  |  |
| Events | 691 | 713 | 714 | 738 | 724 |  |  |
| Person-years | 96801 | 94174 | 90910 | 87261 | 78909 |  |  |
| HR (95% CI), Model 1 | Reference | 1.06 (0.95-1.17) | 1.10 (0.99-1.22) | 1.18 (1.06-1.30) | 1.28 (1.15-1.42) |  | <0.0001 |
| HR (95% CI), Model 2 | Reference | 1.11 (1.00-1.23) | 1.19 (1.07-1.32) | 1.31 (1.18-1.45) | 1.46 (1.31-1.62) |  | <0.0001 |
| HR (95% CI), Model 3 | Reference | 1.07 (0.95-1.19) | 1.06 (0.95-1.19) | 1.12 (1.00-1.25) | 1.13 (1.01-1.27) |  | 0.24 |
| Diabetes |  |  |  |  |  |  |  |
| Events | 289 | 325 | 367 | 447 | 554 |  |  |
| Person-years | 123246 | 122453 | 120746 | 118156 | 109325 |  |  |
| HR (95% CI), Model 1 | Reference | 1.14 (0.97-1.33) | 1.30 (1.11-1.52) | 1.62 (1.39-1.88) | 2.16 (1.87-2.49) |  | <0.0001 |
| HR (95% CI), Model 2 | Reference | 1.19 (1.01-1.39) | 1.41 (1.21-1.64) | 1.80 (1.55-2.09) | 2.45 (2.12-2.82) |  | <0.0001 |
| HR (95% CI), Model 3 | Reference | 1.07 (0.92-1.26) | 1.20 (1.02-1.40) | 1.34 (1.15-1.55) | 1.53 (1.32-1.77) |  | <0.0001 |
| Dyslipidemia |  |  |  |  |  |  |  |
| Events | 755 | 817 | 910 | 915 | 1012 |  |  |
| Person-years | 114236 | 111755 | 108061 | 105570 | 97288 |  |  |
| HR (95% CI), Model 1 | Reference | 1.10 (1.00-1.22) | 1.27 (1.15-1.40) | 1.30 (1.18-1.44) | 1.57 (1.43-1.72) |  | <0.0001 |
| HR (95% CI), Model 2 | Reference | 1.18 (1.06-1.30) | 1.43 (1.29-1.57) | 1.50 (1.36-1.65) | 1.87 (1.70-2.05) |  | <0.0001 |
| HR (95% CI), Model 3 | Reference | 1.06 (0.96-1.18) | 1.27 (1.15-1.40) | 1.25 (1.13-1.38) | 1.44 (1.30-1.59) |  | 0.0002 |
| Non-melanoma skin cancer |  |  |  |  |  |  |  |
| Events | 379 | 395 | 414 | 350 | 372 |  |  |
| Person-years | 124852 | 124635 | 123621 | 122755 | 119506 |  |  |
| HR (95% CI), Model 1 | Reference | 1.04 (0.90-1.20) | 1.10 (0.96-1.27) | 0.93 (0.81-1.08) | 1.03 (0.89-1.18) |  | 0.23 |
| HR (95% CI), Model 2 | Reference | 1.06 (0.92-1.23) | 1.14 (0.99-1.31) | 0.99 (0.85-1.14) | 1.11 (0.96-1.29) |  | 0.20 |
| HR (95% CI), Model 3 | Reference | 1.08 (0.94-1.24) | 1.16 (1.01-1.34) | 1.02 (0.88-1.18) | 1.17 (1.01-1.36) |  | 0.0959 |
| Melanoma |  |  |  |  |  |  |  |
| Events | 52 | 82 | 42 | 58 | 66 |  |  |
| Person-years | 126409 | 126186 | 125717 | 124640 | 121495 |  |  |
| HR (95% CI), Model 1 | Reference | 1.58 (1.12-2.23) | 0.81 (0.54-1.22) | 1.13 (0.78-1.64) | 1.32 (0.92-1.90) |  | 0.27 |
| HR (95% CI), Model 2 | Reference | 1.59 (1.12-2.25) | 0.81 (0.54-1.21) | 1.14 (0.78-1.66) | 1.35 (0.94-1.95) |  | 0.18 |
| HR (95% CI), Model 3 | Reference | 1.59 (1.12-2.26) | 0.81 (0.54-1.22) | 1.15 (0.79-1.68) | 1.39 (0.96-2.02) |  | 0.10 |
| Lung cancer |  |  |  |  |  |  |  |
| Events | 92 | 102 | 90 | 87 | 98 |  |  |
| Person-years | 127385 | 127000 | 126221 | 125347 | 122090 |  |  |
| HR (95% CI), Model 1 | Reference | 1.10 (0.83-1.46) | 0.98 (0.73-1.31) | 0.95 (0.71-1.27) | 1.11 (0.84-1.48) |  | 0.75 |
| HR (95% CI), Model 2 | Reference | 1.14 (0.86-1.51) | 1.04 (0.78-1.39) | 1.00 (0.74-1.34) | 1.16 (0.87-1.55) |  | 0.74 |
| HR (95% CI), Model 3 | Reference | 1.15 (0.87-1.53) | 1.05 (0.78-1.41) | 1.01 (0.75-1.36) | 1.17 (0.88-1.57) |  | 0.72 |
| Stomach Cancer |  |  |  |  |  |  |  |
| Events | 30 | 30 | 20 | 17 | 22 |  |  |
| Person-years | 127727 | 127510 | 126709 | 125841 | 122556 |  |  |
| HR (95% CI), Model 1 | Reference | 1.00 (0.60-1.66) | 0.67 (0.38-1.18) | 0.57 (0.32-1.04) | 0.76 (0.44-1.33) |  | 0.94 |
| HR (95% CI), Model 2 | Reference | 1.09 (0.66-1.81) | 0.76 (0.43-1.34) | 0.68 (0.38-1.24) | 0.98 (0.56-1.70) |  | 0.64 |
| HR (95% CI), Model 3 | Reference | 1.07 (0.65-1.78) | 0.73 (0.41-1.29) | 0.66 (0.36-1.20) | 0.91 (0.51-1.61) |  | 0.81 |
| Colon cancer |  |  |  |  |  |  |  |
| Events | 110 | 92 | 110 | 76 | 94 |  |  |
| Person-years | 126761 | 126732 | 125730 | 124972 | 121746 |  |  |
| HR (95% CI), Model 1 | Reference | 0.84 (0.64-1.11) | 1.00 (0.77-1.30) | 0.71 (0.53-0.95) | 0.90 (0.68-1.18) |  | 0.3607 |
| HR (95% CI), Model 2 | Reference | 0.86 (0.65-1.13) | 1.04 (0.80-1.36) | 0.74 (0.55-0.99) | 0.95 (0.72-1.25) |  | 0.5722 |
| HR (95% CI), Model 3 | Reference | 0.84 (0.63-1.11) | 1.01 (0.77-1.31) | 0.70 (0.52-0.94) | 0.86 (0.65-1.15) |  | 0.1196 |
| Oesophageal cancer |  |  |  |  |  |  |  |
| Events | 22 | 31 | 32 | 29 | 37 |  |  |
| Person-years | 127779 | 127483 | 126652 | 125760 | 122529 |  |  |
| HR (95% CI), Model 1 | Reference | 1.41 (0.82-2.43) | 1.46 (0.85-2.52) | 1.33 (0.77-2.32) | 1.75 (1.03-2.97) |  | 0.0492 |
| HR (95% CI), Model 2 | Reference | 1.51 (0.87-2.60) | 1.64 (0.95-2.83) | 1.57 (0.90-2.75) | 2.17 (1.27-3.69) |  | 0.0154 |
| HR (95% CI), Model 3 | Reference | 1.51 (0.87-2.61) | 1.66 (0.96-2.87) | 1.61 (0.92-2.82) | 2.27 (1.32-3.89) |  | 0.0134 |
| Rectal cancer |  |  |  |  |  |  |  |
| Events | 34 | 36 | 46 | 44 | 38 |  |  |
| Person-years | 127488 | 127265 | 126408 | 125521 | 122233 |  |  |
| HR (95% CI), Model 1 | Reference | 1.06 (0.66-1.70) | 1.37 (0.88-2.13) | 1.29 (0.82-2.02) | 1.14 (0.72-1.82) |  | 0.45 |
| HR (95% CI), Model 2 | Reference | 1.13 (0.70-1.80) | 1.48 (0.95-2.30) | 1.48 (0.95-2.32) | 1.38 (0.86-2.20) |  | 0.17 |
| HR (95% CI), Model 3 | Reference | 1.12 (0.70-1.79) | 1.46 (0.94-2.28) | 1.44 (0.92-2.27) | 1.29 (0.80-2.08) |  | 0.43 |
| Prostate cancer^‡^ |  |  |  |  |  |  |  |
| Events | 270 | 243 | 238 | 175 | 161 |  |  |
| Person-years | 70060 | 62725 | 59000 | 54302 | 47609 |  |  |
| HR (95% CI), Model 1 | Reference | 1.00 (0.84-1.19) | 1.05 (0.88-1.25) | 0.82 (0.68-0.99) | 0.87 (0.72-1.06) |  | 0.0754 |
| HR (95% CI), Model 2 | Reference | 1.02 (0.86-1.21) | 1.07 (0.90-1.28) | 0.85 (0.70-1.03) | 0.89 (0.73-1.08) |  | 0.11 |
| HR (95% CI), Model 3 | Reference | 1.02 (0.86-1.22) | 1.07 (0.90-1.28) | 0.86 (0.71-1.04) | 0.91 (0.74-1.11) |  | 0.18 |
| Ovarian cancer^¶^ |  |  |  |  |  |  |  |
| Events | 26 | 22 | 36 | 29 | 29 |  |  |
| Person-years | 59188 | 66910 | 70841 | 75947 | 82633 |  |  |
| HR (95% CI), Model 1 | Reference | 0.75 (0.42-1.32) | 1.16 (0.70-1.91) | 0.87 (0.51-1.47) | 0.77 (0.45-1.32) |  | 0.41 |
| HR (95% CI), Model 2 | Reference | 0.74 (0.42-1.31) | 1.17 (0.71-1.95) | 0.87 (0.51-1.49) | 0.79 (0.46-1.34) |  | 0.40 |
| HR (95% CI), Model 3 | Reference | 0.74 (0.42-1.31) | 1.16 (0.70-1.92) | 0.86 (0.50-1.46) | 0.76 (0.44-1.31) |  | 0.38 |
| Breast cancer^¶^ |  |  |  |  |  |  |  |
| Events | 159 | 193 | 216 | 227 | 230 |  |  |
| Person-years | 56450 | 63207 | 67047 | 71863 | 77954 |  |  |
| HR (95% CI), Model 1 | Reference | 1.08 (0.87-1.33) | 1.14 (0.93-1.41) | 1.12 (0.91-1.37) | 1.04 (0.85-1.28) |  | 0.70 |
| HR (95% CI), Model 2 | Reference | 1.08 (0.87-1.33) | 1.14 (0.93-1.40) | 1.11 (0.91-1.36) | 1.05 (0.85-1.28) |  | 0.74 |
| HR (95% CI), Model 3 | Reference | 1.07 (0.86-1.32) | 1.11 (0.90-1.36) | 1.08 (0.88-1.33) | 1.00 (0.81-1.23) |  | 0.77 |
| Other cancer |  |  |  |  |  |  |  |
| Events | 597 | 623 | 623 | 615 | 664 |  |  |
| Person-years | 119065 | 118247 | 117213 | 116431 | 111962 |  |  |
| HR (95% CI), Model 1 | Reference | 1.05 (0.94-1.18) | 1.05 (0.94-1.18) | 1.05 (0.94-1.17) | 1.18 (1.06-1.32) |  | 0.0210 |
| HR (95% CI), Model 2 | Reference | 1.06 (0.95-1.19) | 1.08 (0.96-1.20) | 1.07 (0.96-1.20) | 1.22 (1.09-1.36) |  | 0.0083 |
| HR (95% CI), Model 3 | Reference | 1.05 (0.94-1.17) | 1.05 (0.94-1.18) | 1.04 (0.92-1.16) | 1.14 (1.02-1.28) |  | 0.0830 |
| Depression |  |  |  |  |  |  |  |
| Events | 185 | 212 | 207 | 217 | 237 |  |  |
| Person-years | 119985 | 119225 | 118488 | 116994 | 112138 |  |  |
| HR (95% CI), Model 1 | Reference | 1.14 (0.93-1.39) | 1.11 (0.91-1.36) | 1.19 (0.98-1.45) | 1.34 (1.11-1.63) |  | 0.0347 |
| HR (95% CI), Model 2 | Reference | 1.10 (0.91-1.35) | 1.09 (0.90-1.33) | 1.13 (0.93-1.37) | 1.21 (1.00-1.47) |  | 0.38 |
| HR (95% CI), Model 3 | Reference | 1.07 (0.88-1.30) | 1.03 (0.84-1.26) | 1.03 (0.85-1.26) | 1.04 (0.85-1.27) |  | 0.81 |
| Anxiety |  |  |  |  |  |  |  |
| Events | 374 | 409 | 351 | 397 | 467 |  |  |
| Person-years | 123880 | 123489 | 122942 | 121744 | 117840 |  |  |
| HR (95% CI), Model 1 | Reference | 1.09 (0.95-1.26) | 0.94 (0.81-1.09) | 1.07 (0.93-1.23) | 1.31 (1.14-1.50) |  | 0.0062 |
| HR (95% CI), Model 2 | Reference | 1.04 (0.91-1.20) | 0.89 (0.77-1.03) | 0.99 (0.86-1.14) | 1.14 (0.99-1.30) |  | 0.37 |
| HR (95% CI), Model 3 | Reference | 1.02 (0.89-1.18) | 0.86 (0.74-1.00) | 0.94 (0.81-1.08) | 1.03 (0.90-1.19) |  | 0.84 |
| Schizophrenia |  |  |  |  |  |  |  |
| Events | 20 | 23 | 16 | 25 | 32 |  |  |
| Person-years | 127184 | 127048 | 126254 | 125263 | 121853 |  |  |
| HR (95% CI), Model 1 | Reference | 1.15 (0.63-2.09) | 0.81 (0.42-1.56) | 1.27 (0.70-2.28) | 1.62 (0.92-2.84) |  | 0.64 |
| HR (95% CI), Model 2 | Reference | 1.14 (0.63-2.08) | 0.81 (0.42-1.56) | 1.27 (0.70-2.29) | 1.55 (0.88-2.73) |  | 0.66 |
| HR (95% CI), Model 3 | Reference | 1.09 (0.60-1.99) | 0.73 (0.38-1.42) | 1.12 (0.61-2.03) | 1.23 (0.68-2.21) |  | 0.90 |
| Alcohol problems |  |  |  |  |  |  |  |
| Events | 107 | 98 | 99 | 112 | 140 |  |  |
| Person-years | 126763 | 126725 | 125907 | 124920 | 121488 |  |  |
| HR (95% CI), Model 1 | Reference | 0.92 (0.70-1.22) | 0.94 (0.71-1.24) | 1.07 (0.82-1.40) | 1.37 (1.06-1.76) |  | 0.0132 |
| HR (95% CI), Model 2 | Reference | 0.98 (0.74-1.29) | 1.05 (0.80-1.38) | 1.21 (0.93-1.58) | 1.64 (1.27-2.11) |  | 0.0036 |
| HR (95% CI), Model 3 | Reference | 0.96 (0.73-1.26) | 1.02 (0.77-1.34) | 1.15 (0.88-1.50) | 1.49 (1.15-1.94) |  | 0.0366 |
| Psychoactive substance abuse |  |  |  |  |  |  |  |
| Events | 35 | 31 | 31 | 27 | 45 |  |  |
| Person-years | 127465 | 127325 | 126508 | 125558 | 122199 |  |  |
| HR (95% CI), Model 1 | Reference | 0.89 (0.55-1.44) | 0.89 (0.55-1.45) | 0.75 (0.45-1.25) | 1.31 (0.84-2.05) |  | 0.19 |
| HR (95% CI), Model 2 | Reference | 0.97 (0.59-1.57) | 1.00 (0.61-1.62) | 0.90 (0.54-1.49) | 1.59 (1.01-2.48) |  | 0.38 |
| HR (95% CI), Model 3 | Reference | 0.97 (0.60-1.57) | 1.01 (0.62-1.65) | 0.90 (0.54-1.50) | 1.57 (0.99-2.49) |  | 0.53 |
| Dementia |  |  |  |  |  |  |  |
| Events | 133 | 139 | 132 | 135 | 144 |  |  |
| Person-years | 127439 | 127132 | 126308 | 125522 | 122227 |  |  |
| HR (95% CI), Model 1 | Reference | 1.03 (0.81-1.31) | 0.99 (0.78-1.26) | 1.02 (0.80-1.30) | 1.12 (0.88-1.42) |  | 0.0936 |
| HR (95% CI), Model 2 | Reference | 1.10 (0.86-1.39) | 1.07 (0.84-1.37) | 1.16 (0.91-1.47) | 1.25 (0.98-1.58) |  | 0.0615 |
| HR (95% CI), Model 3 | Reference | 1.08 (0.85-1.37) | 1.04 (0.81-1.32) | 1.09 (0.85-1.38) | 1.10 (0.86-1.40) |  | 0.45 |
| Parkinson's disease |  |  |  |  |  |  |  |
| Events | 69 | 60 | 49 | 65 | 53 |  |  |
| Person-years | 127395 | 127255 | 126445 | 125579 | 122466 |  |  |
| HR (95% CI), Model 1 | Reference | 0.87 (0.62-1.23) | 0.72 (0.50-1.03) | 0.96 (0.68-1.34) | 0.80 (0.56-1.14) |  | 0.78 |
| HR (95% CI), Model 2 | Reference | 0.92 (0.65-1.30) | 0.78 (0.54-1.13) | 1.08 (0.77-1.52) | 0.93 (0.65-1.34) |  | 0.57 |
| HR (95% CI), Model 3 | Reference | 0.92 (0.65-1.30) | 0.77 (0.53-1.11) | 1.05 (0.74-1.48) | 0.88 (0.60-1.27) |  | 0.82 |
| Multiple sclerosis |  |  |  |  |  |  |  |
| Events | 7 | 11 | 10 | 7 | 11 |  |  |
| Person-years | 127555 | 127174 | 126408 | 125427 | 122270 |  |  |
| HR (95% CI), Model 1 | Reference | 1.57 (0.61-4.06) | 1.30 (0.48-3.48) | 1.02 (0.36-2.89) | 1.64 (0.63-4.22) |  | 0.89 |
| HR (95% CI), Model 2 | Reference | 1.64 (0.65-4.18) | 1.31 (0.49-3.45) | 0.78 (0.26-2.33) | 1.33 (0.51-3.51) |  | 0.86 |
| HR (95% CI), Model 3 | Reference | 1.50 (0.58-3.88) | 1.36 (0.52-3.60) | 0.93 (0.32-2.68) | 1.33 (0.50-3.53) |  | 0.80 |
| Migraine |  |  |  |  |  |  |  |
| Events | 79 | 62 | 79 | 83 | 82 |  |  |
| Person-years | 123611 | 123536 | 123093 | 121417 | 118084 |  |  |
| HR (95% CI), Model 1 | Reference | 0.78 (0.56-1.09) | 1.00 (0.73-1.37) | 1.07 (0.78-1.45) | 1.07 (0.79-1.46) |  | 0.20 |
| HR (95% CI), Model 2 | Reference | 0.74 (0.53-1.04) | 0.93 (0.68-1.27) | 0.97 (0.71-1.32) | 0.92 (0.68-1.26) |  | 0.47 |
| HR (95% CI), Model 3 | Reference | 0.73 (0.52-1.02) | 0.90 (0.66-1.23) | 0.92 (0.67-1.25) | 0.85 (0.62-1.17) |  | 0.90 |
| Epilepsy |  |  |  |  |  |  |  |
| Events | 60 | 50 | 66 | 55 | 72 |  |  |
| Person-years | 126121 | 125950 | 125608 | 124588 | 121382 |  |  |
| HR (95% CI), Model 1 | Reference | 0.82 (0.56-1.19) | 1.10 (0.78-1.57) | 0.89 (0.62-1.29) | 1.25 (0.89-1.76) |  | 0.0926 |
| HR (95% CI), Model 2 | Reference | 0.86 (0.59-1.25) | 1.15 (0.81-1.64) | 0.98 (0.68-1.42) | 1.32 (0.94-1.87) |  | 0.11 |
| HR (95% CI), Model 3 | Reference | 0.84 (0.58-1.23) | 1.11 (0.78-1.58) | 0.93 (0.64-1.35) | 1.20 (0.84-1.71) |  | 0.18 |
| COPD |  |  |  |  |  |  |  |
| Events | 294 | 305 | 288 | 323 | 352 |  |  |
| Person-years | 125136 | 124414 | 123633 | 122369 | 118683 |  |  |
| HR (95% CI), Model 1 | Reference | 1.05 (0.89-1.23) | 1.00 (0.85-1.17) | 1.12 (0.95-1.31) | 1.24 (1.06-1.46) |  | 0.0003 |
| HR (95% CI), Model 2 | Reference | 1.10 (0.94-1.30) | 1.09 (0.93-1.28) | 1.23 (1.05-1.45) | 1.37 (1.17-1.60) |  | 0.0007 |
| HR (95% CI), Model 3 | Reference | 1.08 (0.92-1.27) | 1.05 (0.89-1.24) | 1.17 (1.00-1.37) | 1.23 (1.05-1.45) |  | 0.0233 |
| Asthma |  |  |  |  |  |  |  |
| Events | 223 | 233 | 217 | 226 | 273 |  |  |
| Person-years | 112187 | 112212 | 111897 | 109542 | 105028 |  |  |
| HR (95% CI), Model 1 | Reference | 1.04 (0.87-1.25) | 0.97 (0.80-1.17) | 1.03 (0.86-1.24) | 1.30 (1.09-1.56) |  | 0.0081 |
| HR (95% CI), Model 2 | Reference | 1.04 (0.86-1.24) | 0.97 (0.81-1.17) | 1.02 (0.85-1.23) | 1.26 (1.05-1.51) |  | 0.0631 |
| HR (95% CI), Model 3 | Reference | 1.01 (0.84-1.21) | 0.92 (0.77-1.12) | 0.95 (0.79-1.14) | 1.10 (0.92-1.32) |  | 0.49 |
| Bronchiectasis |  |  |  |  |  |  |  |
| Events | 94 | 75 | 83 | 76 | 86 |  |  |
| Person-years | 127266 | 127016 | 126197 | 125315 | 122080 |  |  |
| HR (95% CI), Model 1 | Reference | 0.80 (0.59-1.08) | 0.88 (0.65-1.18) | 0.81 (0.60-1.10) | 0.93 (0.69-1.25) |  | 0.51 |
| HR (95% CI), Model 2 | Reference | 0.79 (0.58-1.07) | 0.90 (0.67-1.21) | 0.82 (0.61-1.11) | 0.94 (0.70-1.26) |  | 0.40 |
| HR (95% CI), Model 3 | Reference | 0.81 (0.60-1.10) | 0.93 (0.69-1.25) | 0.86 (0.63-1.17) | 1.00 (0.74-1.35) |  | 0.60 |
| Dyspepsia |  |  |  |  |  |  |  |
| Events | 1292 | 1293 | 1272 | 1241 | 1386 |  |  |
| Person-years | 107528 | 106632 | 106522 | 106151 | 100648 |  |  |
| HR (95% CI), Model 1 | Reference | 1.01 (0.94-1.09) | 0.99 (0.92-1.07) | 0.97 (0.90-1.05) | 1.15 (1.06-1.24) |  | 0.0000 |
| HR (95% CI), Model 2 | Reference | 1.01 (0.93-1.09) | 0.99 (0.92-1.07) | 0.97 (0.89-1.04) | 1.12 (1.03-1.21) |  | 0.0044 |
| HR (95% CI), Model 3 | Reference | 0.98 (0.91-1.06) | 0.95 (0.88-1.03) | 0.91 (0.84-0.99) | 1.02 (0.94-1.10) |  | 0.33 |
| Constipation |  |  |  |  |  |  |  |
| Events | 461 | 403 | 406 | 443 | 477 |  |  |
| Person-years | 124588 | 124873 | 123667 | 122985 | 119040 |  |  |
| HR (95% CI), Model 1 | Reference | 0.88 (0.77-1.00) | 0.89 (0.78-1.01) | 0.98 (0.86-1.11) | 1.08 (0.95-1.23) |  | 0.0675 |
| HR (95% CI), Model 2 | Reference | 0.87 (0.76-1.00) | 0.90 (0.78-1.02) | 0.99 (0.87-1.12) | 1.08 (0.95-1.23) |  | 0.14 |
| HR (95% CI), Model 3 | Reference | 0.86 (0.75-0.98) | 0.87 (0.76-1.00) | 0.94 (0.83-1.08) | 0.99 (0.87-1.13) |  | 0.89 |
| Diverticulitis |  |  |  |  |  |  |  |
| Events | 934 | 938 | 983 | 1021 | 1031 |  |  |
| Person-years | 120124 | 119602 | 118503 | 117424 | 113985 |  |  |
| HR (95% CI), Model 1 | Reference | 1.01 (0.92-1.10) | 1.06 (0.97-1.16) | 1.12 (1.02-1.22) | 1.16 (1.06-1.27) |  | 0.0005 |
| HR (95% CI), Model 2 | Reference | 1.01 (0.93-1.11) | 1.08 (0.98-1.18) | 1.13 (1.03-1.24) | 1.17 (1.07-1.28) |  | 0.0051 |
| HR (95% CI), Model 3 | Reference | 0.99 (0.91-1.09) | 1.04 (0.95-1.13) | 1.07 (0.98-1.17) | 1.07 (0.97-1.17) |  | 0.32 |
| Irritable bowel syndrome |  |  |  |  |  |  |  |
| Events | 155 | 119 | 166 | 134 | 181 |  |  |
| Person-years | 124233 | 123576 | 122476 | 121803 | 118219 |  |  |
| HR (95% CI), Model 1 | Reference | 0.77 (0.61-0.98) | 1.09 (0.87-1.35) | 0.88 (0.69-1.10) | 1.23 (0.99-1.52) |  | 0.14 |
| HR (95% CI), Model 2 | Reference | 0.72 (0.57-0.92) | 0.99 (0.80-1.24) | 0.79 (0.62-0.99) | 1.02 (0.82-1.26) |  | 0.83 |
| HR (95% CI), Model 3 | Reference | 0.72 (0.56-0.91) | 0.97 (0.78-1.21) | 0.76 (0.60-0.96) | 0.96 (0.77-1.20) |  | 0.73 |
| Inflammatory bowel disease | |  |  |  |  |  |  |
| Events | 79 | 55 | 56 | 65 | 67 |  |  |
| Person-years | 126379 | 126064 | 125287 | 124180 | 121044 |  |  |
| HR (95% CI), Model 1 | Reference | 0.70 (0.49-0.98) | 0.71 (0.51-1.01) | 0.84 (0.60-1.16) | 0.88 (0.64-1.22) |  | 0.88 |
| HR (95% CI), Model 2 | Reference | 0.71 (0.50-1.00) | 0.74 (0.53-1.05) | 0.88 (0.63-1.23) | 0.94 (0.67-1.30) |  | 1.00 |
| HR (95% CI), Model 3 | Reference | 0.71 (0.50-1.01) | 0.75 (0.53-1.06) | 0.89 (0.64-1.24) | 0.94 (0.67-1.32) |  | 0.93 |
| Chronic liver disease |  |  |  |  |  |  |  |
| Events | 32 | 27 | 33 | 43 | 58 |  |  |
| Person-years | 126750 | 126652 | 125820 | 124667 | 121448 |  |  |
| HR (95% CI), Model 1 | Reference | 0.87 (0.52-1.46) | 1.04 (0.63-1.70) | 1.41 (0.89-2.23) | 1.91 (1.23-2.96) |  | 0.0021 |
| HR (95% CI), Model 2 | Reference | 0.89 (0.53-1.48) | 1.13 (0.69-1.83) | 1.53 (0.97-2.42) | 2.12 (1.37-3.27) |  | 0.0004 |
| HR (95% CI), Model 3 | Reference | 0.87 (0.52-1.46) | 1.09 (0.67-1.77) | 1.43 (0.90-2.28) | 1.82 (1.16-2.85) |  | 0.0110 |
| CKD |  |  |  |  |  |  |  |
| Events | 486 | 482 | 551 | 609 | 776 |  |  |
| Person-years | 125729 | 125424 | 124284 | 123045 | 118333 |  |  |
| HR (95% CI), Model 1 | Reference | 1.00 (0.88-1.13) | 1.14 (1.01-1.29) | 1.29 (1.14-1.45) | 1.70 (1.52-1.91) |  | <0.0001 |
| HR (95% CI), Model 2 | Reference | 1.03 (0.91-1.17) | 1.24 (1.10-1.40) | 1.42 (1.26-1.60) | 1.91 (1.70-2.14) |  | <0.0001 |
| HR (95% CI), Model 3 | Reference | 0.98 (0.86-1.11) | 1.12 (0.99-1.26) | 1.20 (1.06-1.35) | 1.44 (1.28-1.62) |  | <0.0001 |
| Osteoporosis |  |  |  |  |  |  |  |
| Events | 769 | 805 | 812 | 809 | 874 |  |  |
| Person-years | 112819 | 111943 | 110456 | 109702 | 105479 |  |  |
| HR (95% CI), Model 1 | Reference | 1.05 (0.95-1.16) | 1.08 (0.98-1.19) | 1.08 (0.98-1.19) | 1.21 (1.10-1.34) |  | 0.0022 |
| HR (95% CI), Model 2 | Reference | 1.05 (0.95-1.16) | 1.07 (0.97-1.19) | 1.06 (0.96-1.17) | 1.18 (1.07-1.30) |  | 0.0186 |
| HR (95% CI), Model 3 | Reference | 1.00 (0.91-1.11) | 0.99 (0.89-1.09) | 0.95 (0.86-1.05) | 0.99 (0.89-1.09) |  | 0.83 |
| Fracture |  |  |  |  |  |  |  |
| Events | 113 | 97 | 104 | 103 | 105 |  |  |
| Person-years | 126801 | 126774 | 125751 | 125080 | 121745 |  |  |
| HR (95% CI), Model 1 | Reference | 0.86 (0.65-1.13) | 0.92 (0.70-1.20) | 0.92 (0.71-1.21) | 0.97 (0.74-1.26) |  | 0.52 |
| HR (95% CI), Model 2 | Reference | 0.83 (0.63-1.09) | 0.89 (0.68-1.16) | 0.87 (0.66-1.14) | 0.89 (0.68-1.16) |  | 0.92 |
| HR (95% CI), Model 3 | Reference | 0.85 (0.65-1.11) | 0.92 (0.71-1.21) | 0.90 (0.69-1.18) | 0.93 (0.71-1.22) |  | 0.98 |
| Glaucoma |  |  |  |  |  |  |  |
| Events | 190 | 176 | 178 | 193 | 184 |  |  |
| Person-years | 125521 | 125797 | 124526 | 123807 | 120580 |  |  |
| HR (95% CI), Model 1 | Reference | 0.92 (0.75-1.13) | 0.93 (0.76-1.15) | 1.03 (0.84-1.26) | 1.01 (0.82-1.23) |  | 0.75 |
| HR (95% CI), Model 2 | Reference | 0.93 (0.76-1.15) | 0.96 (0.78-1.18) | 1.06 (0.87-1.29) | 1.03 (0.84-1.27) |  | 0.78 |
| HR (95% CI), Model 3 | Reference | 0.93 (0.76-1.15) | 0.96 (0.78-1.18) | 1.05 (0.86-1.29) | 1.01 (0.82-1.24) |  | 0.89 |
| Cataract |  |  |  |  |  |  |  |
| Events | 881 | 900 | 919 | 873 | 881 |  |  |
| Person-years | 121112 | 120785 | 120173 | 119187 | 115319 |  |  |
| HR (95% CI), Model 1 | Reference | 1.02 (0.93-1.12) | 1.05 (0.95-1.15) | 1.00 (0.91-1.10) | 1.05 (0.95-1.15) |  | 0.0619 |
| HR (95% CI), Model 2 | Reference | 1.03 (0.94-1.13) | 1.06 (0.97-1.16) | 1.01 (0.92-1.11) | 1.04 (0.94-1.14) |  | 0.30 |
| HR (95% CI), Model 3 | Reference | 1.01 (0.92-1.11) | 1.03 (0.93-1.13) | 0.96 (0.87-1.05) | 0.95 (0.86-1.04) |  | 0.15 |
| AMD |  |  |  |  |  |  |  |
| Events | 138 | 127 | 135 | 137 | 174 |  |  |
| Person-years | 127084 | 126947 | 126152 | 125095 | 121801 |  |  |
| HR (95% CI), Model 1 | Reference | 0.92 (0.72-1.17) | 0.98 (0.77-1.24) | 1.01 (0.79-1.27) | 1.30 (1.04-1.62) |  | 0.0038 |
| HR (95% CI), Model 2 | Reference | 0.92 (0.72-1.17) | 0.98 (0.77-1.25) | 0.99 (0.78-1.26) | 1.28 (1.02-1.61) |  | 0.0163 |
| HR (95% CI), Model 3 | Reference | 0.91 (0.71-1.16) | 0.96 (0.76-1.23) | 0.96 (0.76-1.23) | 1.22 (0.97-1.53) |  | 0.0701 |
| Pernicious anaemia |  |  |  |  |  |  |  |
| Events | 26 | 24 | 29 | 18 | 24 |  |  |
| Person-years | 127367 | 127187 | 126391 | 125546 | 122154 |  |  |
| HR (95% CI), Model 1 | Reference | 0.92 (0.53-1.61) | 1.08 (0.64-1.85) | 0.70 (0.38-1.28) | 0.96 (0.55-1.68) |  | 0.94 |
| HR (95% CI), Model 2 | Reference | 0.92 (0.53-1.60) | 1.13 (0.66-1.92) | 0.70 (0.38-1.28) | 0.92 (0.53-1.62) |  | 0.99 |
| HR (95% CI), Model 3 | Reference | 0.88 (0.50-1.53) | 1.03 (0.61-1.76) | 0.60 (0.33-1.11) | 0.68 (0.38-1.22) |  | 0.69 |
| Thyroid disorders |  |  |  |  |  |  |  |
| Events | 235 | 259 | 253 | 294 | 319 |  |  |
| Person-years | 120527 | 119318 | 118685 | 116458 | 112154 |  |  |
| HR (95% CI), Model 1 | Reference | 1.11 (0.93-1.32) | 1.09 (0.91-1.30) | 1.28 (1.08-1.52) | 1.45 (1.23-1.72) |  | 0.0000 |
| HR (95% CI), Model 2 | Reference | 1.04 (0.87-1.24) | 1.01 (0.85-1.21) | 1.16 (0.98-1.38) | 1.23 (1.03-1.45) |  | 0.0014 |
| HR (95% CI), Model 3 | Reference | 1.01 (0.85-1.21) | 0.96 (0.80-1.15) | 1.08 (0.91-1.28) | 1.08 (0.91-1.28) |  | 0.0843 |
| Eczema |  |  |  |  |  |  |  |
| Events | 125 | 137 | 149 | 135 | 159 |  |  |
| Person-years | 122644 | 122362 | 121530 | 120398 | 116931 |  |  |
| HR (95% CI), Model 1 | Reference | 1.10 (0.86-1.40) | 1.20 (0.94-1.52) | 1.09 (0.86-1.39) | 1.33 (1.05-1.68) |  | 0.0112 |
| HR (95% CI), Model 2 | Reference | 1.10 (0.86-1.40) | 1.20 (0.95-1.53) | 1.11 (0.87-1.42) | 1.31 (1.04-1.66) |  | 0.0519 |
| HR (95% CI), Model 3 | Reference | 1.07 (0.84-1.36) | 1.15 (0.90-1.46) | 1.03 (0.80-1.32) | 1.16 (0.91-1.48) |  | 0.40 |
| Meniere disease |  |  |  |  |  |  |  |
| Events | 18 | 10 | 20 | 7 | 13 |  |  |
| Person-years | 127409 | 127215 | 126367 | 125414 | 122237 |  |  |
| HR (95% CI), Model 1 | Reference | 0.56 (0.26-1.21) | 1.12 (0.60-2.13) | 0.40 (0.17-0.95) | 0.76 (0.37-1.55) |  | 0.15 |
| HR (95% CI), Model 2 | Reference | 0.55 (0.25-1.18) | 1.11 (0.58-2.10) | 0.39 (0.16-0.93) | 0.72 (0.35-1.49) |  | 0.14 |
| HR (95% CI), Model 3 | Reference | 0.52 (0.24-1.14) | 1.04 (0.55-1.97) | 0.35 (0.15-0.85) | 0.61 (0.29-1.29) |  | 0.0424 |
| Prostate disorders^‡^ |  |  |  |  |  |  |  |
| Events | 497 | 455 | 418 | 378 | 327 |  |  |
| Person-years | 65178 | 58800 | 54762 | 50623 | 44224 |  |  |
| HR (95% CI), Model 1 | Reference | 1.01 (0.89-1.15) | 1.00 (0.87-1.13) | 0.98 (0.85-1.12) | 0.97 (0.84-1.11) |  | 0.97 |
| HR (95% CI), Model 2 | Reference | 1.02 (0.90-1.16) | 1.01 (0.88-1.15) | 0.97 (0.85-1.11) | 0.93 (0.81-1.07) |  | 0.78 |
| HR (95% CI), Model 3 | Reference | 1.00 (0.88-1.14) | 0.97 (0.85-1.11) | 0.94 (0.82-1.07) | 0.88 (0.76-1.02) |  | 0.39 |

AMD, age related macular degeneration; CI, confidence interval; CKD, chronic kidney disease; COPD, chronic obstructive pulmonary disease; HR, hazard ratio.

*Age gap was calculated by subtracting chronological age from metabolomic age. Chronological age-adjusted age gap was calculated with use of regression models.

^†^Cox proportional regression models were used to examine the association between chronological age-adjusted age gap and incidence of individual chronic diseases. Model 1 was unadjusted; Model 2 was adjusted for Model 1 plus age, sex, ethnicity, education, household income, diet score, alcohol consumption, physical activity, smoking, sleep duration, fasting duration, and GRS for longevity; Model 3 was adjusted for Model 2 plus BMI, high cholesterol, hypertension, and antihypertensive and lipid-lowering medications (hypertension or antihypertensive medication use at baseline was not adjusted for the analysis of incident hypertension given these participants with hypertension or antihypertensive medication use were excluded from the analysis).

^‡^These analyses were conducted among men only.

^¶^These analyses were conducted among women only.

**Table S4.** **The association between chronological age-adjusted age gap and risk of individual diseases in validation population with follow-up duration of ≥1 years**

|  | Chronological age-adjusted age gap* | | | | |  | P-value  for trend |
| --- | --- | --- | --- | --- | --- | --- | --- |
|  | Quintile 1 | Quintile 2 | Quintile 3 | Quintile 4 | Quintile 5 |  |  |
| Coronary heart disease |  |  |  |  |  |  |  |
| Events | 629 | 616 | 635 | 669 | 704 |  |  |
| Person-years | 123623 | 123471 | 122163 | 120983 | 117632 |  |  |
| HR (95% CI), Model 1^†^ | Reference | 0.98 (0.87-1.09) | 1.02 (0.91-1.14) | 1.08 (0.97-1.21) | 1.18 (1.06-1.31) |  | 0.0011 |
| HR (95% CI), Model 2 | Reference | 1.04 (0.93-1.17) | 1.12 (1.00-1.25) | 1.24 (1.11-1.38) | 1.38 (1.24-1.54) |  | <0.0001 |
| HR (95% CI), Model 3 | Reference | 1.00 (0.90-1.12) | 1.04 (0.93-1.16) | 1.09 (0.98-1.22) | 1.12 (1.00-1.25) |  | 0.0197 |
| Heart failure |  |  |  |  |  |  |  |
| Events | 219 | 203 | 225 | 230 | 312 |  |  |
| Person-years | 126757 | 126641 | 125616 | 124433 | 120732 |  |  |
| HR (95% CI), Model 1 | Reference | 0.94 (0.77-1.13) | 1.04 (0.86-1.25) | 1.08 (0.90-1.30) | 1.50 (1.26-1.78) |  | <0.0001 |
| HR (95% CI), Model 2 | Reference | 0.98 (0.81-1.19) | 1.14 (0.95-1.38) | 1.22 (1.01-1.47) | 1.72 (1.45-2.05) |  | <0.0001 |
| HR (95% CI), Model 3 | Reference | 0.94 (0.77-1.14) | 1.04 (0.87-1.26) | 1.05 (0.87-1.27) | 1.32 (1.10-1.59) |  | 0.0009 |
| Atrial fibrillation |  |  |  |  |  |  |  |
| Events | 262 | 263 | 264 | 265 | 298 |  |  |
| Person-years | 124861 | 124392 | 123303 | 122139 | 118173 |  |  |
| HR (95% CI), Model 1 | Reference | 1.00 (0.84-1.19) | 1.02 (0.86-1.21) | 1.04 (0.87-1.23) | 1.19 (1.01-1.41) |  | 0.0381 |
| HR (95% CI), Model 2 | Reference | 1.08 (0.91-1.28) | 1.14 (0.96-1.35) | 1.20 (1.01-1.42) | 1.46 (1.23-1.73) |  | 0.0000 |
| HR (95% CI), Model 3 | Reference | 1.04 (0.87-1.23) | 1.06 (0.89-1.26) | 1.07 (0.90-1.28) | 1.21 (1.02-1.44) |  | 0.0321 |
| Other cardiac disease |  |  |  |  |  |  |  |
| Events | 466 | 480 | 505 | 486 | 612 |  |  |
| Person-years | 125144 | 124540 | 123554 | 122450 | 118739 |  |  |
| HR (95% CI), Model 1 | Reference | 1.04 (0.91-1.18) | 1.09 (0.96-1.24) | 1.07 (0.94-1.21) | 1.39 (1.23-1.57) |  | <0.0001 |
| HR (95% CI), Model 2 | Reference | 1.10 (0.96-1.24) | 1.20 (1.05-1.36) | 1.19 (1.05-1.35) | 1.57 (1.39-1.78) |  | <0.0001 |
| HR (95% CI), Model 3 | Reference | 1.05 (0.93-1.20) | 1.12 (0.98-1.27) | 1.07 (0.94-1.21) | 1.30 (1.15-1.47) |  | 0.0002 |
| Stroke |  |  |  |  |  |  |  |
| Events | 115 | 124 | 141 | 118 | 143 |  |  |
| Person-years | 125253 | 124695 | 123999 | 122797 | 118837 |  |  |
| HR (95% CI), Model 1 | Reference | 1.08 (0.83-1.39) | 1.25 (0.98-1.61) | 1.06 (0.82-1.37) | 1.33 (1.04-1.69) |  | 0.0696 |
| HR (95% CI), Model 2 | Reference | 1.14 (0.89-1.48) | 1.33 (1.04-1.71) | 1.16 (0.90-1.50) | 1.49 (1.16-1.90) |  | <0.0001 |
| HR (95% CI), Model 3 | Reference | 1.12 (0.87-1.45) | 1.29 (1.01-1.65) | 1.10 (0.85-1.43) | 1.34 (1.04-1.73) |  | 0.0482 |
| Peripheral vascular disease |  |  |  |  |  |  |  |
| Events | 128 | 120 | 120 | 127 | 144 |  |  |
| Person-years | 126736 | 126751 | 125847 | 124723 | 121286 |  |  |
| HR (95% CI), Model 1 | Reference | 0.93 (0.72-1.19) | 0.94 (0.73-1.20) | 1.00 (0.78-1.28) | 1.15 (0.91-1.46) |  | 0.1702 |
| HR (95% CI), Model 2 | Reference | 0.97 (0.75-1.24) | 1.01 (0.78-1.29) | 1.08 (0.85-1.39) | 1.25 (0.98-1.59) |  | 0.0420 |
| HR (95% CI), Model 3 | Reference | 0.95 (0.74-1.22) | 0.97 (0.76-1.25) | 1.01 (0.79-1.29) | 1.09 (0.85-1.40) |  | 0.3979 |
| Hypertension |  |  |  |  |  |  |  |
| Events | 534 | 554 | 538 | 547 | 535 |  |  |
| Person-years | 96732 | 94103 | 90832 | 87180 | 78828 |  |  |
| HR (95% CI), Model 1 | Reference | 1.06 (0.94-1.20) | 1.08 (0.95-1.21) | 1.13 (1.00-1.27) | 1.23 (1.09-1.38) |  | 0.0008 |
| HR (95% CI), Model 2 | Reference | 1.12 (0.99-1.26) | 1.16 (1.03-1.31) | 1.27 (1.12-1.43) | 1.41 (1.25-1.59) |  | <0.0001 |
| HR (95% CI), Model 3 | Reference | 1.07 (0.95-1.19) | 1.06 (0.95-1.19) | 1.12 (1.00-1.25) | 1.13 (1.01-1.27) |  | 0.1068 |
| Diabetes |  |  |  |  |  |  |  |
| Events | 263 | 301 | 340 | 404 | 477 |  |  |
| Person-years | 123234 | 122442 | 120736 | 118137 | 109292 |  |  |
| HR (95% CI), Model 1 | Reference | 1.16 (0.98-1.37) | 1.32 (1.13-1.55) | 1.61 (1.38-1.88) | 2.04 (1.76-2.38) |  | <0.0001 |
| HR (95% CI), Model 2 | Reference | 1.21 (1.02-1.42) | 1.43 (1.22-1.68) | 1.78 (1.52-2.08) | 2.31 (1.98-2.69) |  | <0.0001 |
| HR (95% CI), Model 3 | Reference | 1.10 (0.93-1.30) | 1.19 (1.01-1.40) | 1.39 (1.19-1.63) | 1.55 (1.32-1.81) |  | <0.0001 |
| Dyslipidemia |  |  |  |  |  |  |  |
| Events | 663 | 680 | 766 | 778 | 850 |  |  |
| Person-years | 112283 | 109578 | 105998 | 103763 | 96044 |  |  |
| HR (95% CI), Model 1 | Reference | 1.04 (0.94-1.16) | 1.22 (1.10-1.35) | 1.27 (1.14-1.41) | 1.50 (1.36-1.66) |  | <0.0001 |
| HR (95% CI), Model 2 | Reference | 1.11 (1.00-1.23) | 1.35 (1.22-1.49) | 1.41 (1.27-1.56) | 1.74 (1.58-1.93) |  | <0.0001 |
| HR (95% CI), Model 3 | Reference | 1.04 (0.94-1.16) | 1.22 (1.10-1.35) | 1.19 (1.07-1.32) | 1.30 (1.17-1.44) |  | <0.0001 |
| Non-melanoma skin cancer |  |  |  |  |  |  |  |
| Events | 373 | 399 | 407 | 349 | 383 |  |  |
| Person-years | 128698 | 128866 | 128787 | 128890 | 128596 |  |  |
| HR (95% CI), Model 1 | Reference | 1.07 (0.93-1.23) | 1.09 (0.95-1.26) | 0.93 (0.81-1.08) | 1.03 (0.89-1.19) |  | 0.53 |
| HR (95% CI), Model 2 | Reference | 1.08 (0.94-1.25) | 1.12 (0.97-1.29) | 0.97 (0.84-1.12) | 1.09 (0.94-1.26) |  | 0.48 |
| HR (95% CI), Model 3 | Reference | 1.10 (0.95-1.26) | 1.15 (1.00-1.32) | 1.00 (0.87-1.16) | 1.16 (1.00-1.34) |  | 0.18 |
| Melanoma |  |  |  |  |  |  |  |
| Events | 48 | 79 | 43 | 58 | 65 |  |  |
| Person-years | 130329 | 130551 | 131054 | 130909 | 130791 |  |  |
| HR (95% CI), Model 1 | Reference | 1.64 (1.15-2.35) | 0.89 (0.59-1.34) | 1.20 (0.82-1.76) | 1.35 (0.93-1.96) |  | 0.47 |
| HR (95% CI), Model 2 | Reference | 1.65 (1.15-2.37) | 0.89 (0.59-1.34) | 1.21 (0.83-1.78) | 1.38 (0.95-2.01) |  | 0.39 |
| HR (95% CI), Model 3 | Reference | 1.66 (1.16-2.37) | 0.90 (0.59-1.36) | 1.24 (0.84-1.82) | 1.44 (0.98-2.11) |  | 0.30 |
| Lung cancer |  |  |  |  |  |  |  |
| Events | 95 | 107 | 100 | 95 | 109 |  |  |
| Person-years | 131250 | 131370 | 131556 | 131588 | 131377 |  |  |
| HR (95% CI), Model 1 | Reference | 1.11 (0.84-1.47) | 1.04 (0.78-1.38) | 0.98 (0.74-1.31) | 1.14 (0.87-1.51) |  | 0.87 |
| HR (95% CI), Model 2 | Reference | 1.15 (0.87-1.52) | 1.10 (0.83-1.45) | 1.03 (0.77-1.37) | 1.16 (0.88-1.53) |  | 0.59 |
| HR (95% CI), Model 3 | Reference | 1.16 (0.88-1.53) | 1.10 (0.83-1.46) | 1.03 (0.77-1.37) | 1.14 (0.86-1.52) |  | 0.62 |
| Stomach Cancer |  |  |  |  |  |  |  |
| Events | 28 | 30 | 21 | 20 | 25 |  |  |
| Person-years | 131712 | 131914 | 132120 | 132134 | 131919 |  |  |
| HR (95% CI), Model 1 | Reference | 1.07 (0.64-1.79) | 0.75 (0.42-1.31) | 0.71 (0.40-1.26) | 0.89 (0.52-1.53) |  | 0.13 |
| HR (95% CI), Model 2 | Reference | 1.14 (0.68-1.91) | 0.83 (0.47-1.46) | 0.82 (0.46-1.47) | 1.06 (0.62-1.83) |  | 0.49 |
| HR (95% CI), Model 3 | Reference | 1.12 (0.67-1.88) | 0.79 (0.44-1.39) | 0.77 (0.43-1.37) | 0.93 (0.53-1.63) |  | 0.31 |
| Colon cancer |  |  |  |  |  |  |  |
| Events | 110 | 90 | 108 | 77 | 99 |  |  |
| Person-years | 130691 | 131138 | 131017 | 131191 | 130978 |  |  |
| HR (95% CI), Model 1 | Reference | 0.82 (0.62-1.09) | 0.97 (0.74-1.27) | 0.70 (0.52-0.94) | 0.90 (0.68-1.18) |  | 0.0618 |
| HR (95% CI), Model 2 | Reference | 0.83 (0.63-1.10) | 1.01 (0.78-1.32) | 0.73 (0.54-0.98) | 0.94 (0.72-1.24) |  | 0.0981 |
| HR (95% CI), Model 3 | Reference | 0.82 (0.62-1.08) | 0.98 (0.75-1.28) | 0.69 (0.51-0.93) | 0.86 (0.65-1.13) |  | 0.22 |
| Oesophageal cancer |  |  |  |  |  |  |  |
| Events | 21 | 30 | 34 | 30 | 40 |  |  |
| Person-years | 131753 | 131888 | 132047 | 132040 | 131905 |  |  |
| HR (95% CI), Model 1 | Reference | 1.42 (0.82-2.49) | 1.61 (0.93-2.77) | 1.42 (0.81-2.48) | 1.90 (1.12-3.22) |  | 0.0646 |
| HR (95% CI), Model 2 | Reference | 1.49 (0.85-2.61) | 1.76 (1.02-3.03) | 1.60 (0.92-2.80) | 2.18 (1.28-3.71) |  | 0.0077 |
| HR (95% CI), Model 3 | Reference | 1.50 (0.86-2.62) | 1.77 (1.02-3.05) | 1.63 (0.93-2.85) | 2.25 (1.31-3.87) |  | 0.0062 |
| Rectal cancer |  |  |  |  |  |  |  |
| Events | 31 | 37 | 43 | 46 | 41 |  |  |
| Person-years | 131460 | 131636 | 131805 | 131807 | 131570 |  |  |
| HR (95% CI), Model 1 | Reference | 1.19 (0.74-1.92) | 1.39 (0.87-2.20) | 1.45 (0.92-2.29) | 1.33 (0.83-2.12) |  | 0.14 |
| HR (95% CI), Model 2 | Reference | 1.26 (0.78-2.02) | 1.49 (0.94-2.36) | 1.65 (1.04-2.61) | 1.52 (0.95-2.43) |  | 0.29 |
| HR (95% CI), Model 3 | Reference | 1.25 (0.78-2.02) | 1.48 (0.93-2.35) | 1.62 (1.02-2.57) | 1.44 (0.89-2.34) |  | 0.0664 |
| Prostate cancer^‡^ |  |  |  |  |  |  |  |
| Events | 255 | 230 | 229 | 171 | 160 |  |  |
| Person-years | 70051 | 62716 | 58996 | 54300 | 47608 |  |  |
| HR (95% CI), Model 1 | Reference | 1.01 (0.84-1.20) | 1.07 (0.89-1.27) | 0.85 (0.70-1.03) | 0.92 (0.75-1.12) |  | 0.19 |
| HR (95% CI), Model 2 | Reference | 1.02 (0.86-1.22) | 1.09 (0.91-1.31) | 0.88 (0.73-1.07) | 0.93 (0.76-1.14) |  | 0.25 |
| HR (95% CI), Model 3 | Reference | 1.03 (0.86-1.23) | 1.09 (0.91-1.31) | 0.89 (0.73-1.08) | 0.95 (0.78-1.17) |  | 0.32 |
| Ovarian cancer^¶^ |  |  |  |  |  |  |  |
| Events | 24 | 19 | 33 | 26 | 28 |  |  |
| Person-years | 59187 | 66909 | 70840 | 75945 | 82632 |  |  |
| HR (95% CI), Model 1 | Reference | 0.70 (0.38-1.28) | 1.15 (0.68-1.94) | 0.84 (0.48-1.47) | 0.81 (0.46-1.40) |  | 0.43 |
| HR (95% CI), Model 2 | Reference | 0.70 (0.38-1.28) | 1.17 (0.69-1.98) | 0.85 (0.49-1.49) | 0.83 (0.48-1.43) |  | 0.42 |
| HR (95% CI), Model 3 | Reference | 0.70 (0.38-1.27) | 1.15 (0.67-1.95) | 0.83 (0.47-1.45) | 0.79 (0.45-1.38) |  | 0.40 |
| Breast cancer^¶^ |  |  |  |  |  |  |  |
| Events | 151 | 177 | 199 | 206 | 208 |  |  |
| Person-years | 56445 | 63202 | 67039 | 71850 | 77944 |  |  |
| HR (95% CI), Model 1 | Reference | 1.04 (0.84-1.30) | 1.11 (0.90-1.37) | 1.07 (0.87-1.32) | 0.99 (0.81-1.23) |  | 0.80 |
| HR (95% CI), Model 2 | Reference | 1.04 (0.84-1.29) | 1.10 (0.89-1.36) | 1.06 (0.86-1.31) | 0.99 (0.81-1.23) |  | 0.83 |
| HR (95% CI), Model 3 | Reference | 1.03 (0.83-1.28) | 1.08 (0.87-1.33) | 1.03 (0.84-1.28) | 0.95 (0.77-1.18) |  | 0.80 |
| Other cancer |  |  |  |  |  |  |  |
| Events | 591 | 611 | 627 | 634 | 682 |  |  |
| Person-years | 122703 | 122292 | 121992 | 122254 | 120408 |  |  |
| HR (95% CI), Model 1 | Reference | 1.04 (0.92-1.16) | 1.06 (0.95-1.19) | 1.07 (0.96-1.20) | 1.18 (1.05-1.32) |  | 0.0208 |
| HR (95% CI), Model 2 | Reference | 1.04 (0.93-1.17) | 1.08 (0.97-1.21) | 1.09 (0.97-1.22) | 1.19 (1.07-1.33) |  | 0.0042 |
| HR (95% CI), Model 3 | Reference | 1.03 (0.92-1.15) | 1.05 (0.94-1.18) | 1.05 (0.94-1.17) | 1.12 (1.00-1.25) |  | 0.0878 |
| Depression |  |  |  |  |  |  |  |
| Events | 155 | 162 | 164 | 180 | 195 |  |  |
| Person-years | 119976 | 119211 | 118473 | 116979 | 112123 |  |  |
| HR (95% CI), Model 1 | Reference | 1.04 (0.83-1.30) | 1.05 (0.85-1.31) | 1.18 (0.95-1.46) | 1.32 (1.07-1.63) |  | 0.0036 |
| HR (95% CI), Model 2 | Reference | 1.01 (0.81-1.25) | 1.03 (0.83-1.29) | 1.12 (0.90-1.39) | 1.20 (0.97-1.48) |  | 0.0546 |
| HR (95% CI), Model 3 | Reference | 0.97 (0.78-1.21) | 0.97 (0.78-1.21) | 1.01 (0.81-1.26) | 1.01 (0.81-1.25) |  | 0.81 |
| Anxiety |  |  |  |  |  |  |  |
| Events | 357 | 380 | 335 | 377 | 438 |  |  |
| Person-years | 123873 | 123478 | 122935 | 121734 | 117828 |  |  |
| HR (95% CI), Model 1 | Reference | 1.06 (0.92-1.23) | 0.94 (0.81-1.09) | 1.06 (0.92-1.23) | 1.28 (1.11-1.48) |  | 0.0019 |
| HR (95% CI), Model 2 | Reference | 1.01 (0.88-1.17) | 0.89 (0.77-1.04) | 0.99 (0.85-1.14) | 1.12 (0.97-1.29) |  | 0.19 |
| HR (95% CI), Model 3 | Reference | 0.99 (0.86-1.15) | 0.86 (0.74-1.00) | 0.93 (0.81-1.08) | 1.01 (0.87-1.17) |  | 0.89 |
| Schizophrenia |  |  |  |  |  |  |  |
| Events | 18 | 21 | 15 | 23 | 31 |  |  |
| Person-years | 127183 | 127046 | 126253 | 125262 | 121853 |  |  |
| HR (95% CI), Model 1 | Reference | 1.17 (0.62-2.19) | 0.84 (0.42-1.67) | 1.30 (0.70-2.40) | 1.74 (0.97-3.12) |  | 0.0409 |
| HR (95% CI), Model 2 | Reference | 1.16 (0.62-2.19) | 0.85 (0.43-1.68) | 1.31 (0.70-2.43) | 1.69 (0.94-3.05) |  | 0.0636 |
| HR (95% CI), Model 3 | Reference | 1.11 (0.59-2.09) | 0.77 (0.39-1.53) | 1.15 (0.62-2.16) | 1.34 (0.73-2.45) |  | 0.33 |
| Alcohol problems |  |  |  |  |  |  |  |
| Events | 104 | 95 | 95 | 110 | 135 |  |  |
| Person-years | 126761 | 126722 | 125905 | 124919 | 121486 |  |  |
| HR (95% CI), Model 1 | Reference | 0.92 (0.70-1.22) | 0.93 (0.70-1.23) | 1.08 (0.83-1.42) | 1.36 (1.05-1.75) |  | 0.0064 |
| HR (95% CI), Model 2 | Reference | 0.97 (0.74-1.28) | 1.04 (0.78-1.37) | 1.22 (0.93-1.60) | 1.62 (1.25-2.10) |  | 0.0001 |
| HR (95% CI), Model 3 | Reference | 0.95 (0.72-1.26) | 0.99 (0.75-1.32) | 1.15 (0.87-1.50) | 1.46 (1.12-1.90) |  | 0.0020 |
| Psychoactive substance abuse |  |  |  |  |  |  |  |
| Events | 34 | 30 | 29 | 25 | 43 |  |  |
| Person-years | 127465 | 127324 | 126507 | 125557 | 122198 |  |  |
| HR (95% CI), Model 1 | Reference | 0.88 (0.54-1.44) | 0.86 (0.52-1.41) | 0.72 (0.43-1.21) | 1.29 (0.82-2.03) |  | 0.33 |
| HR (95% CI), Model 2 | Reference | 0.96 (0.58-1.56) | 0.96 (0.58-1.58) | 0.85 (0.51-1.43) | 1.55 (0.98-2.44) |  | 0.12 |
| HR (95% CI), Model 3 | Reference | 0.96 (0.59-1.57) | 0.98 (0.59-1.61) | 0.85 (0.51-1.44) | 1.53 (0.96-2.45) |  | 0.15 |
| Dementia |  |  |  |  |  |  |  |
| Events | 133 | 136 | 129 | 134 | 142 |  |  |
| Person-years | 127439 | 127131 | 126307 | 125522 | 122226 |  |  |
| HR (95% CI), Model 1 | Reference | 1.01 (0.79-1.28) | 0.97 (0.76-1.24) | 1.02 (0.80-1.29) | 1.10 (0.87-1.40) |  | 0.44 |
| HR (95% CI), Model 2 | Reference | 1.07 (0.84-1.36) | 1.05 (0.82-1.34) | 1.14 (0.90-1.46) | 1.23 (0.96-1.56) |  | 0.0868 |
| HR (95% CI), Model 3 | Reference | 1.06 (0.83-1.34) | 1.01 (0.79-1.29) | 1.07 (0.84-1.37) | 1.07 (0.84-1.37) |  | 0.59 |
| Parkinson's disease |  |  |  |  |  |  |  |
| Events | 65 | 59 | 48 | 64 | 49 |  |  |
| Person-years | 127394 | 127255 | 126444 | 125578 | 122464 |  |  |
| HR (95% CI), Model 1 | Reference | 0.91 (0.64-1.29) | 0.74 (0.51-1.08) | 1.00 (0.71-1.41) | 0.78 (0.54-1.14) |  | 0.40 |
| HR (95% CI), Model 2 | Reference | 0.96 (0.68-1.37) | 0.81 (0.56-1.18) | 1.13 (0.80-1.60) | 0.92 (0.63-1.33) |  | 0.99 |
| HR (95% CI), Model 3 | Reference | 0.96 (0.67-1.36) | 0.80 (0.55-1.17) | 1.10 (0.77-1.56) | 0.86 (0.59-1.27) |  | 0.76 |
| Multiple sclerosis |  |  |  |  |  |  |  |
| Events | 7 | 10 | 9 | 6 | 11 |  |  |
| Person-years | 127555 | 127173 | 126408 | 125427 | 122270 |  |  |
| HR (95% CI), Model 1 | Reference | 1.43 (0.54-3.76) | 1.15 (0.42-3.18) | 0.87 (0.29-2.59) | 1.64 (0.63-4.22) |  | 0.60 |
| HR (95% CI), Model 2 | Reference | 1.64 (0.65-4.18) | 1.31 (0.50-3.46) | 0.78 (0.26-2.34) | 1.34 (0.51-3.52) |  | 0.72 |
| HR (95% CI), Model 3 | Reference | 1.37 (0.52-3.62) | 1.24 (0.46-3.35) | 0.81 (0.27-2.43) | 1.35 (0.50-3.60) |  | 0.92 |
| Migraine |  |  |  |  |  |  |  |
| Events | 67 | 59 | 73 | 77 | 77 |  |  |
| Person-years | 123608 | 123535 | 123091 | 121414 | 118082 |  |  |
| HR (95% CI), Model 1 | Reference | 0.88 (0.62-1.25) | 1.09 (0.78-1.52) | 1.17 (0.84-1.62) | 1.18 (0.85-1.65) |  | 0.0828 |
| HR (95% CI), Model 2 | Reference | 0.83 (0.59-1.18) | 1.01 (0.73-1.41) | 1.05 (0.76-1.46) | 1.02 (0.73-1.42) |  | 0.47 |
| HR (95% CI), Model 3 | Reference | 0.82 (0.57-1.16) | 0.98 (0.70-1.36) | 0.99 (0.71-1.39) | 0.93 (0.66-1.30) |  | 0.91 |
| Epilepsy |  |  |  |  |  |  |  |
| Events | 60 | 50 | 62 | 53 | 68 |  |  |
| Person-years | 126121 | 125950 | 125606 | 124587 | 121380 |  |  |
| HR (95% CI), Model 1 | Reference | 0.82 (0.56-1.19) | 1.04 (0.73-1.48) | 0.86 (0.59-1.25) | 1.18 (0.83-1.67) |  | 0.31 |
| HR (95% CI), Model 2 | Reference | 0.86 (0.59-1.25) | 1.08 (0.76-1.55) | 0.95 (0.66-1.38) | 1.26 (0.88-1.79) |  | 0.17 |
| HR (95% CI), Model 3 | Reference | 0.84 (0.58-1.23) | 1.05 (0.74-1.50) | 0.90 (0.62-1.32) | 1.15 (0.80-1.64) |  | 0.42 |
| COPD |  |  |  |  |  |  |  |
| Events | 281 | 292 | 274 | 310 | 337 |  |  |
| Person-years | 125130 | 124408 | 123626 | 122362 | 118677 |  |  |
| HR (95% CI), Model 1 | Reference | 1.05 (0.89-1.24) | 0.99 (0.84-1.17) | 1.13 (0.96-1.33) | 1.25 (1.06-1.46) |  | 0.0050 |
| HR (95% CI), Model 2 | Reference | 1.11 (0.94-1.31) | 1.09 (0.92-1.29) | 1.25 (1.06-1.47) | 1.38 (1.18-1.62) |  | <0.0001 |
| HR (95% CI), Model 3 | Reference | 1.09 (0.92-1.28) | 1.05 (0.89-1.24) | 1.18 (1.00-1.39) | 1.24 (1.05-1.46) |  | 0.0066 |
| Asthma |  |  |  |  |  |  |  |
| Events | 197 | 211 | 192 | 202 | 254 |  |  |
| Person-years | 112174 | 112202 | 111885 | 109531 | 105020 |  |  |
| HR (95% CI), Model 1 | Reference | 1.07 (0.88-1.30) | 0.97 (0.79-1.18) | 1.04 (0.86-1.27) | 1.37 (1.14-1.65) |  | 0.0030 |
| HR (95% CI), Model 2 | Reference | 1.06 (0.87-1.29) | 0.97 (0.80-1.19) | 1.03 (0.85-1.26) | 1.32 (1.10-1.60) |  | 0.0105 |
| HR (95% CI), Model 3 | Reference | 1.03 (0.85-1.25) | 0.92 (0.76-1.13) | 0.96 (0.78-1.17) | 1.16 (0.95-1.40) |  | 0.29 |
| Bronchiectasis |  |  |  |  |  |  |  |
| Events | 94 | 68 | 80 | 75 | 85 |  |  |
| Person-years | 127266 | 127012 | 126195 | 125315 | 122080 |  |  |
| HR (95% CI), Model 1 | Reference | 0.72 (0.53-0.99) | 0.85 (0.63-1.14) | 0.80 (0.59-1.08) | 0.92 (0.68-1.24) |  | 0.90 |
| HR (95% CI), Model 2 | Reference | 0.72 (0.53-0.98) | 0.87 (0.64-1.17) | 0.81 (0.60-1.10) | 0.93 (0.69-1.26) |  | 0.88 |
| HR (95% CI), Model 3 | Reference | 0.73 (0.54-1.00) | 0.89 (0.66-1.21) | 0.85 (0.62-1.15) | 0.99 (0.73-1.34) |  | 0.82 |
| Dyspepsia |  |  |  |  |  |  |  |
| Events | 1191 | 1190 | 1174 | 1136 | 1272 |  |  |
| Person-years | 107482 | 106585 | 106485 | 106100 | 100600 |  |  |
| HR (95% CI), Model 1 | Reference | 1.01 (0.93-1.09) | 0.99 (0.92-1.08) | 0.96 (0.89-1.05) | 1.14 (1.06-1.24) |  | 0.0319 |
| HR (95% CI), Model 2 | Reference | 1.00 (0.93-1.09) | 0.99 (0.92-1.08) | 0.96 (0.88-1.04) | 1.11 (1.03-1.21) |  | 0.0603 |
| HR (95% CI), Model 3 | Reference | 0.98 (0.91-1.07) | 0.96 (0.88-1.04) | 0.91 (0.84-0.99) | 1.02 (0.94-1.10) |  | 0.64 |
| Constipation |  |  |  |  |  |  |  |
| Events | 443 | 388 | 377 | 433 | 453 |  |  |
| Person-years | 124576 | 124864 | 123651 | 122978 | 119027 |  |  |
| HR (95% CI), Model 1 | Reference | 0.88 (0.77-1.01) | 0.86 (0.75-0.98) | 0.99 (0.87-1.13) | 1.07 (0.94-1.22) |  | 0.13 |
| HR (95% CI), Model 2 | Reference | 0.88 (0.76-1.00) | 0.87 (0.76-1.00) | 1.01 (0.88-1.15) | 1.07 (0.94-1.22) |  | 0.0753 |
| HR (95% CI), Model 3 | Reference | 0.86 (0.75-0.99) | 0.84 (0.73-0.97) | 0.96 (0.84-1.10) | 0.98 (0.86-1.13) |  | 0.64 |
| Diverticulitis |  |  |  |  |  |  |  |
| Events | 883 | 892 | 932 | 972 | 977 |  |  |
| Person-years | 120098 | 119580 | 118476 | 117396 | 113960 |  |  |
| HR (95% CI), Model 1 | Reference | 1.02 (0.93-1.11) | 1.07 (0.97-1.17) | 1.13 (1.03-1.23) | 1.17 (1.06-1.28) |  | 0.0002 |
| HR (95% CI), Model 2 | Reference | 1.02 (0.93-1.12) | 1.08 (0.98-1.18) | 1.14 (1.04-1.25) | 1.17 (1.07-1.29) |  | 0.0000 |
| HR (95% CI), Model 3 | Reference | 1.00 (0.91-1.10) | 1.04 (0.95-1.14) | 1.08 (0.98-1.18) | 1.07 (0.97-1.17) |  | 0.0468 |
| Irritable bowel syndrome |  |  |  |  |  |  |  |
| Events | 132 | 104 | 141 | 122 | 160 |  |  |
| Person-years | 124223 | 123570 | 122465 | 121799 | 118212 |  |  |
| HR (95% CI), Model 1 | Reference | 0.79 (0.61-1.02) | 1.08 (0.86-1.37) | 0.94 (0.73-1.20) | 1.27 (1.01-1.61) |  | 0.0252 |
| HR (95% CI), Model 2 | Reference | 0.74 (0.57-0.96) | 0.99 (0.78-1.25) | 0.83 (0.65-1.07) | 1.04 (0.83-1.31) |  | 0.43 |
| HR (95% CI), Model 3 | Reference | 0.73 (0.57-0.95) | 0.97 (0.76-1.23) | 0.81 (0.63-1.03) | 0.98 (0.77-1.24) |  | 0.84 |
| Inflammatory bowel disease |  |  |  |  |  |  |  |
| Events | 73 | 51 | 52 | 62 | 62 |  |  |
| Person-years | 126377 | 126062 | 125285 | 124178 | 121042 |  |  |
| HR (95% CI), Model 1 | Reference | 0.70 (0.49-1.00) | 0.72 (0.50-1.02) | 0.86 (0.62-1.21) | 0.89 (0.63-1.24) |  | 0.82 |
| HR (95% CI), Model 2 | Reference | 0.72 (0.50-1.02) | 0.75 (0.53-1.07) | 0.92 (0.65-1.29) | 0.95 (0.67-1.33) |  | 0.83 |
| HR (95% CI), Model 3 | Reference | 0.72 (0.50-1.03) | 0.76 (0.53-1.08) | 0.93 (0.66-1.31) | 0.96 (0.68-1.36) |  | 0.78 |
| Chronic liver disease |  |  |  |  |  |  |  |
| Events | 29 | 23 | 32 | 38 | 55 |  |  |
| Person-years | 126749 | 126649 | 125820 | 124666 | 121446 |  |  |
| HR (95% CI), Model 1 | Reference | 0.82 (0.47-1.42) | 1.11 (0.67-1.85) | 1.37 (0.84-2.24) | 2.01 (1.27-3.17) |  | 0.0002 |
| HR (95% CI), Model 2 | Reference | 0.83 (0.48-1.44) | 1.20 (0.73-1.99) | 1.48 (0.91-2.40) | 2.23 (1.41-3.51) |  | 0.0000 |
| HR (95% CI), Model 3 | Reference | 0.82 (0.47-1.42) | 1.15 (0.69-1.91) | 1.37 (0.84-2.24) | 1.89 (1.18-3.02) |  | 0.0008 |
| CKD |  |  |  |  |  |  |  |
| Events | 481 | 476 | 547 | 604 | 761 |  |  |
| Person-years | 125727 | 125421 | 124281 | 123042 | 118326 |  |  |
| HR (95% CI), Model 1 | Reference | 1.00 (0.88-1.13) | 1.14 (1.01-1.29) | 1.29 (1.14-1.45) | 1.69 (1.51-1.89) |  | <0.0001 |
| HR (95% CI), Model 2 | Reference | 1.03 (0.91-1.17) | 1.24 (1.10-1.40) | 1.42 (1.26-1.60) | 1.89 (1.68-2.12) |  | <0.0001 |
| HR (95% CI), Model 3 | Reference | 0.98 (0.86-1.11) | 1.12 (0.99-1.27) | 1.20 (1.06-1.35) | 1.42 (1.26-1.60) |  | <0.0001 |
| Osteoporosis |  |  |  |  |  |  |  |
| Events | 739 | 766 | 781 | 793 | 850 |  |  |
| Person-years | 115889 | 115483 | 114706 | 114517 | 112917 |  |  |
| HR (95% CI), Model 1 | Reference | 1.04 (0.94-1.15) | 1.07 (0.97-1.18) | 1.08 (0.98-1.20) | 1.18 (1.07-1.30) |  | 0.0024 |
| HR (95% CI), Model 2 | Reference | 1.03 (0.93-1.14) | 1.06 (0.96-1.17) | 1.06 (0.96-1.18) | 1.14 (1.03-1.25) |  | 0.0048 |
| HR (95% CI), Model 3 | Reference | 0.99 (0.89-1.09) | 0.97 (0.88-1.08) | 0.95 (0.86-1.05) | 0.95 (0.86-1.05) |  | 0.48 |
| Fracture |  |  |  |  |  |  |  |
| Events | 115 | 101 | 106 | 107 | 113 |  |  |
| Person-years | 130956 | 131313 | 131240 | 131500 | 131199 |  |  |
| HR (95% CI), Model 1 | Reference | 0.88 (0.67-1.14) | 0.91 (0.70-1.19) | 0.93 (0.71-1.20) | 0.97 (0.75-1.26) |  | 0.99 |
| HR (95% CI), Model 2 | Reference | 0.85 (0.65-1.11) | 0.88 (0.68-1.15) | 0.88 (0.67-1.14) | 0.89 (0.69-1.16) |  | 0.55 |
| HR (95% CI), Model 3 | Reference | 0.87 (0.66-1.14) | 0.92 (0.71-1.20) | 0.92 (0.70-1.20) | 0.95 (0.73-1.24) |  | 0.84 |
| Glaucoma |  |  |  |  |  |  |  |
| Events | 191 | 177 | 176 | 193 | 199 |  |  |
| Person-years | 129393 | 130105 | 129791 | 129894 | 129588 |  |  |
| HR (95% CI), Model 1 | Reference | 0.91 (0.74-1.12) | 0.91 (0.74-1.11) | 1.01 (0.83-1.24) | 1.04 (0.85-1.27) |  | 0.66 |
| HR (95% CI), Model 2 | Reference | 0.93 (0.75-1.14) | 0.93 (0.75-1.14) | 1.02 (0.84-1.25) | 1.04 (0.85-1.27) |  | 0.48 |
| HR (95% CI), Model 3 | Reference | 0.92 (0.75-1.13) | 0.92 (0.75-1.13) | 1.01 (0.83-1.24) | 1.00 (0.82-1.23) |  | 0.67 |
| Cataract |  |  |  |  |  |  |  |
| Events | 893 | 913 | 945 | 919 | 977 |  |  |
| Person-years | 124610 | 124803 | 124977 | 124639 | 123366 |  |  |
| HR (95% CI), Model 1 | Reference | 1.02 (0.93-1.12) | 1.05 (0.96-1.15) | 1.03 (0.94-1.13) | 1.11 (1.01-1.21) |  | 0.61 |
| HR (95% CI), Model 2 | Reference | 1.02 (0.93-1.12) | 1.06 (0.97-1.16) | 1.03 (0.94-1.13) | 1.08 (0.98-1.18) |  | 0.72 |
| HR (95% CI), Model 3 | Reference | 1.00 (0.91-1.10) | 1.02 (0.93-1.12) | 0.97 (0.89-1.07) | 0.97 (0.89-1.07) |  | 0.11 |
| AMD |  |  |  |  |  |  |  |
| Events | 139 | 132 | 137 | 138 | 188 |  |  |
| Person-years | 131037 | 131336 | 131504 | 131353 | 131022 |  |  |
| HR (95% CI), Model 1 | Reference | 0.95 (0.75-1.20) | 0.97 (0.77-1.23) | 0.99 (0.78-1.25) | 1.34 (1.07-1.67) |  | 0.0202 |
| HR (95% CI), Model 2 | Reference | 0.94 (0.74-1.20) | 0.97 (0.77-1.23) | 0.97 (0.77-1.23) | 1.30 (1.04-1.62) |  | 0.0258 |
| HR (95% CI), Model 3 | Reference | 0.93 (0.73-1.18) | 0.95 (0.75-1.21) | 0.94 (0.74-1.19) | 1.22 (0.97-1.53) |  | 0.0930 |
| Pernicious anaemia |  |  |  |  |  |  |  |
| Events | 21 | 22 | 25 | 15 | 21 |  |  |
| Person-years | 127365 | 127186 | 126391 | 125545 | 122154 |  |  |
| HR (95% CI), Model 1 | Reference | 1.05 (0.58-1.91) | 1.15 (0.64-2.07) | 0.72 (0.37-1.40) | 1.04 (0.57-1.91) |  | 0.69 |
| HR (95% CI), Model 2 | Reference | 1.03 (0.57-1.88) | 1.20 (0.67-2.15) | 0.72 (0.37-1.39) | 1.00 (0.54-1.84) |  | 0.64 |
| HR (95% CI), Model 3 | Reference | 0.99 (0.54-1.81) | 1.11 (0.62-1.99) | 0.62 (0.32-1.22) | 0.76 (0.40-1.44) |  | 0.18 |
| Thyroid disorders |  |  |  |  |  |  |  |
| Events | 204 | 239 | 232 | 264 | 299 |  |  |
| Person-years | 120716 | 119486 | 119244 | 118165 | 115592 |  |  |
| HR (95% CI), Model 1 | Reference | 1.18 (0.98-1.42) | 1.14 (0.95-1.38) | 1.31 (1.09-1.57) | 1.53 (1.28-1.82) |  | <0.0001 |
| HR (95% CI), Model 2 | Reference | 1.11 (0.93-1.33) | 1.05 (0.87-1.26) | 1.18 (0.99-1.41) | 1.27 (1.06-1.51) |  | 0.0094 |
| HR (95% CI), Model 3 | Reference | 1.08 (0.90-1.30) | 1.00 (0.83-1.20) | 1.10 (0.91-1.31) | 1.11 (0.93-1.33) |  | 0.25 |
| Eczema |  |  |  |  |  |  |  |
| Events | 111 | 126 | 137 | 122 | 142 |  |  |
| Person-years | 122639 | 122358 | 121524 | 120391 | 116926 |  |  |
| HR (95% CI), Model 1 | Reference | 1.14 (0.88-1.47) | 1.24 (0.96-1.59) | 1.11 (0.86-1.44) | 1.33 (1.04-1.71) |  | 0.0705 |
| HR (95% CI), Model 2 | Reference | 1.14 (0.88-1.47) | 1.25 (0.97-1.61) | 1.13 (0.88-1.47) | 1.33 (1.03-1.70) |  | 0.0507 |
| HR (95% CI), Model 3 | Reference | 1.11 (0.86-1.43) | 1.19 (0.93-1.53) | 1.05 (0.81-1.36) | 1.17 (0.91-1.51) |  | 0.38 |
| Meniere disease |  |  |  |  |  |  |  |
| Events | 16 | 11 | 20 | 8 | 11 |  |  |
| Person-years | 131367 | 131604 | 131762 | 131669 | 131592 |  |  |
| HR (95% CI), Model 1 | Reference | 0.69 (0.32-1.48) | 1.25 (0.65-2.41) | 0.50 (0.21-1.17) | 0.69 (0.32-1.49) |  | 0.33 |
| HR (95% CI), Model 2 | Reference | 0.68 (0.31-1.46) | 1.24 (0.64-2.40) | 0.49 (0.21-1.16) | 0.66 (0.30-1.43) |  | 0.32 |
| HR (95% CI), Model 3 | Reference | 0.65 (0.30-1.40) | 1.15 (0.59-2.24) | 0.45 (0.19-1.05) | 0.56 (0.25-1.24) |  | 0.13 |
| Prostate disorders^‡^ |  |  |  |  |  |  |  |
| Events | 465 | 424 | 375 | 352 | 296 |  |  |
| Person-years | 65166 | 58786 | 54745 | 50613 | 44211 |  |  |
| HR (95% CI), Model 1 | Reference | 1.01 (0.88-1.15) | 0.96 (0.83-1.10) | 0.97 (0.85-1.12) | 0.94 (0.81-1.08) |  | 0.84 |
| HR (95% CI), Model 2 | Reference | 1.01 (0.89-1.16) | 0.97 (0.84-1.11) | 0.97 (0.84-1.12) | 0.91 (0.78-1.05) |  | 0.62 |
| HR (95% CI), Model 3 | Reference | 1.00 (0.87-1.14) | 0.93 (0.81-1.07) | 0.93 (0.81-1.07) | 0.85 (0.73-0.99) |  | 0.20 |

AMD, age related macular degeneration; CI, confidence interval; CKD, chronic kidney disease; COPD, chronic obstructive pulmonary disease; HR, hazard ratio.

*Age gap was calculated by subtracting chronological age from metabolomic age. Chronological age-adjusted age gap was calculated with use of regression models.

^†^Cox proportional regression models were used to examine the association between chronological age-adjusted age gap and incidence of individual chronic diseases by excluding those developed the disease in the first year of follow-up. Model 1 was unadjusted; Model 2 was adjusted for Model 1 plus age, sex, ethnicity, education, household income, diet score, alcohol consumption, physical activity, smoking, sleep duration, fasting duration, and GRS for longevity; Model 3 was adjusted for Model 2 plus BMI, high cholesterol, hypertension, and antihypertensive and lipid-lowering medications.

^‡^These analyses were conducted among men only.

^¶^These analyses were conducted among women only.

**Table S5. The association between chronological age-adjusted age gap and risk of individual diseases in validation population with follow-up duration of ≥5 years**

|  | Chronological age-adjusted age gap* | | | | |  | P-value |
| --- | --- | --- | --- | --- | --- | --- | --- |
|  | Quintile 1 | Quintile 2 | Quintile 3 | Quintile 4 | Quintile 5 |  | for trend |
| Coronary heart disease |  |  |  |  |  |  |  |
| Events | 457 | 452 | 439 | 472 | 492 |  |  |
| Person-years | 123064 | 122959 | 121550 | 120384 | 116952 |  |  |
| HR (95% CI), Model 1^†^ | Reference | 0.98 (0.86-1.12) | 0.97 (0.85-1.10) | 1.05 (0.92-1.19) | 1.13 (0.99-1.28) |  | 0.0564 |
| HR (95% CI), Model 2 | Reference | 1.05 (0.92-1.20) | 1.06 (0.93-1.21) | 1.19 (1.04-1.35) | 1.31 (1.15-1.48) |  | 0.0000 |
| HR (95% CI), Model 3 | Reference | 1.01 (0.89-1.15) | 0.98 (0.86-1.12) | 1.05 (0.93-1.20) | 1.06 (0.93-1.21) |  | 0.33 |
| Heart failure |  |  |  |  |  |  |  |
| Events | 170 | 162 | 185 | 190 | 248 |  |  |
| Person-years | 126593 | 126510 | 125483 | 124310 | 120523 |  |  |
| HR (95% CI), Model 1 | Reference | 0.96 (0.77-1.19) | 1.10 (0.89-1.35) | 1.14 (0.93-1.41) | 1.53 (1.25-1.86) |  | <0.0001 |
| HR (95% CI), Model 2 | Reference | 1.01 (0.81-1.25) | 1.21 (0.98-1.49) | 1.29 (1.05-1.59) | 1.76 (1.44-2.14) |  | <0.0001 |
| HR (95% CI), Model 3 | Reference | 0.96 (0.77-1.19) | 1.10 (0.89-1.35) | 1.11 (0.90-1.37) | 1.34 (1.09-1.64) |  | 0.0018 |
| Atrial fibrillation |  |  |  |  |  |  |  |
| Events | 149 | 151 | 126 | 140 | 155 |  |  |
| Person-years | 124507 | 124033 | 122854 | 121736 | 117699 |  |  |
| HR (95% CI), Model 1 | Reference | 1.00 (0.80-1.26) | 0.86 (0.68-1.09) | 0.96 (0.76-1.21) | 1.08 (0.86-1.35) |  | 0.58 |
| HR (95% CI), Model 2 | Reference | 1.09 (0.87-1.37) | 0.96 (0.76-1.22) | 1.13 (0.89-1.42) | 1.37 (1.09-1.72) |  | 0.0138 |
| HR (95% CI), Model 3 | Reference | 1.05 (0.84-1.32) | 0.90 (0.71-1.15) | 1.02 (0.81-1.29) | 1.15 (0.91-1.45) |  | 0.37 |
| Other cardiac disease |  |  |  |  |  |  |  |
| Events | 383 | 395 | 401 | 390 | 493 |  |  |
| Person-years | 124872 | 124280 | 123216 | 122124 | 118355 |  |  |
| HR (95% CI), Model 1 | Reference | 1.04 (0.90-1.19) | 1.05 (0.92-1.21) | 1.03 (0.90-1.19) | 1.36 (1.19-1.55) |  | 0.0001 |
| HR (95% CI), Model 2 | Reference | 1.10 (0.95-1.26) | 1.15 (1.00-1.33) | 1.16 (1.01-1.34) | 1.54 (1.35-1.77) |  | <0.0001 |
| HR (95% CI), Model 3 | Reference | 1.06 (0.92-1.22) | 1.08 (0.94-1.24) | 1.04 (0.90-1.20) | 1.28 (1.11-1.47) |  | 0.0022 |
| Stroke |  |  |  |  |  |  |  |
| Events | 73 | 70 | 87 | 59 | 82 |  |  |
| Person-years | 125115 | 124511 | 123822 | 122588 | 118639 |  |  |
| HR (95% CI), Model 1 | Reference | 0.96 (0.69-1.34) | 1.23 (0.90-1.68) | 0.84 (0.60-1.18) | 1.20 (0.88-1.65) |  | 0.58 |
| HR (95% CI), Model 2 | Reference | 1.01 (0.73-1.40) | 1.27 (0.93-1.74) | 0.89 (0.63-1.26) | 1.31 (0.95-1.80) |  | 0.22 |
| HR (95% CI), Model 3 | Reference | 0.98 (0.71-1.37) | 1.22 (0.89-1.67) | 0.83 (0.59-1.18) | 1.15 (0.83-1.60) |  | 0.68 |
| Peripheral vascular disease |  |  |  |  |  |  |  |
| Events | 94 | 95 | 96 | 92 | 100 |  |  |
| Person-years | 126621 | 126671 | 125773 | 124614 | 121138 |  |  |
| HR (95% CI), Model 1 | Reference | 1.00 (0.75-1.33) | 1.03 (0.77-1.36) | 0.99 (0.74-1.32) | 1.09 (0.82-1.44) |  | 0.59 |
| HR (95% CI), Model 2 | Reference | 1.04 (0.78-1.38) | 1.08 (0.82-1.44) | 1.05 (0.79-1.40) | 1.16 (0.87-1.54) |  | 0.34 |
| HR (95% CI), Model 3 | Reference | 1.02 (0.76-1.35) | 1.05 (0.79-1.40) | 0.98 (0.73-1.31) | 1.00 (0.75-1.34) |  | 0.90 |
| Hypertension |  |  |  |  |  |  |  |
| Events | 293 | 300 | 296 | 295 | 291 |  |  |
| Person-years | 95968 | 93270 | 90077 | 86398 | 78038 |  |  |
| HR (95% CI), Model 1 | Reference | 1.04 (0.89-1.23) | 1.08 (0.91-1.26) | 1.12 (0.95-1.32) | 1.22 (1.03-1.43) |  | 0.0178 |
| HR (95% CI), Model 2 | Reference | 1.10 (0.94-1.30) | 1.16 (0.99-1.36) | 1.23 (1.05-1.45) | 1.38 (1.17-1.63) |  | 0.0001 |
| HR (95% CI), Model 3 | Reference | 1.04 (0.89-1.22) | 1.03 (0.88-1.21) | 1.08 (0.93-1.27) | 1.13 (0.97-1.33) |  | 0.11 |
| Diabetes |  |  |  |  |  |  |  |
| Events | 204 | 246 | 263 | 324 | 371 |  |  |
| Person-years | 123051 | 122270 | 120491 | 117881 | 108963 |  |  |
| HR (95% CI), Model 1 | Reference | 1.23 (1.02-1.48) | 1.32 (1.10-1.58) | 1.66 (1.39-1.98) | 2.06 (1.73-2.44) |  | <0.0001 |
| HR (95% CI), Model 2 | Reference | 1.27 (1.06-1.53) | 1.43 (1.19-1.71) | 1.84 (1.54-2.20) | 2.31 (1.94-2.75) |  | <0.0001 |
| HR (95% CI), Model 3 | Reference | 1.16 (0.97-1.40) | 1.19 (0.99-1.43) | 1.45 (1.22-1.73) | 1.58 (1.32-1.88) |  | <0.0001 |
| Dyslipidemia |  |  |  |  |  |  |  |
| Events | 501 | 520 | 552 | 567 | 589 |  |  |
| Person-years | 114901 | 112598 | 109045 | 106704 | 99084 |  |  |
| HR (95% CI), Model 1 | Reference | 1.06 (0.94-1.20) | 1.16 (1.03-1.31) | 1.22 (1.08-1.37) | 1.37 (1.22-1.55) |  | <0.0001 |
| HR (95% CI), Model 2 | Reference | 1.12 (0.99-1.27) | 1.29 (1.14-1.45) | 1.37 (1.22-1.55) | 1.59 (1.40-1.79) |  | <0.0001 |
| HR (95% CI), Model 3 | Reference | 1.09 (0.96-1.24) | 1.21 (1.07-1.37) | 1.23 (1.09-1.40) | 1.24 (1.09-1.41) |  | 0.0002 |
| Non-melanoma skin cancer |  |  |  |  |  |  |  |
| Events | 280 | 297 | 309 | 240 | 279 |  |  |
| Person-years | 128408 | 128528 | 128486 | 128544 | 128282 |  |  |
| HR (95% CI), Model 1 | Reference | 1.06 (0.90-1.24) | 1.11 (0.94-1.30) | 0.86 (0.72-1.02) | 1.00 (0.85-1.18) |  | 0.32 |
| HR (95% CI), Model 2 | Reference | 1.08 (0.92-1.27) | 1.13 (0.96-1.33) | 0.89 (0.75-1.06) | 1.07 (0.90-1.26) |  | 0.76 |
| HR (95% CI), Model 3 | Reference | 1.11 (0.94-1.31) | 1.21 (1.02-1.43) | 0.96 (0.80-1.15) | 1.18 (0.99-1.40) |  | 0.34 |
| Melanoma |  |  |  |  |  |  |  |
| Events | 35 | 47 | 28 | 46 | 47 |  |  |
| Person-years | 130289 | 130443 | 131005 | 130879 | 130736 |  |  |
| HR (95% CI), Model 1 | Reference | 1.34 (0.86-2.07) | 0.79 (0.48-1.30) | 1.30 (0.84-2.02) | 1.34 (0.86-2.07) |  | 0.25 |
| HR (95% CI), Model 2 | Reference | 1.35 (0.87-2.09) | 0.80 (0.49-1.32) | 1.34 (0.86-2.08) | 1.41 (0.91-2.19) |  | 0.16 |
| HR (95% CI), Model 3 | Reference | 1.42 (0.91-2.22) | 0.78 (0.46-1.31) | 1.39 (0.88-2.19) | 1.56 (0.98-2.48) |  | 0.10 |
| Lung cancer |  |  |  |  |  |  |  |
| Events | 63 | 71 | 71 | 63 | 81 |  |  |
| Person-years | 131141 | 131264 | 131472 | 131484 | 131292 |  |  |
| HR (95% CI), Model 1 | Reference | 1.11 (0.79-1.56) | 1.12 (0.80-1.57) | 0.98 (0.69-1.39) | 1.28 (0.92-1.78) |  | 0.81 |
| HR (95% CI), Model 2 | Reference | 1.15 (0.82-1.61) | 1.16 (0.82-1.63) | 1.01 (0.71-1.43) | 1.27 (0.91-1.77) |  | 0.65 |
| HR (95% CI), Model 3 | Reference | 1.18 (0.83-1.68) | 1.06 (0.74-1.52) | 0.94 (0.65-1.37) | 1.20 (0.84-1.72) |  | 0.71 |
| Stomach Cancer |  |  |  |  |  |  |  |
| Events | 20 | 23 | 18 | 18 | 22 |  |  |
| Person-years | 131690 | 131895 | 132109 | 132128 | 131910 |  |  |
| HR (95% CI), Model 1 | Reference | 1.14 (0.63-2.08) | 0.89 (0.47-1.69) | 0.89 (0.47-1.69) | 1.10 (0.60-2.01) |  | 0.60 |
| HR (95% CI), Model 2 | Reference | 1.22 (0.67-2.22) | 0.98 (0.52-1.86) | 1.02 (0.54-1.94) | 1.28 (0.70-2.36) |  | 0.89 |
| HR (95% CI), Model 3 | Reference | 1.27 (0.69-2.34) | 0.96 (0.49-1.85) | 0.94 (0.48-1.84) | 1.08 (0.55-2.12) |  | 0.81 |
| Colon cancer |  |  |  |  |  |  |  |
| Events | 68 | 60 | 71 | 48 | 64 |  |  |
| Person-years | 130563 | 131054 | 130905 | 131100 | 130865 |  |  |
| HR (95% CI), Model 1 | Reference | 0.89 (0.63-1.26) | 1.02 (0.73-1.43) | 0.71 (0.49-1.03) | 0.95 (0.68-1.34) |  | 0.23 |
| HR (95% CI), Model 2 | Reference | 0.90 (0.63-1.27) | 1.07 (0.77-1.50) | 0.73 (0.50-1.06) | 0.98 (0.69-1.38) |  | 0.38 |
| HR (95% CI), Model 3 | Reference | 0.87 (0.61-1.23) | 0.95 (0.67-1.34) | 0.68 (0.46-0.99) | 0.81 (0.56-1.17) |  | 0.12 |
| Oesophageal cancer |  |  |  |  |  |  |  |
| Events | 16 | 20 | 26 | 20 | 31 |  |  |
| Person-years | 131737 | 131856 | 132023 | 132007 | 131878 |  |  |
| HR (95% CI), Model 1 | Reference | 1.25 (0.65-2.40) | 1.61 (0.87-3.01) | 1.24 (0.64-2.39) | 1.93 (1.05-3.53) |  | 0.0890 |
| HR (95% CI), Model 2 | Reference | 1.30 (0.67-2.51) | 1.74 (0.93-3.24) | 1.37 (0.71-2.66) | 2.19 (1.19-4.02) |  | 0.0196 |
| HR (95% CI), Model 3 | Reference | 1.32 (0.69-2.56) | 1.61 (0.85-3.06) | 1.42 (0.73-2.78) | 2.31 (1.23-4.35) |  | 0.0134 |
| Rectal cancer |  |  |  |  |  |  |  |
| Events | 22 | 24 | 29 | 30 | 25 |  |  |
| Person-years | 131433 | 131594 | 131765 | 131762 | 131515 |  |  |
| HR (95% CI), Model 1 | Reference | 1.09 (0.61-1.95) | 1.32 (0.76-2.29) | 1.32 (0.76-2.30) | 1.14 (0.64-2.03) |  | 0.64 |
| HR (95% CI), Model 2 | Reference | 1.15 (0.64-2.05) | 1.44 (0.83-2.51) | 1.54 (0.89-2.67) | 1.34 (0.75-2.38) |  | 0.24 |
| HR (95% CI), Model 3 | Reference | 1.15 (0.63-2.08) | 1.50 (0.85-2.63) | 1.37 (0.77-2.45) | 1.19 (0.64-2.20) |  | 0.43 |
| Prostate cancer^‡^ |  |  |  |  |  |  |  |
| Events | 181 | 167 | 159 | 123 | 124 |  |  |
| Person-years | 69805 | 62526 | 58777 | 54154 | 47489 |  |  |
| HR (95% CI), Model 1 | Reference | 1.03 (0.83-1.27) | 1.04 (0.84-1.29) | 0.86 (0.68-1.08) | 1.00 (0.79-1.25) |  | 0.54 |
| HR (95% CI), Model 2 | Reference | 1.04 (0.84-1.29) | 1.07 (0.86-1.32) | 0.90 (0.71-1.13) | 1.02 (0.81-1.29) |  | 0.66 |
| HR (95% CI), Model 3 | Reference | 1.04 (0.85-1.29) | 1.07 (0.86-1.32) | 0.90 (0.72-1.14) | 1.05 (0.83-1.33) |  | 0.66 |
| Ovarian cancer^¶^ |  |  |  |  |  |  |  |
| Events | 17 | 14 | 21 | 16 | 14 |  |  |
| Person-years | 59168 | 66887 | 70803 | 75911 | 82586 |  |  |
| HR (95% CI), Model 1 | Reference | 0.73 (0.36-1.48) | 1.03 (0.54-1.95) | 0.73 (0.37-1.44) | 0.59 (0.29-1.19) |  | 0.45 |
| HR (95% CI), Model 2 | Reference | 0.74 (0.36-1.50) | 1.06 (0.56-2.01) | 0.75 (0.38-1.48) | 0.60 (0.29-1.21) |  | 0.45 |
| HR (95% CI), Model 3 | Reference | 0.74 (0.36-1.50) | 1.04 (0.54-1.98) | 0.73 (0.37-1.45) | 0.57 (0.28-1.18) |  | 0.41 |
| Breast cancer^¶^ |  |  |  |  |  |  |  |
| Events | 105 | 107 | 125 | 124 | 145 |  |  |
| Person-years | 56324 | 62985 | 66806 | 71596 | 77754 |  |  |
| HR (95% CI), Model 1 | Reference | 0.91 (0.70-1.19) | 1.00 (0.77-1.30) | 0.92 (0.71-1.19) | 1.00 (0.78-1.29) |  | 0.89 |
| HR (95% CI), Model 2 | Reference | 0.91 (0.69-1.19) | 1.00 (0.77-1.30) | 0.93 (0.71-1.20) | 1.00 (0.78-1.29) |  | 0.89 |
| HR (95% CI), Model 3 | Reference | 0.90 (0.69-1.18) | 0.97 (0.75-1.26) | 0.90 (0.69-1.16) | 0.96 (0.74-1.24) |  | 0.90 |
| Other cancer |  |  |  |  |  |  |  |
| Events | 422 | 426 | 425 | 438 | 484 |  |  |
| Person-years | 121963 | 121587 | 121245 | 121478 | 119580 |  |  |
| HR (95% CI), Model 1 | Reference | 1.01 (0.88-1.16) | 1.01 (0.88-1.16) | 1.04 (0.91-1.18) | 1.17 (1.03-1.34) |  | 0.0597 |
| HR (95% CI), Model 2 | Reference | 1.02 (0.89-1.17) | 1.03 (0.90-1.18) | 1.06 (0.93-1.22) | 1.20 (1.05-1.37) |  | 0.0112 |
| HR (95% CI), Model 3 | Reference | 1.02 (0.89-1.18) | 0.98 (0.85-1.12) | 1.03 (0.90-1.18) | 1.15 (1.00-1.32) |  | 0.0830 |
| Depression |  |  |  |  |  |  |  |
| Events | 93 | 94 | 100 | 109 | 106 |  |  |
| Person-years | 119775 | 118986 | 118271 | 116750 | 111840 |  |  |
| HR (95% CI), Model 1 | Reference | 1.00 (0.75-1.33) | 1.07 (0.81-1.42) | 1.19 (0.91-1.58) | 1.21 (0.92-1.60) |  | 0.0870 |
| HR (95% CI), Model 2 | Reference | 0.97 (0.73-1.30) | 1.04 (0.78-1.38) | 1.11 (0.84-1.47) | 1.07 (0.81-1.42) |  | 0.38 |
| HR (95% CI), Model 3 | Reference | 0.94 (0.71-1.25) | 0.98 (0.74-1.30) | 1.02 (0.77-1.35) | 0.92 (0.69-1.23) |  | 0.81 |
| Anxiety |  |  |  |  |  |  |  |
| Events | 300 | 320 | 268 | 307 | 362 |  |  |
| Person-years | 123682 | 123279 | 122732 | 121520 | 117579 |  |  |
| HR (95% CI), Model 1 | Reference | 1.07 (0.91-1.25) | 0.89 (0.76-1.05) | 1.03 (0.88-1.21) | 1.27 (1.09-1.48) |  | 0.0131 |
| HR (95% CI), Model 2 | Reference | 1.02 (0.87-1.19) | 0.85 (0.72-1.00) | 0.96 (0.82-1.12) | 1.10 (0.95-1.29) |  | 0.36 |
| HR (95% CI), Model 3 | Reference | 1.00 (0.85-1.17) | 0.83 (0.70-0.97) | 0.92 (0.78-1.08) | 1.02 (0.87-1.19) |  | 0.84 |
| Schizophrenia |  |  |  |  |  |  |  |
| Events | 14 | 16 | 10 | 16 | 17 |  |  |
| Person-years | 127172 | 127033 | 126239 | 125241 | 121818 |  |  |
| HR (95% CI), Model 1 | Reference | 1.14 (0.56-2.34) | 0.72 (0.32-1.62) | 1.16 (0.56-2.37) | 1.26 (0.62-2.57) |  | 0.54 |
| HR (95% CI), Model 2 | Reference | 1.12 (0.55-2.30) | 0.72 (0.32-1.63) | 1.15 (0.56-2.37) | 1.19 (0.58-2.43) |  | 0.65 |
| HR (95% CI), Model 3 | Reference | 1.07 (0.52-2.21) | 0.66 (0.29-1.49) | 1.04 (0.50-2.15) | 0.96 (0.46-2.03) |  | 0.90 |
| Alcohol problems |  |  |  |  |  |  |  |
| Events | 77 | 73 | 65 | 80 | 94 |  |  |
| Person-years | 126679 | 126652 | 125805 | 124837 | 121359 |  |  |
| HR (95% CI), Model 1 | Reference | 0.95 (0.69-1.30) | 0.85 (0.61-1.18) | 1.05 (0.77-1.44) | 1.26 (0.93-1.70) |  | 0.0722 |
| HR (95% CI), Model 2 | Reference | 1.01 (0.73-1.39) | 0.95 (0.68-1.32) | 1.19 (0.87-1.63) | 1.52 (1.12-2.07) |  | 0.0144 |
| HR (95% CI), Model 3 | Reference | 0.98 (0.71-1.36) | 0.91 (0.65-1.27) | 1.12 (0.81-1.53) | 1.36 (1.00-1.87) |  | 0.0366 |
| Psychoactive substance abuse |  |  |  |  |  |  |  |
| Events | 26 | 22 | 17 | 14 | 19 |  |  |
| Person-years | 127443 | 127304 | 126470 | 125531 | 122121 |  |  |
| HR (95% CI), Model 1 | Reference | 0.85 (0.48-1.49) | 0.66 (0.36-1.21) | 0.55 (0.29-1.05) | 0.76 (0.42-1.38) |  | 0.16 |
| HR (95% CI), Model 2 | Reference | 0.91 (0.52-1.61) | 0.73 (0.40-1.35) | 0.63 (0.33-1.20) | 0.90 (0.50-1.64) |  | 0.38 |
| HR (95% CI), Model 3 | Reference | 0.93 (0.53-1.65) | 0.77 (0.41-1.42) | 0.66 (0.34-1.27) | 0.97 (0.53-1.79) |  | 0.53 |
| Dementia |  |  |  |  |  |  |  |
| Events | 122 | 120 | 112 | 124 | 131 |  |  |
| Person-years | 127397 | 127074 | 126252 | 125483 | 122189 |  |  |
| HR (95% CI), Model 1 | Reference | 0.98 (0.76-1.26) | 0.92 (0.71-1.19) | 1.02 (0.80-1.31) | 1.12 (0.87-1.43) |  | 0.35 |
| HR (95% CI), Model 2 | Reference | 1.03 (0.80-1.32) | 0.99 (0.77-1.29) | 1.16 (0.90-1.49) | 1.24 (0.97-1.59) |  | 0.0553 |
| HR (95% CI), Model 3 | Reference | 1.01 (0.79-1.30) | 0.96 (0.74-1.24) | 1.08 (0.84-1.40) | 1.08 (0.84-1.40) |  | 0.45 |
| Parkinson's disease |  |  |  |  |  |  |  |
| Events | 53 | 48 | 40 | 58 | 43 |  |  |
| Person-years | 127355 | 127212 | 126414 | 125561 | 122445 |  |  |
| HR (95% CI), Model 1 | Reference | 0.91 (0.61-1.34) | 0.76 (0.50-1.15) | 1.11 (0.76-1.61) | 0.84 (0.56-1.26) |  | 0.85 |
| HR (95% CI), Model 2 | Reference | 0.96 (0.65-1.42) | 0.83 (0.55-1.25) | 1.26 (0.87-1.83) | 0.99 (0.66-1.48) |  | 0.55 |
| HR (95% CI), Model 3 | Reference | 0.96 (0.65-1.41) | 0.82 (0.54-1.23) | 1.21 (0.83-1.77) | 0.91 (0.60-1.39) |  | 0.82 |
| Multiple sclerosis |  |  |  |  |  |  |  |
| Events | 6 | 8 | 6 | 5 | 7 |  |  |
| Person-years | 127553 | 127167 | 126395 | 125424 | 122256 |  |  |
| HR (95% CI), Model 1 | Reference | 1.33 (0.46-3.85) | 1.01 (0.32-3.12) | 0.84 (0.26-2.77) | 1.21 (0.41-3.61) |  | 0.96 |
| HR (95% CI), Model 2 | Reference | 1.28 (0.44-3.71) | 0.94 (0.30-2.93) | 0.78 (0.24-2.58) | 1.05 (0.35-3.19) |  | 0.86 |
| HR (95% CI), Model 3 | Reference | 1.28 (0.44-3.71) | 0.98 (0.31-3.04) | 0.80 (0.24-2.67) | 1.08 (0.35-3.32) |  | 0.80 |
| Migraine |  |  |  |  |  |  |  |
| Events | 53 | 44 | 51 | 64 | 58 |  |  |
| Person-years | 123562 | 123490 | 123018 | 121371 | 118025 |  |  |
| HR (95% CI), Model 1 | Reference | 0.83 (0.56-1.24) | 0.96 (0.66-1.42) | 1.23 (0.85-1.76) | 1.12 (0.77-1.63) |  | 0.11 |
| HR (95% CI), Model 2 | Reference | 0.78 (0.53-1.17) | 0.90 (0.61-1.32) | 1.11 (0.77-1.61) | 0.98 (0.67-1.42) |  | 0.48 |
| HR (95% CI), Model 3 | Reference | 0.77 (0.51-1.15) | 0.86 (0.58-1.26) | 1.04 (0.72-1.51) | 0.88 (0.60-1.29) |  | 0.90 |
| Epilepsy |  |  |  |  |  |  |  |
| Events | 40 | 35 | 44 | 38 | 49 |  |  |
| Person-years | 126060 | 125901 | 125534 | 124539 | 121326 |  |  |
| HR (95% CI), Model 1 | Reference | 0.88 (0.56-1.38) | 1.10 (0.72-1.69) | 0.93 (0.60-1.46) | 1.27 (0.84-1.93) |  | 0.22 |
| HR (95% CI), Model 2 | Reference | 0.91 (0.58-1.43) | 1.17 (0.76-1.80) | 1.04 (0.67-1.62) | 1.39 (0.91-2.13) |  | 0.10 |
| HR (95% CI), Model 3 | Reference | 0.90 (0.57-1.42) | 1.16 (0.75-1.78) | 1.02 (0.65-1.59) | 1.33 (0.86-2.04) |  | 0.18 |
| COPD |  |  |  |  |  |  |  |
| Events | 230 | 242 | 222 | 243 | 273 |  |  |
| Person-years | 124962 | 124247 | 123458 | 122142 | 118475 |  |  |
| HR (95% CI), Model 1 | Reference | 1.07 (0.89-1.28) | 0.99 (0.82-1.19) | 1.08 (0.90-1.30) | 1.23 (1.03-1.47) |  | 0.0311 |
| HR (95% CI), Model 2 | Reference | 1.13 (0.94-1.35) | 1.08 (0.90-1.30) | 1.19 (1.00-1.43) | 1.37 (1.15-1.64) |  | 0.0006 |
| HR (95% CI), Model 3 | Reference | 1.11 (0.92-1.33) | 1.05 (0.87-1.26) | 1.14 (0.95-1.37) | 1.25 (1.04-1.49) |  | 0.0233 |
| Asthma |  |  |  |  |  |  |  |
| Events | 147 | 157 | 141 | 143 | 184 |  |  |
| Person-years | 112013 | 112027 | 111717 | 109344 | 104806 |  |  |
| HR (95% CI), Model 1 | Reference | 1.07 (0.85-1.34) | 0.95 (0.75-1.19) | 0.99 (0.78-1.24) | 1.33 (1.07-1.65) |  | 0.0361 |
| HR (95% CI), Model 2 | Reference | 1.06 (0.85-1.33) | 0.96 (0.76-1.21) | 0.98 (0.78-1.23) | 1.29 (1.04-1.61) |  | 0.0659 |
| HR (95% CI), Model 3 | Reference | 1.04 (0.83-1.30) | 0.91 (0.72-1.15) | 0.92 (0.73-1.16) | 1.15 (0.92-1.44) |  | 0.49 |
| Bronchiectasis |  |  |  |  |  |  |  |
| Events | 78 | 59 | 68 | 62 | 61 |  |  |
| Person-years | 127214 | 126986 | 126150 | 125266 | 122011 |  |  |
| HR (95% CI), Model 1 | Reference | 0.76 (0.54-1.06) | 0.88 (0.63-1.21) | 0.79 (0.57-1.11) | 0.79 (0.56-1.10) |  | 0.33 |
| HR (95% CI), Model 2 | Reference | 0.76 (0.54-1.06) | 0.89 (0.65-1.24) | 0.82 (0.59-1.15) | 0.83 (0.59-1.16) |  | 0.39 |
| HR (95% CI), Model 3 | Reference | 0.77 (0.55-1.08) | 0.92 (0.66-1.28) | 0.85 (0.61-1.19) | 0.87 (0.62-1.23) |  | 0.60 |
| Dyspepsia |  |  |  |  |  |  |  |
| Events | 842 | 822 | 833 | 843 | 923 |  |  |
| Person-years | 106379 | 105434 | 105370 | 105210 | 99523 |  |  |
| HR (95% CI), Model 1 | Reference | 0.98 (0.89-1.08) | 0.99 (0.90-1.10) | 1.01 (0.91-1.11) | 1.17 (1.07-1.29) |  | 0.0026 |
| HR (95% CI), Model 2 | Reference | 0.98 (0.89-1.08) | 1.00 (0.90-1.10) | 1.01 (0.91-1.11) | 1.15 (1.05-1.27) |  | 0.0045 |
| HR (95% CI), Model 3 | Reference | 0.97 (0.88-1.06) | 0.96 (0.88-1.06) | 0.96 (0.87-1.06) | 1.06 (0.96-1.17) |  | 0.33 |
| Constipation |  |  |  |  |  |  |  |
| Events | 348 | 296 | 274 | 341 | 347 |  |  |
| Person-years | 124269 | 124573 | 123315 | 122676 | 118677 |  |  |
| HR (95% CI), Model 1 | Reference | 0.85 (0.73-1.00) | 0.79 (0.67-0.92) | 1.00 (0.86-1.16) | 1.04 (0.90-1.21) |  | 0.22 |
| HR (95% CI), Model 2 | Reference | 0.85 (0.73-0.99) | 0.80 (0.69-0.94) | 1.01 (0.87-1.18) | 1.05 (0.90-1.22) |  | 0.13 |
| HR (95% CI), Model 3 | Reference | 0.83 (0.71-0.97) | 0.77 (0.66-0.91) | 0.95 (0.82-1.11) | 0.95 (0.81-1.11) |  | 0.89 |
| Diverticulitis |  |  |  |  |  |  |  |
| Events | 654 | 651 | 653 | 701 | 708 |  |  |
| Person-years | 119378 | 118830 | 117589 | 116549 | 113113 |  |  |
| HR (95% CI), Model 1 | Reference | 1.00 (0.90-1.11) | 1.01 (0.91-1.12) | 1.10 (0.99-1.22) | 1.14 (1.03-1.27) |  | 0.0058 |
| HR (95% CI), Model 2 | Reference | 1.00 (0.90-1.11) | 1.01 (0.91-1.13) | 1.10 (0.99-1.22) | 1.13 (1.02-1.26) |  | 0.0054 |
| HR (95% CI), Model 3 | Reference | 0.98 (0.88-1.09) | 0.97 (0.87-1.09) | 1.04 (0.93-1.16) | 1.03 (0.92-1.15) |  | 0.32 |
| Irritable bowel syndrome |  |  |  |  |  |  |  |
| Events | 103 | 78 | 106 | 88 | 118 |  |  |
| Person-years | 124135 | 123487 | 122352 | 121683 | 118087 |  |  |
| HR (95% CI), Model 1 | Reference | 0.76 (0.57-1.02) | 1.04 (0.80-1.37) | 0.86 (0.65-1.15) | 1.21 (0.93-1.57) |  | 0.15 |
| HR (95% CI), Model 2 | Reference | 0.71 (0.53-0.96) | 0.95 (0.72-1.24) | 0.77 (0.58-1.02) | 0.99 (0.76-1.29) |  | 0.83 |
| HR (95% CI), Model 3 | Reference | 0.70 (0.52-0.95) | 0.93 (0.71-1.22) | 0.74 (0.56-0.99) | 0.92 (0.70-1.21) |  | 0.73 |
| Inflammatory bowel disease |  |  |  |  |  |  |  |
| Events | 49 | 41 | 37 | 44 | 42 |  |  |
| Person-years | 126298 | 126028 | 125238 | 124126 | 120978 |  |  |
| HR (95% CI), Model 1 | Reference | 0.84 (0.55-1.27) | 0.76 (0.50-1.16) | 0.91 (0.61-1.37) | 0.89 (0.59-1.35) |  | 0.74 |
| HR (95% CI), Model 2 | Reference | 0.85 (0.56-1.29) | 0.79 (0.52-1.22) | 0.97 (0.64-1.46) | 0.95 (0.63-1.44) |  | 0.99 |
| HR (95% CI), Model 3 | Reference | 0.85 (0.56-1.29) | 0.78 (0.51-1.20) | 0.96 (0.63-1.45) | 0.93 (0.61-1.43) |  | 0.93 |
| Chronic liver disease |  |  |  |  |  |  |  |
| Events | 22 | 22 | 27 | 25 | 42 |  |  |
| Person-years | 126729 | 126648 | 125802 | 124630 | 121406 |  |  |
| HR (95% CI), Model 1 | Reference | 1.05 (0.58-1.90) | 1.29 (0.73-2.28) | 1.20 (0.67-2.15) | 2.03 (1.20-3.43) |  | 0.0069 |
| HR (95% CI), Model 2 | Reference | 1.04 (0.58-1.88) | 1.31 (0.74-2.30) | 1.25 (0.70-2.22) | 2.19 (1.30-3.70) |  | 0.0020 |
| HR (95% CI), Model 3 | Reference | 1.01 (0.56-1.83) | 1.24 (0.70-2.18) | 1.13 (0.63-2.02) | 1.78 (1.04-3.06) |  | 0.0300 |
| CKD |  |  |  |  |  |  |  |
| Events | 415 | 409 | 467 | 536 | 650 |  |  |
| Person-years | 125486 | 125183 | 124003 | 122803 | 117954 |  |  |
| HR (95% CI), Model 1 | Reference | 0.99 (0.87-1.14) | 1.14 (0.99-1.30) | 1.33 (1.17-1.51) | 1.68 (1.48-1.90) |  | <0.0001 |
| HR (95% CI), Model 2 | Reference | 1.02 (0.89-1.17) | 1.22 (1.07-1.40) | 1.45 (1.27-1.65) | 1.86 (1.64-2.11) |  | <0.0001 |
| HR (95% CI), Model 3 | Reference | 0.97 (0.84-1.11) | 1.10 (0.96-1.26) | 1.22 (1.07-1.39) | 1.40 (1.23-1.59) |  | <0.0001 |
| Osteoporosis |  |  |  |  |  |  |  |
| Events | 524 | 539 | 569 | 545 | 595 |  |  |
| Person-years | 115210 | 114803 | 114062 | 113716 | 112144 |  |  |
| HR (95% CI), Model 1 | Reference | 1.03 (0.91-1.16) | 1.10 (0.98-1.24) | 1.05 (0.93-1.19) | 1.17 (1.04-1.31) |  | 0.0321 |
| HR (95% CI), Model 2 | Reference | 1.02 (0.90-1.15) | 1.08 (0.96-1.21) | 1.02 (0.90-1.15) | 1.10 (0.98-1.24) |  | 0.0813 |
| HR (95% CI), Model 3 | Reference | 0.97 (0.86-1.10) | 0.99 (0.88-1.12) | 0.91 (0.81-1.03) | 0.96 (0.84-1.08) |  | 0.30 |
| Fracture |  |  |  |  |  |  |  |
| Events | 83 | 78 | 76 | 87 | 88 |  |  |
| Person-years | 130856 | 131249 | 131156 | 131440 | 131118 |  |  |
| HR (95% CI), Model 1 | Reference | 0.94 (0.69-1.27) | 0.90 (0.66-1.23) | 1.04 (0.77-1.41) | 1.05 (0.77-1.41) |  | 0.71 |
| HR (95% CI), Model 2 | Reference | 0.90 (0.66-1.23) | 0.87 (0.64-1.19) | 0.99 (0.73-1.34) | 0.97 (0.71-1.31) |  | 0.90 |
| HR (95% CI), Model 3 | Reference | 0.88 (0.64-1.21) | 0.88 (0.64-1.21) | 0.98 (0.72-1.34) | 0.93 (0.68-1.29) |  | 0.98 |
| Glaucoma |  |  |  |  |  |  |  |
| Events | 164 | 146 | 147 | 162 | 163 |  |  |
| Person-years | 129310 | 130001 | 129697 | 129792 | 129482 |  |  |
| HR (95% CI), Model 1 | Reference | 0.87 (0.69-1.08) | 0.87 (0.70-1.09) | 0.98 (0.79-1.22) | 0.98 (0.79-1.22) |  | 0.93 |
| HR (95% CI), Model 2 | Reference | 0.89 (0.71-1.11) | 0.90 (0.72-1.12) | 1.00 (0.80-1.24) | 0.99 (0.79-1.23) |  | 0.77 |
| HR (95% CI), Model 3 | Reference | 0.92 (0.73-1.16) | 0.92 (0.73-1.17) | 1.01 (0.80-1.27) | 0.98 (0.77-1.24) |  | 0.89 |
| Cataract |  |  |  |  |  |  |  |
| Events | 707 | 727 | 744 | 727 | 779 |  |  |
| Person-years | 124021 | 124198 | 124331 | 124018 | 122749 |  |  |
| HR (95% CI), Model 1 | Reference | 1.02 (0.92-1.14) | 1.04 (0.94-1.15) | 1.02 (0.92-1.13) | 1.11 (1.00-1.23) |  | 0.57 |
| HR (95% CI), Model 2 | Reference | 1.03 (0.92-1.14) | 1.04 (0.94-1.16) | 1.02 (0.92-1.13) | 1.07 (0.97-1.19) |  | 0.82 |
| HR (95% CI), Model 3 | Reference | 1.02 (0.91-1.13) | 1.03 (0.93-1.15) | 0.96 (0.86-1.07) | 0.95 (0.85-1.06) |  | 0.15 |
| AMD |  |  |  |  |  |  |  |
| Events | 110 | 108 | 123 | 112 | 159 |  |  |
| Person-years | 130940 | 131260 | 131456 | 131273 | 130931 |  |  |
| HR (95% CI), Model 1 | Reference | 0.98 (0.75-1.28) | 1.10 (0.85-1.43) | 1.01 (0.78-1.32) | 1.43 (1.12-1.82) |  | 0.0115 |
| HR (95% CI), Model 2 | Reference | 0.97 (0.74-1.27) | 1.10 (0.85-1.42) | 0.99 (0.76-1.28) | 1.37 (1.07-1.75) |  | 0.0198 |
| HR (95% CI), Model 3 | Reference | 0.95 (0.72-1.25) | 1.10 (0.84-1.43) | 0.97 (0.74-1.28) | 1.28 (0.98-1.66) |  | 0.0701 |
| Pernicious anaemia |  |  |  |  |  |  |  |
| Events | 14 | 13 | 19 | 10 | 16 |  |  |
| Person-years | 127341 | 127162 | 126372 | 125526 | 122139 |  |  |
| HR (95% CI), Model 1 | Reference | 0.93 (0.44-1.98) | 1.29 (0.64-2.60) | 0.72 (0.32-1.63) | 1.19 (0.58-2.44) |  | 0.87 |
| HR (95% CI), Model 2 | Reference | 0.90 (0.42-1.93) | 1.37 (0.68-2.74) | 0.71 (0.31-1.60) | 1.12 (0.54-2.32) |  | 0.96 |
| HR (95% CI), Model 3 | Reference | 0.88 (0.43-1.78) | 1.33 (0.70-2.53) | 0.76 (0.37-1.56) | 0.81 (0.40-1.64) |  | 0.69 |
| Thyroid disorders |  |  |  |  |  |  |  |
| Events | 158 | 184 | 192 | 215 | 245 |  |  |
| Person-years | 124006 | 123153 | 123390 | 122029 | 120178 |  |  |
| HR (95% CI), Model 1 | Reference | 1.17 (0.95-1.45) | 1.21 (0.98-1.49) | 1.36 (1.11-1.67) | 1.60 (1.31-1.95) |  | <0.0001 |
| HR (95% CI), Model 2 | Reference | 1.10 (0.89-1.37) | 1.13 (0.92-1.40) | 1.25 (1.02-1.53) | 1.35 (1.11-1.66) |  | 0.0027 |
| HR (95% CI), Model 3 | Reference | 1.06 (0.85-1.31) | 1.04 (0.83-1.29) | 1.16 (0.94-1.43) | 1.17 (0.95-1.45) |  | 0.0843 |
| Eczema |  |  |  |  |  |  |  |
| Events | 83 | 104 | 111 | 95 | 114 |  |  |
| Person-years | 122553 | 122298 | 121450 | 120297 | 116837 |  |  |
| HR (95% CI), Model 1 | Reference | 1.25 (0.94-1.67) | 1.34 (1.01-1.78) | 1.15 (0.86-1.55) | 1.43 (1.08-1.90) |  | 0.0727 |
| HR (95% CI), Model 2 | Reference | 1.26 (0.94-1.68) | 1.36 (1.02-1.81) | 1.18 (0.88-1.59) | 1.41 (1.06-1.88) |  | 0.0562 |
| HR (95% CI), Model 3 | Reference | 1.22 (0.91-1.63) | 1.28 (0.96-1.71) | 1.08 (0.80-1.46) | 1.23 (0.92-1.65) |  | 0.40 |
| Meniere disease |  |  |  |  |  |  |  |
| Events | 14 | 7 | 13 | 5 | 8 |  |  |
| Person-years | 131364 | 131587 | 131742 | 131659 | 131582 |  |  |
| HR (95% CI), Model 1 | Reference | 0.50 (0.20-1.24) | 0.93 (0.44-1.98) | 0.36 (0.13-0.99) | 0.58 (0.24-1.37) |  | 0.16 |
| HR (95% CI), Model 2 | Reference | 0.49 (0.20-1.22) | 0.93 (0.44-1.99) | 0.36 (0.13-1.00) | 0.55 (0.23-1.33) |  | 0.17 |
| HR (95% CI), Model 3 | Reference | 0.43 (0.16-1.14) | 0.84 (0.38-1.87) | 0.27 (0.09-0.83) | 0.39 (0.15-1.04) |  | 0.0424 |
| Prostate disorders^‡^ |  |  |  |  |  |  |  |
| Events | 347 | 316 | 255 | 268 | 219 |  |  |
| Person-years | 64792 | 58453 | 54395 | 50360 | 43974 |  |  |
| HR (95% CI), Model 1 | Reference | 1.01 (0.87-1.18) | 0.87 (0.74-1.03) | 0.99 (0.84-1.16) | 0.92 (0.78-1.10) |  | 0.37 |
| HR (95% CI), Model 2 | Reference | 1.02 (0.87-1.18) | 0.88 (0.75-1.04) | 0.99 (0.85-1.16) | 0.90 (0.76-1.07) |  | 0.33 |
| HR (95% CI), Model 3 | Reference | 1.00 (0.86-1.16) | 0.85 (0.72-1.00) | 0.95 (0.81-1.12) | 0.84 (0.71-1.01) |  | 0.11 |

AMD, age related macular degeneration; CI, confidence interval; CKD, chronic kidney disease; COPD, chronic obstructive pulmonary disease; HR, hazard ratio.

*Age gap was calculated by subtracting chronological age from metabolomic age. Chronological age-adjusted age gap was calculated with use of regression models.

^†^Cox proportional regression models were used to examine the association between chronological age-adjusted age gap and incidence of individual chronic diseases by excluding those developed the disease in the first 5 years of follow-up. Model 1 was unadjusted; Model 2 was adjusted for Model 1 plus age, sex, ethnicity, education, household income, diet score, alcohol consumption, physical activity, smoking, sleep duration, fasting duration, and GRS for longevity; Model 3 was adjusted for Model 2 plus BMI, high cholesterol, hypertension, and antihypertensive and lipid-lowering medications.

^‡^These analyses were conducted among men only.

^¶^These analyses were conducted among women only.
